# Engineering MXenes for Thermal and Photothermal Catalysis

**DOI:** 10.1021/acs.chemrev.5c00705

**Published:** 2026-03-09

**Authors:** Aicha Anouar, Amarajothi Dhakshinamoorthy, Feiyan Xu, Sergio Navalon, Ana Primo, Jiaguo Yu, Hermenegildo Garcia

**Affiliations:** † Instituto Universitario de Tecnología Química, Consejo Superior de Investigaciones Científicas-Universitat Politecnica de Valencia, 16774 Universitat Politecnica de Valencia, Av. De los Naranjos s/n, Valencia 46022, Spain; ‡ School of Chemistry, 29944 Madurai Kamaraj University, Madurai 625021, Tamil Nadu, India; § Departamento de Química, Universitat Politècnica de València, C/Camino de Vera, s/n, 46022 Valencia, Spain; ∥ Laboratory of Solar Fuel, Faculty of Materials Science and Chemistry, 12564 China University of Geosciences, Wuhan 430078, P. R. China

## Abstract

Heterogeneous catalysis relies on advanced, tunable materials offering structurally defined active sites and large accessible surface areas. Among the various material types, two-dimensional nanomaterials with high aspect ratios feature a high fraction of exposed atoms and thus efficient atom utilization. After more than a decade since the first report of MXene synthesis, these two-dimensional transition-metal carbides and nitrides, composed of alternating one-atom-thick metal and carbide/nitride layers with surface terminations, have found applications in diverse catalytic areas. This review focuses on the use of MXenes as solid catalysts in thermal or photothermal reactions, while electro- and photocatalysis are excluded as they have been extensively reviewed elsewhere. Section 2 briefly summarizes MXene synthesis and structural features, followed by Section 3 describing the nature and characterization of catalytically active sites, including surface groups, vacancies, and metal–support interfaces that arise from the synthesis conditions. Section 4 emphasizes best practices for ensuring reproducible and stable catalytic performance, with turnover frequency as a key comparative metric. Sections 5 and 6 highlight some representative thermal and photothermal reactions, underscoring the high light-to-heat conversion efficiency of MXenes. This review concludes with current challenges and future prospects, anticipating rapid progress with MXene-based heterogeneous catalysis.

## Graphical abstract

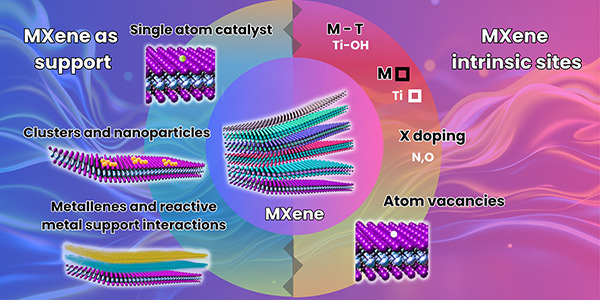

## 1 Introduction

### 1.1 MXenes as Emerging Materials in Heterogeneous Catalysis

Heterogeneous catalysis, in which the active sites responsible for promoting a chemical reaction are in a different phase than the substrates or products, is a foundational pillar of chemical science and has played a central role in the advancement of modern chemical industry and environmental technologies.^1^ Compared to homogeneous catalysis, heterogeneous catalysis is particularly valued for its robustness, ease of catalyst recovery, scalability, and straightforward implementation in continuous flow processes. These advantages have made heterogeneous catalysis the enabling technology for the large-scale synthesis of chemicals and fuels, as well as for applications in environmental remediation and energy conversion. The significance of heterogeneous catalysis in the industrial, energy, and environmental sectors cannot be overstated. It is estimated that 80–90% of all chemical manufacturing processes involve at least one catalytic step, with the majority relying on heterogeneous catalysts. Prominent examples include oil refining, the Haber–Bosch ammonia (NH_3_) synthesis, the Fischer–Tropsch process for converting syngas into hydrocarbons, and catalytic converters for reducing automobile emissions. These transformations are typically mediated by solid materials that offer active sites for reagents, enhancing selectivity, efficiency, and overall economic viability.

In addition to solid acids and bases, most heterogeneous catalysts are composed of transition metal compounds.^2^ Owing to their electronic configuration, transition metals function effectively as Lewis acids and facilitate bond activation through electron transfer processes. Metallic catalysts such as platinum, palladium, and nickel are widely employed in hydrogenation, dehydrogenation, and reforming reactions. Transition metal oxides like TiO_2_, V_2_O_5_, and CeO_2_ are prevalent in oxidation reactions and environmental catalysis,^3^ while sulfides such as MoS_2_ are central to hydrodesulfurization in oil refining.^4^ Transition metal carbides and nitrides such as Mo_2_C and W_2_C have gained attention for exhibiting noble metal-like catalytic properties, but at lower cost with high thermal stability.^5^ These catalytic systems have undergone decades of research and optimization, achieving high activity, selectivity, and durability under industrial conditions.

Heterogeneous catalysis is inherently multidisciplinary, benefiting from advances in material sciences, computational chemistry, applied spectroscopy, chemical physics, and engineering (Figure 1). Progress in any one of these disciplines has an inevitable impact on catalytic science. For example, advances in high-resolution microscopy enabled the visualization of nanoscale features, leading to a clear correlation between nanoparticle (NP) size and catalytic activity.^6,7^ A well-known case is Au catalysis.^8^ Although Au was long considered catalytically inert, Haruta’s pioneering work demonstrated that Au NPs smaller than 10 nm exhibit remarkable activity for low-temperature CO oxidation.^9^

**Figure 1:**
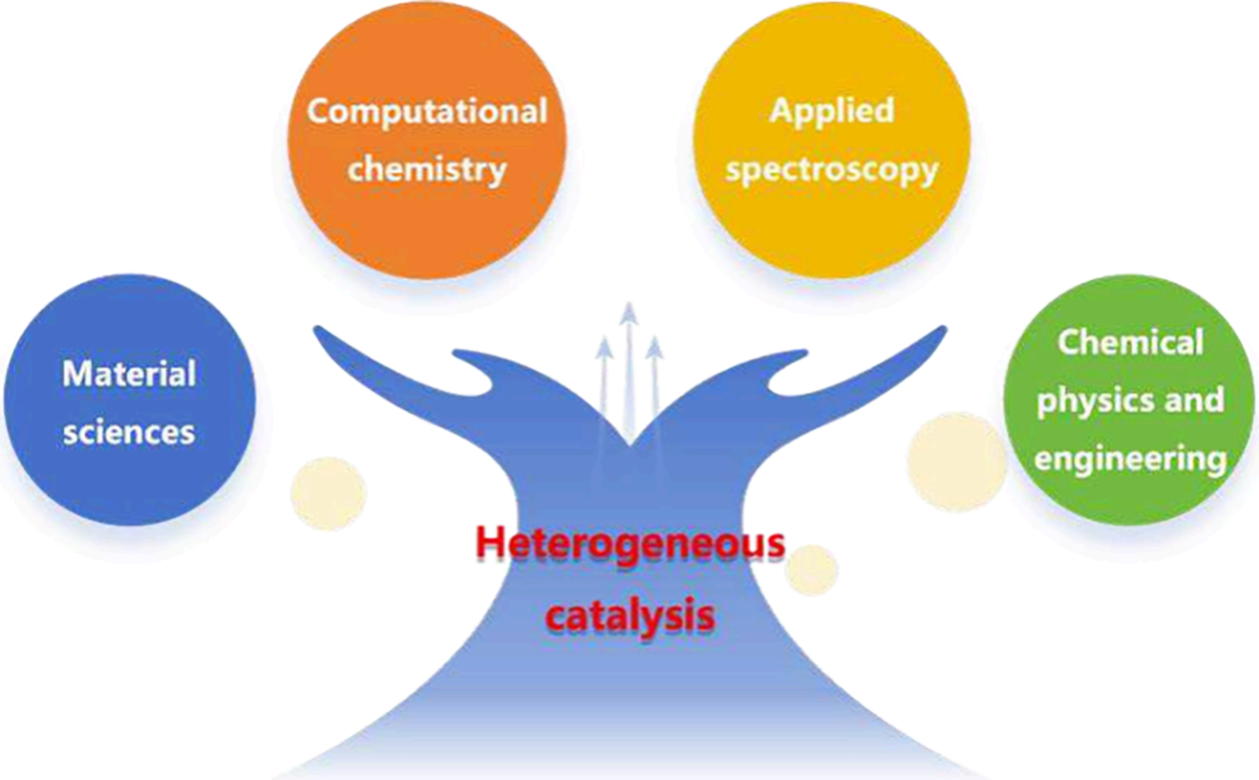
Multidisciplinary contributions to the development of heterogeneous catalysis.

Another example is the emergence of two-dimensional (2D) nanomaterials, which opened new frontiers in heterogeneous catalysis by offering distinctive advantages over their three-dimensional (3D) counterparts.^10^ With their high aspect ratios and ultrathin architectures, 2D nanomaterials provide a large density of exposed active sites per unit mass, high atomic utilization, and superior mass transport.^11^ Unlike porous 3D solids, where reactants must diffuse through lengthy pore networks, 2D materials allow rapid access to active sites.^12^ Moreover, their tunable surface chemistry, mechanical flexibility, and ability to integrate into layered architectures allow precise control over reaction pathways and product selectivity. Their thin morphology also makes 2D materials ideal for nonthermal energy inputs, such as light or electric fields, which can penetrate these structures more effectively than bulk materials.^13^ Consequently, 2D materials are increasingly explored for applications in electro- and photocatalysis.

Among 2D materials, MXenes have rapidly emerged as a particularly promising class due to their unique combination of properties.^14^ Discovered in 2011 by selective etching of layered MAX phases,^15^ MXenes are 2D carbides, nitrides, or carbonitrides of early transition metals, typically described by the formula M_*n*+1_X_*n*_T_*x*_, where M represents an early transition metal (e.g., Sc, Ti, V, Zr and Mo), X is carbon and/or nitrogen, T_*x*_ denotes surface terminations, in most of the cases determined by the etching process, and *n* ranges from 1 to 4. MXenes consist of alternating atom-thick layers of M and X, with M atoms forming the outermost layers and being terminated with surface groups such as −F, −O, or −OH, depending on the etching method (Figure 2). The remarkable versatility of MXenes stems from the interplay between their atomically thin, metallic M_*n*+1_X_*n*_ cores and the diverse surface terminations (T_*x*_). In the pristine carbide or nitride lattice, early transition metals form strong covalent and metallic M–X bonds that stabilize a hexagonal close packed stacking. Although bare metal surfaces of MXenes have occasionally been reported, the low oxidation state of the metal atoms in this circumstance makes them highly reactive and susceptible to oxidation if exposed to the ambient.^16^

**Figure 2:**
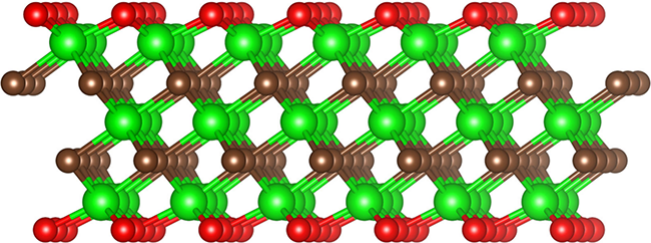
Structure of MXene material (M_3_X_2_T_2_). Green spheres represent early transition metal atoms (M), brown spheres denote carbon or nitrogen atoms (X), and red spheres indicate surface termination groups (T).

What immediately attracted the interest to MXenes in materials science was their unique combination of metallic conductivity, hydrophilicity, and surface functionalization, three properties rarely found together in graphene-like materials.^17^ These features, together with their vast chemical space and composition, grant MXenes a level of structural and chemical tunability that makes them ideally suited for catalytic design. Without considering surface terminations, to date, over 70 MXenes have been reported, and this number continues to grow with the development of multimetal compositions and nonstoichiometric alloys such as Mo_2_TiC_2_ and Ti–V/Nb solid solutions.^18^

In just a few years since their discovery, MXenes have demonstrated exceptional performance across a wide range of applications, particularly in the field of renewable energy. Their high electrical conductivity and redox-active surfaces make them excellent electrode materials for lithium-ion batteries, sodium-ion batteries, and beyond.^19^ In electrochemical energy storage, MXene-based supercapacitors have shown remarkable capacitance and cycling stability, benefiting from their layered structure and rapid ion intercalation.^20^ Furthermore, MXenes are actively being investigated for their photocatalytic and electrocatalytic capabilities, especially in reactions such as water splitting, CO_2_ reduction, and nitrogen fixation—processes that are central to sustainable energy production and environmental remediation.^21^ In contrast to the excitement surrounding MXenes in electrocatalysis and their growing use in photocatalysis, relatively little attention has been paid to their potential in conventional thermal catalysis.^22^ This limited interest is particularly striking given that, as previously noted, transition metal compounds, including early transition elements, are among the most widely used catalysts, with broad applicability in Lewis acids and redox reactions. Figure 3 illustrates the potential application of MXenes in various fields.

**Figure 3:**
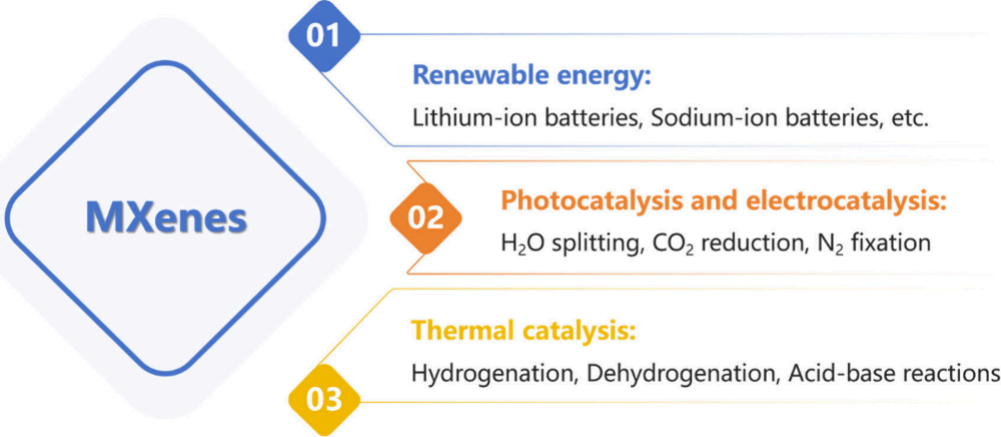
Potential applications of MXenes as solid catalysts.

Considering the potential applicability of MXenes as thermal catalysts and taking into account their structure and composition, one can expect them to exhibit catalytic properties comparable to those of transition metal carbides, which are well-known heterogeneous catalysts for hydrogenations and hydroprocessing reactions, including NH_3_ synthesis and decomposition, CO_2_ hydrogenation, as well as reductive C–C and C–X bond cleavage.^23^ Transition metal carbides are also increasingly used in biomass conversion for the hydrotreatment of vegetable oils and lignin depolymerization.^5^ Thus, in view of the composition and structure of MXenes, as well as the presence of structural defects, a correlation between these features and their catalytic activity can be anticipated based on current knowledge on the nature of active sites in other similar materials. It is therefore expected that all these reactions could also be promoted by suitably modified MXenes.

In addition, considering the surface functional groups that are almost universally present in ambient-equilibrated MXenes, a clear analogy can be drawn between the metal coordination environments in MXenes and the active sites of early transition metals in conventional heterogeneous catalysts. For example, titanol groups (≡Ti–OH), which can undergo ligand exchange with −OOH or −OOR, are recognized as active sites in titanosilicates for reactions such as alkene epoxidation and aromatic hydroxylation.^24^ Similarly, vanadyl (≡V=O) anchored on silica are active centers in hydrocarbon oxidation.^25^ Another example is Nb=O moieties, which function as water-tolerant Lewis acids capable of promoting glucose dehydration to hydroxymethylfurfural.^26^ Such ≡M–O­(OH) coordination motifs are also present in MXenes, due to their universal oxygenated surface terminations. However, it is reasonable to expect that their behavior may differ from those in classical metal oxides, as the underlying carbide/nitride layers in MXenes contribute significantly to the electron density of the metal center. This higher electron density must alter reactivity, but also opens the possibility of fine-tuning the catalytic behavior by modifying the electron-withdrawing nature and spatial distribution of the surface termination groups. In addition, these MXene surface functional groups will experience rigidity and steric constrains that surely would influence their activity.

Another source of inspiration suggesting a broad applicability of MXenes in heterogeneous catalysis comes from homogeneous catalysis, particularly from the well-documented activity of molecular organometallic complexes of early transition metals. These complexes often feature metal centers bonded to negatively charged carbons, such as cyclopentadienylium.^27^ Such compounds are very well-known catalysts for a wide range of chemical transformations, including alkene polymerization,^28^ alkyne oligomerization,^29^ and various C–C bond-forming reactions such as hydroaminations^30^ and hydrosilylations.^31^ Representative examples of these catalytically active organometallic complexes are presented in Figure 4.

**Figure 4:**
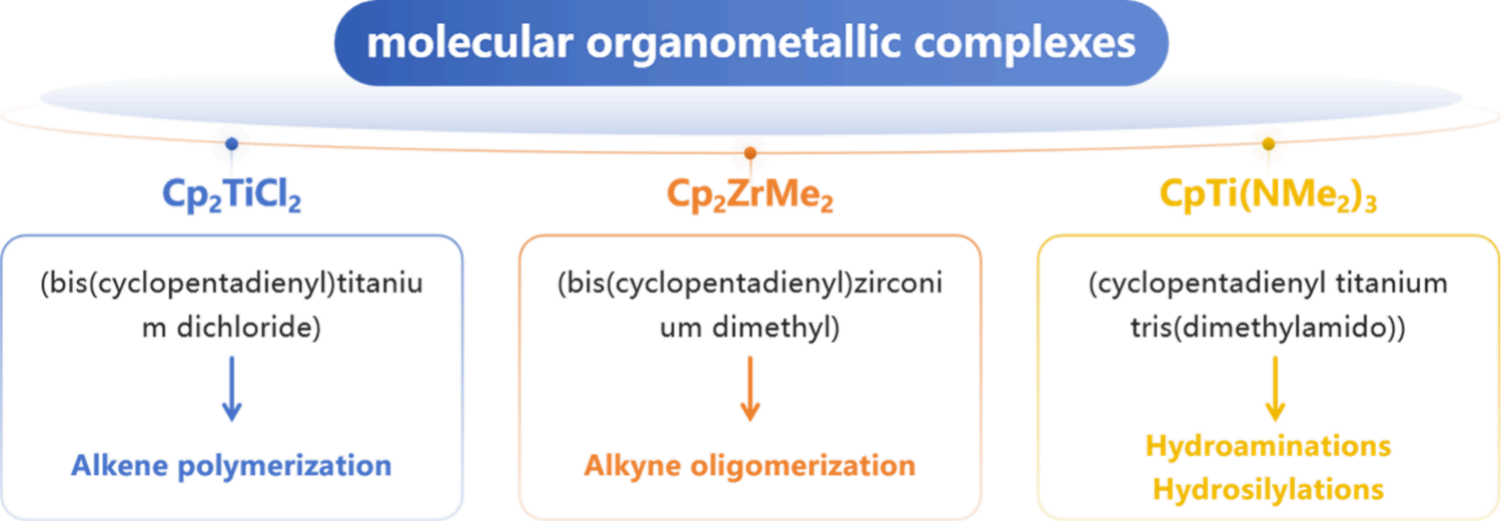
Organometallic complexes of early transition metals as inspiration for MXene-based heterogeneous catalysis.

In all the above considerations regarding the catalytic potential of MXenes, it is important to note that their composition, particularly the nature of the surface terminations, and potentially the alloying with other metals, offers a degree of tunability in the electronic density at the presumed active sites. This tunability can, in principle, be exploited to tailor the properties of a given MXene to meet the specific electronic and geometric requirements of a particular reaction mechanism.

One major point of concern when proposing MXenes for heterogeneous catalysis is their limited stability under certain solvents and reaction conditions.^32^ As with any catalyst, stability under operating conditions is a prerequisite for practical applications. While MXenes are generally thermally stable and can withstand heating under inert atmosphere up to 700 °C, structural and compositional changes may occur at higher temperatures, including phase transformation into bulk 3D compounds.^33^ Even below this transformation threshold, surface modification and other thermally induced processes may take place,^34^ potentially altering the nature and performance of the active sites.

Due to the nature of carbides or nitrides containing metal atoms in low oxidation states, MXenes are generally stable under reductive conditions. In contrast, they are prone to oxidation and therefore less suitable for promoting oxidative reactions. Early transition metals are strongly oxyphilic, and their corresponding oxides are thermodynamically stable. It has been reported that Ti_3_C_2_ and other MXenes undergo spontaneous oxidation when suspended in aqueous media exposed to air, gradually converting into the corresponding metal oxides.^35^ MXenes synthesized via fluorinated etching reagents exhibit intrinsic hydrophilicity, primarily due to the random distribution of surface terminations such as −OH, −O, and −F, which enables their dispersion in water. To mitigate oxidative degradation, MXene suspensions are typically stored under inert atmosphere. However, even under such conditions, gradual oxidation over time still occurs, and this process is significantly accelerated at elevated temperatures.^36^ The hydrophilicity of MXenes also increases in the presence of structural defects, such as vacancies and flake edges, which further facilitate oxidation. This degradation is particularly rapid in MXenes with the minimum possible number of layers, such as Ti_2_C, while it tends to be less severe in multilayered MXenes.

In addition to oxidation, the chemical stability of MXenes is also influenced by the pH of the aqueous environment. Generally, MXenes remain relatively stable at ambient temperature in mildly acidic to neutral solutions (pH 3–7), whereas strongly acidic or alkaline conditions accelerate the dissolution of surface terminations (−OH, −F) and even induce partial leaching of metal atoms or lattice degradation with formation of metal oxides. These acid or basic attacks increase upon the elapsed time and with the temperature. For example, Ti_3_C_2_T_*x*_ gradually decomposes in concentrated alkaline media due to hydroxide attack, while in strong acids partial dissolution of Ti layers has been observed.^37^ Under hydrothermal conditions and high saline concentration, partial oxidation of MXenes can also occur.^38^ Therefore, maintaining a moderate pH environment is essential for preserving the structural integrity and catalytic performance of MXenes in aqueous systems.

Hydrothermal treatment of MXenes in saline aqueous solutions also leads to their oxidation into the corresponding metal oxides.^39,40^ By controlling the treatment duration, it is possible to modulate the extent of conversion.^41^ In contrast, storing MXene inks in water under an inert atmosphere appears to suppress this undesired oxidation. Exposure of MXenes to oxidizing agents such as hydroperoxides (e.g., monoperoxypersulfate) at ambient temperature for durations ranging from a few min to several hours initiates modification of the surface groups via the incorporation of O atoms.^42^ Prolonged exposure eventually results in the full conversion of MXenes into metal oxides. One study has linked the instability of MXenes in suspension to the dielectric constant of the solvent.^43^ Although partial or complete transformation into metal oxides maybe attractive for certain applications, for example through retention of 2D morphology or the formation of strongly interacting heterojunctions derived from MXene precursors, such oxidation is generally undesirable in catalysis. Therefore, it can be concluded that, in principle, MXenes are not ideal materials for oxidation catalysis. One of the most resistant MXenes against oxidation is Mo_2_CT_*x*_, but also Nb_2_CT_*x*_ remains intact to ∼600 °C. In the case of Mo_2_TiC_2_T_*x*_ it has been assumed^33^ that its stability against oxidation is similar to that of Ti_3_C_2_T_*x*_, (285.6 °C in air by thermogravimetry analysis (TGA)),^44^ but this assumption still needs experimental data. In addition, oxidation onsets can be shifted even 100 °C higher by replacing −F with −Cl/–O or by encapsulating flakes in inert polymers. In the case of Mo_2_TiC_2_, TGA data in air shows that this bimetallic MXene does not undergo oxidation up to a temperature of 350 °C, with oxidation not becoming significant until well above this temperature. In this way, Mo_2_TiC_2_ exceeds the stability of many other MXenes and sets the basis for even further resistance to oxidation. Figure 5 shows a plot illustrating the stability of Mo_2_TiC_2_ oxidation. It should be noted, however, that this thermal analysis was conducted under continuous heating at a constant rate. As such, the observed oxidation onset primarily reflects dynamic heating conditions rather than long-term isothermal stability at a constant temperature. Under steady-state conditions at lower temperatures, gradual oxidation of Mo_2_TiC_2_ could still occur over extended time scales; therefore, the thermal stability inferred from TGA should be interpreted cautiously with this limitation in mind. Overall, it can be concluded that, in principle, MXenes are not ideal materials for oxidation catalysis. Nevertheless, as MXenes continue to be explored for catalytic applications, it will be crucial to precisely delineate their oxidative stability to ensure the rational design and deployment of these materials.

**Figure 5:**
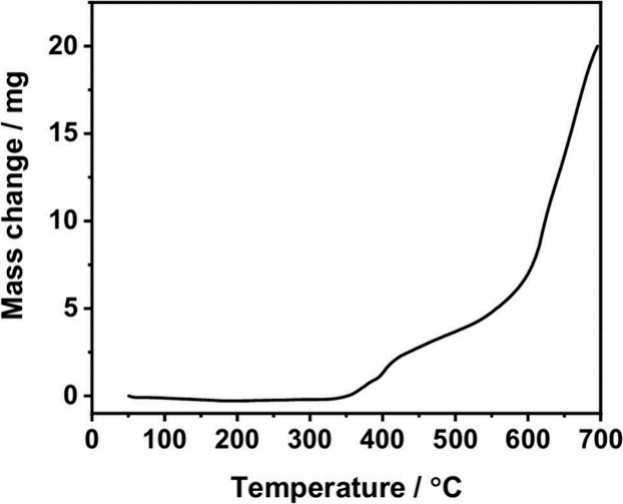
Weight increase undergone by Mo_2_TiC_2_ upon heating in air as a function of the temperature. Reproduced with permission from ref. 45 under Creative Commons Attribution 3.0 Unported License. Copyright 2021 Royal Society of Chemistry.

The preceding discussion has primarily focused on the intrinsic catalytic activity of MXenes, arising from the presence of active sites within their structure. However, as is often the case with other 2D nanomaterials, such as graphene-like carbons, the most common current use of MXenes in catalysis is as support for metal and metal oxide species. These two distinct catalytic roles of MXenes are illustrated in Figure 6.

**Figure 6:**
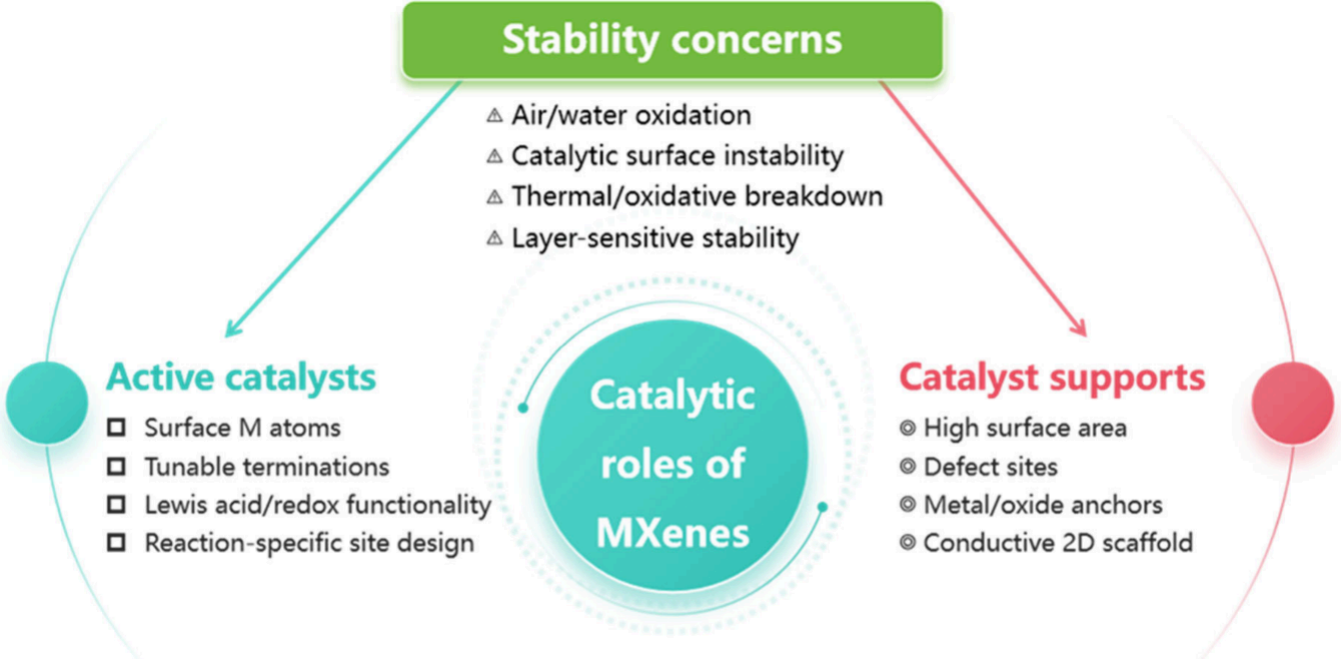
Simplified overview of the two possible roles of MXenes in heterogeneous catalysis and stability concern.

Thus, the 2D morphology of MXenes with large available surface area makes them particularly well suited to support NPs and clusters.^46^ In some cases, strong metal–support interactions (MSIs) are established between the MXene surface and the supported metal species.^47^ This interaction can manifest in various ways, most notably in the formation of metal overlayers on the MXene surface, indicating that the MSI is stronger than the cohesive interaction among the metal atoms themselves.^48^ This phenomenon, often described as “wetting” of the support by the metal, results in thin NPs with large interfacial contact area with the support. Furthermore, as discussed in later sections, the deposition process may even lead to the formation of intermetallic compounds at the interface between the supported metal and the M element of the MXene, reflecting a strong chemical affinity.^49^ Such interactions often contribute positively to catalytic performance by enhancing the stability of anchored metal NPs or clusters.^50^

In relation to the use of MXenes as supports, it is particularly noteworthy that they can serve as host matrices for single-atom catalysts (SACs).^51^ Due to the harsh etching conditions typically employed during synthesis, MXenes inevitably contain M-site vacancies, even when their overall crystallinity is considerably high. These vacancies act as anchoring sites, or “nests”, that can accommodate single atoms (SAs) with high stability. In contrast to many other supports for SACs, where the interaction between the matrix and the isolated metal atom is relatively weak (often due to the atom not being truly incorporated into the framework), MXenes enable genuine incorporation. The single atom fills a vacancy created during the etching process, effectively healing the defect and forming a stable, well-integrated active site.

In sum, integrating MXenes with tunable surface chemistry and the capacity for heteroatom doping or hybridization into heterogeneous catalysis represents a compelling convergence of nanotechnology, materials science, and green chemistry. As the global demand for sustainable technologies intensifies, MXenes stand out as a transformative class of materials, offering the versatility and performance necessary to address long-standing challenges in catalysis and enable practical, real-world applications.

### 1.2 Scope and Structure of This Review

This review focuses on the use of MXenes as heterogeneous catalysts in reactions where heat is required to overcome the energy barrier from reagents to products. Heat, typically obtained from the combustion of fossil fuels, remains the most common energy input in heterogeneous catalysis. With the growing use of alternative, decarbonized energy sources, conventional catalysis is increasingly denoted as “thermal” catalysis to distinguish it from greener alternatives such as electrocatalysis or photocatalysis. Besides thermal catalysis, this review also covers reactions in which light, frequently including IR radiation, serves as the source of heat, or in which thermal energy plays a significant role in light-assisted processes. These reactions are generally described as photothermal or “light-assisted”, and, in many cases, proceed through mechanisms like those of purely thermal reactions, with photoenergy converted into heat.

This review explicitly excludes the use of MXenes in electrocatalysis and photocatalysis, as these two areas have already been extensively reviewed.^21,52–55^ Instead, our intention is to highlight the opportunities that MXenes offer in conventional thermal catalysis, as well as the analogies and differences with other catalysts based on early transition metals. While thermal catalysis will likely need to adapt in the near future, it is clear that heat can also be generated from electricity, for example via Joule heating, microwave irradiation, or solar thermal devices, ensuring the continued relevance of thermal processes beyond the current energy transition.

There exist in the literature a significant number of reviews dealing with the synthesis, properties and applications of MXenes.^55–57^ Several of them include a section summarizing the use of MXenes as heterogeneous catalysts.^58,59^ There are also some reviews that specifically focus on the use of MXenes as solid catalysts that correspond to a topic of the present manuscript.^60–62^ However, the existing reviews have not paid attention to the description of the nature of the active sites on MXenes and how some types of these active sites are spontaneously generated during the harsh conditions needed in the synthesis of MXenes. Since the exact details of the synthetic procedures can vary from one study to others in terms of concentrations, time, temperature, and so on, this raises the issue of reproducibility and confidence on the catalytic data, a problem that is general in heterogeneous catalysis. In this way, the existing literature has not indicated techniques to measure acidity-basicity or reducibility-oxidation, techniques that are widely used to characterize catalysts, but have still not been sufficiently applied to MXene characterization that is more focused on structural information. The need to implement best practices when using MXenes as catalysts has also not been sufficiently encouraged as a necessity to favor the fast and reliable progress of the field and to properly rank the activity of MXenes with valid metrics. Therefore, in writing this review, besides establishing analogies with other metal-based catalysts, emphasis has been made on describing the nature of active sites on MXenes and identifying current knowledge gaps in suitable procedures to increase their density and tune their properties. In that way, the following section summarizes the types of active sites that have been reported on MXenes and proposes suitable characterization techniques for their detection and quantification. Given that MXene-based catalysis is still emerging, some of the comments on active site structures are intended as hypotheses to guide future efforts aimed at enhancing their activity. Best practices for the use of MXenes as catalysts are also discussed, including the importance of providing turnover numbers (TONs) and turnover frequencies (TOFs) to enable meaningful comparison with benchmark materials, and for demonstrating the stability of active sites. In the case of photothermal processes driven by natural or simulated sunlight, solar-to-chemical energy conversion efficiencies should be reported to provide a quantitative assessment of process performance.

The catalytic reactions in which MXenes have been applied as thermal and photothermal catalysts are summarized in Sections 5 and 6. It will be shown that most studies to date have employed MXenes primarily as a support and mostly using Ti_3_C_2_. The unique properties of MXenes in this role will be highlighted, including their potential as hosts for SAs, their ability to support metallene phases with lattice matching to the MXene substrate, and their tendency to form intermetallic compounds with distinct catalytic behavior. These sections will give special attention to the correlation between MXene structure and catalytic efficacy, showing that the intrinsic active sites associated with the structure of MXenes remain largely underexplored and are mainly limited to just a few compositions. A rapid expansion in the library of MXenes used specifically for catalysis is anticipated in the near future. The final section summarizes the key concepts of the review and provides our perspective on future directions and remaining challenges in the field.

## 2 Synthesis and Structural Tuning of MXenes

### 2.1 Brief Overview of Typical Synthetic Routes

MXenes are mostly produced by the top-down selective etching of the “A” element from layered MAX phase precursors, which follow the general formula M_*n*+1_AX_*n*_, in which A is typically Al, but can also be Si, Ga or Zn.^63^ These MAX phases are generally synthesized via metallurgical routes at temperatures of about 1500 °C, using powdered mixtures of M and A metals along with graphite as the carbon source in stoichiometric proportions. The pioneering method, reported by Naguib and co-workers in 2011, involved immersing Ti_3_AlC_2_ powders in 50 wt % aqueous HF to selectively remove Al as soluble AlF_4_^–^ and liberate few-layer Ti_3_C_2_T_*x*_ flakes (Figure 7).^15^ Although highly effective, concentrated HF poses safety hazards and yields predominantly −F/–OH surface terminations, which can limit catalytic performance. Safer protocols were later developed, in which *in situ* HF is generated by reacting LiF or other fluorinated salts with HCl. This approach not only enables Al etching but also facilitates Li^+^ intercalation into the negatively charged Ti_3_C_2_ layers, expanding the interlayer spacing and allowing for exfoliation by mild sonication to yield colloidal suspensions of single- or few-layer MXene.^64^ Other fluoride-containing salts (e.g., NH_4_HF_2_, NaF, KF) provide similar control over MXene etching and exfoliation. Alternatively, quaternary ammonium hydroxides can be used either for etching Al from the MAX phase or as exfoliating agents to obtain single- or few-layer MXene, though these typically result in OH-rich surfaces.

**Figure 7:**
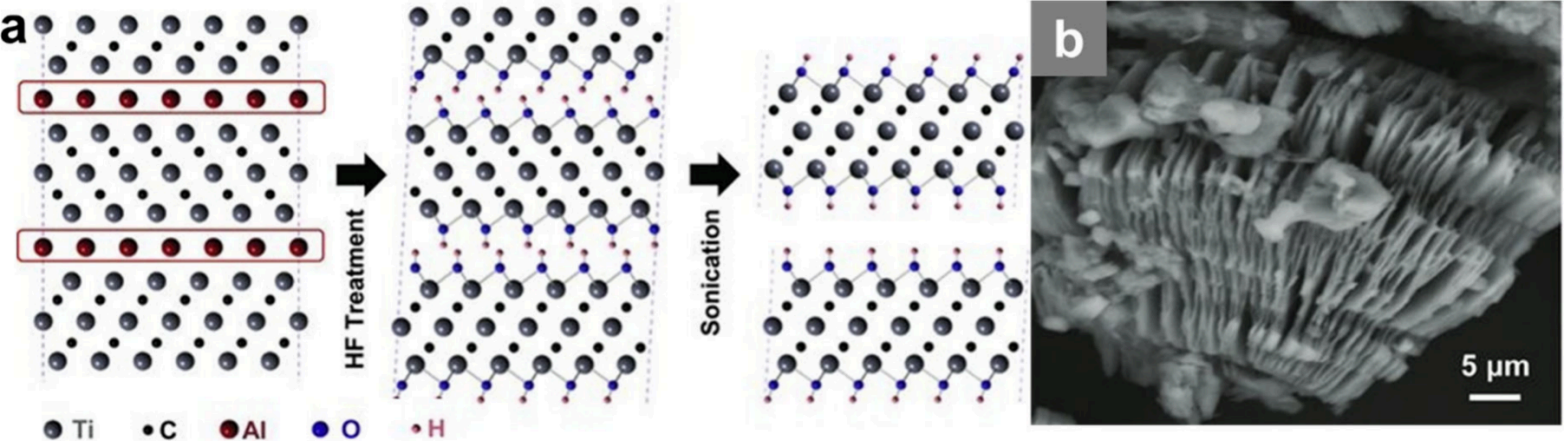
(a) Schematic of the exfoliation process for Ti_3_AlC_2_. (b) SEM image of Ti_3_AlC_2_ after HF treatment. Reproduced with permission from ref. 15. Copyright 2011 Wiley.

To reduce the environmental impact of MXene synthesis, recent efforts have focused on avoiding fluorinated reagents altogether. Electrochemical etching in dilute HCl or organic ammonium salts proceeds via anodic dissolution of the A-layer, yielding Cl-terminated or OH-rich surfaces and enabling gram-scale production under ambient conditions.^65^ Another promising approach is the molten-salt method. This process uses a eutectic mixture of alkali metal chlorides (typically in a 10:1 salt-to-MAX mass ratio) combined with a Lewis acidic transition metal salt as the etching agent.^42^ A common composition includes a 1:1 LiCl-KCl mixture with a stoichiometric amount of ZnCl_2_ or CuCl_2_ to oxidize Al to volatile AlCl_3_. The solid mixture is homogenized and then heated above the melting point of the halides (typically above 450 °C). This treatment results in the oxidation and removal of Al as AlCl_3_ and the simultaneous reduction of the Zn^2+^ or Cu^2+^ to metallic NPs. Subsequent acid washing removes excess metals and salts, producing Cl-terminated MXenes with high conductivity and enhanced oxidative stability.^66–68^ Fluoride-free molten media, such as LiBr/KBr mixtures or NaOH/KOH eutectics containing Lewis acids, have also been used to synthesize MXenes with −Br or −O/–OH terminated surfaces. The mechanism of the molten salt etching may imply an intermediate material in which Al of the MAX precursor is substituted by the Lewis acid metal, the process evolving through an intermediate MAX phase in which “A” corresponds to the etchant metal (Figure 8).

**Figure 8:**
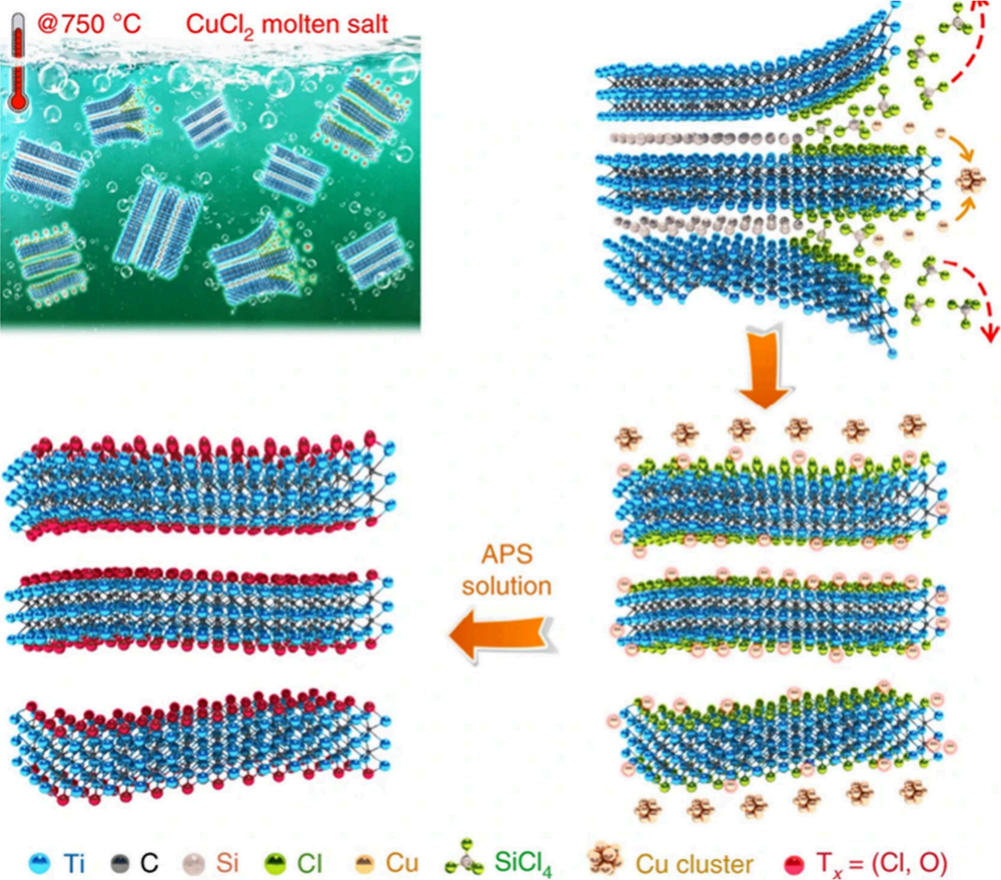
A general Lewis acid etching route for preparing MXenes with enhanced electrochemical performance in nonaqueous electrolyte. Reproduced with permission from ref. 68. Copyright 2020 Nature Portfolio.

While still in early stages, bottom-up synthesis of MXenes has been demonstrated via chemical vapor deposition of Mo_2_CT_*x*_ on Cu foils,^69^ wet-chemical synthesis of Ti_3_C_2_ quantum sheets,^70^ pyrolysis of suitable molecular precursors,^71^ and chemical-free laser ablation of MAX phases.^72^ However, the amount of material produced via these bottom-up routes is generally low, and significant development is still needed to make them scalable.

Thanks to advances in synthesis, multigram-scale production of MXene is now achievable, along with some degree of control over flake thickness, lateral size, and surface terminations, all of which directly influence catalytic behavior. In addition, several postsynthetic modification methods have been reported for tailoring the carbide/nitride layer and surface terminal groups (Figure 9). For example, partial nitridation can be achieved by treating MXene carbides with NH_3_ at controlled temperatures.^73^ Also, a general protocol for installing new surface terminations starting from Cl-terminated MXenes has been developed.^74^

**Figure 9:**
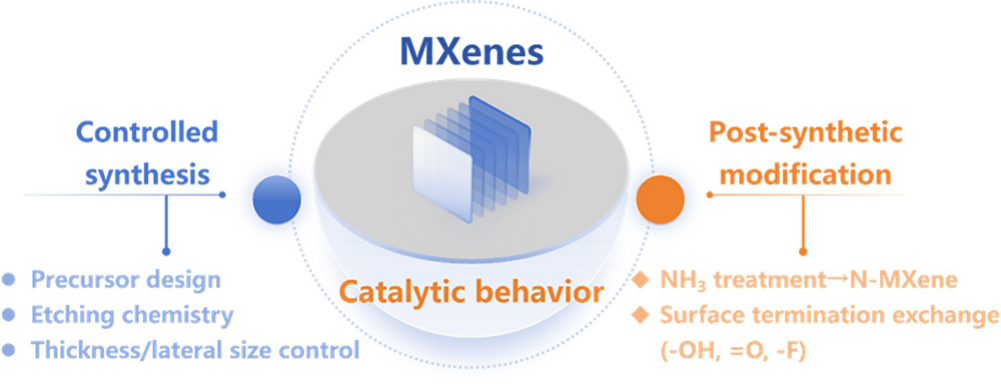
Synthetic and postsynthetic modification strategies for MXenes.

### 2.2 Effects of Synthesis Conditions on Surface Terminations, Vacancies, and Defects

A critical step in MXene synthesis is the selective etching of the “A” element from the MAX phase, typically Al, Ga, or Si, as in Ti_3_AlC_2_. This process usually requires harsh chemical conditions to completely remove the “A” element, thereby exposing the early transition metal “M” to the medium and generating a new surface. The two most widely used etching procedures involve either fluoride-containing reagents in acidic aqueous media at near-ambient temperature or, more recently, molten salt methods, which operate at temperatures above 400 °C using eutectic mixtures of alkali halides and stoichiometric amounts of Lewis acidic metal halides relative to the “A” element. While these etchants are effective in removing “A” layers, they also create highly corrosive environments, often for extended periods, which substantially alter the surface chemistry of the forming MXene particles and may compromise their structural integrity.

In fluoride-based aqueous etching, the resulting surface terminations typically include hydroxyl (−OH), oxygen (−O), and fluorine (−F) groups. These are generally present in an approximate 1:1 atomic ratio of oxygenated to fluorinated species, though the exact composition depends on reagent type, concentration, and other etching parameters. When HCl is added to increase the acidity of the medium, chloride (−Cl) atoms may also be introduced onto the surface. Overall, surface terminations produced by fluoride etching are difficult to control precisely, often resulting in heterogeneous and batch-variable compositions.^75^

In contrast, the molten salt method results in halide-based surface terminations. Due to the strong metal–halide bonds, fluorine and chlorine can be attached to the MXene surface in nearly stoichiometric ratios respect to the M element, leading to well-defined compositions such as Ti_3_C_2_Cl_2_.^66^ In the case of bromide salts, the weaker M–Br bond allows partial substitution by oxygenated groups (−OH and −O) during the aqueous washing step used to remove residual metal bromides. In this regard, surface functionalization, which is unavoidable during “A” element removal, is more precisely defined in molten salt synthesis, particularly for −F and −Cl, compared to liquid-phase etching.

In parallel with surface functionalization, aggressive etching also induces structural defects, particularly metal-site vacancies. As indicated in **eqs. 1 and 2**, a mechanism analogous to “A” removal can apply to the M atoms, leading to the loss of M and resulting in lattice defects such as stacking faults, layer misalignment, or missing layers. Incomplete etching may also leave residual aluminum or byproducts trapped between layers or on the surface, further complicating the physicochemical properties of the resulting MXene. Similarly, molten salt etching can also generate M-site vacancies and, at the same time, enables the incorporation of transition metal cations, such as Fe, Co, Ni, or Cu, from the etching salts into the MXene framework. This substitution creates additional defects where foreign metal atoms replace M atoms, thereby offering a general strategy for fabricating SACs through the stabilization of isolated metal atoms within the MXene lattice (**eq. 3**; Figure 10).

**Figure 10:**
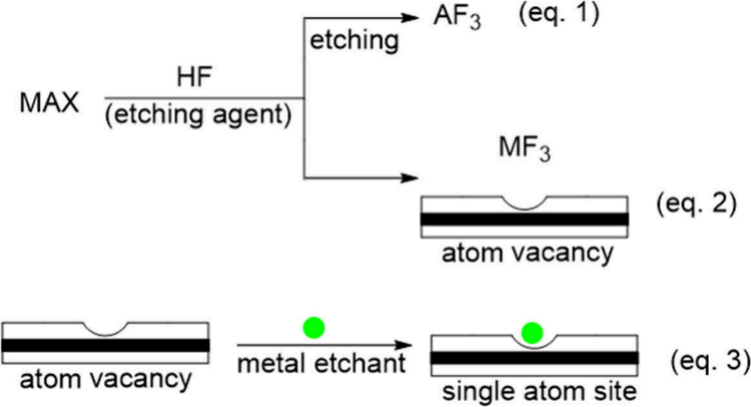
Process of defect generation and single atom installment in MXenes during the etching process.

As such, while “A” removal remains the general route to MXene formation, it inevitably involves a complex interplay of surface modification, defect formation, and active site generation. Precise control over surface chemistry remains a key challenge to be addressed for the rational design of MXene-based catalysts.

### 2.3 Defect and Doping Engineering for Catalytic Site Generation

As discussed in the previous section, etching the “A” element from the MAX precursor inevitably leads to surface functionalization and the generation of atomic vacancies or isolated metal sites. When fluoride-containing reagents in acidic media are used, metal-site vacancies are common due to the absence of compensating metal cations. In contrast, in molten salt etching, such vacancies, if formed, can be healed by Lewis acidic metal cations present in the salt. As just commented, current control over the generation of these defects is minimal and largely limited to variations in reaction conditions. However, in the future, defect engineering can be a means to increase the catalytic activity of MXenes.

Postsynthetic treatments offer additional opportunities to modify surface terminations and, to some extent, control defective structures. For example, Talapin and co-workers reported a general strategy for functional group exchange on Cl- or Br-terminated MXenes.^74^ The method involves treating the MXene with molten bromide salts containing lithium salts of the desired anion. This process is effective for −Cl and −Br due to their relatively weaker metal–halide bonds but is largely ineffective for F- or O-terminated surfaces. Through this approach, a wide variety of surface-modified MXenes, including Ti_3_C_2_Te, Ti_3_C_2_S, and Ti_3_C_2_(NH_2_)_*x*_, as well as termination-free Ti_3_C_2_¨ obtained after LiH reduction, were successfully prepared, demonstrating the generality of the molten-salt-exchange route. One advantage of this approach is the potential for complete replacement of surface halides with new functional groups, as illustrated in Scheme 1 for the case of Br-terminated that is also valid to surface Cl- atoms.

**Scheme 1:**
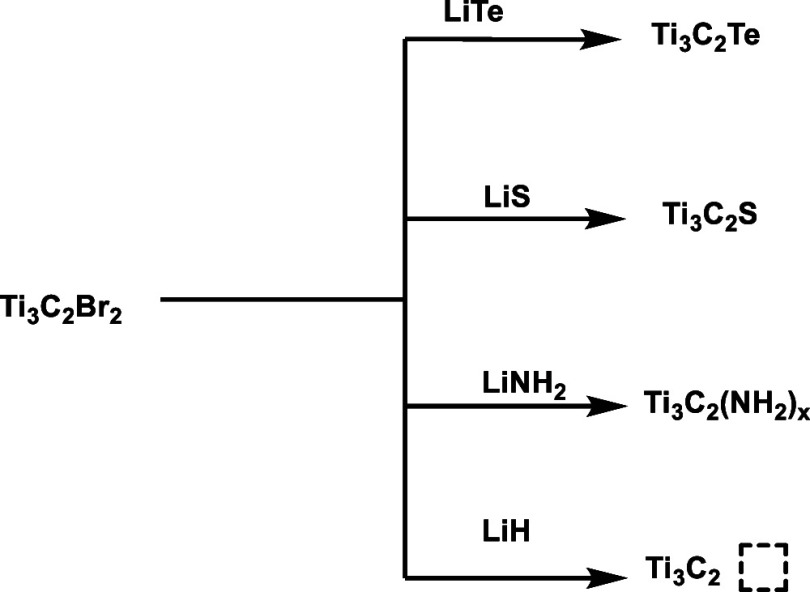
Molten Salt Treatment to Replace Br in Ti_3_C_2_Br_2_ by Other Surface Terminations. The Process is Claimed in Ref 74 to be General for Other MXenes and Also for Cl Atoms. The Dashed Rectangle Indicates a Surface Free Material

Other postsynthetic routes include mild oxidation with aqueous ammonium persulfate (APS), which introduces additional oxygen-containing surface groups. However, excessive APS concentration or prolonged exposure can result in undesirable partial or complete oxidation of MXene into their corresponding metal oxides.^76^ Similarly, treatment with Bu_4_NOH or other hydroxide solutions has been shown to increase surface hydrophilicity, presumably through the introduction of −OH groups.^77^ Thermal treatments under various gas atmospheres also offer routes for surface modification. For instance, heating MXenes under a H_2_ flow can remove surface oxygenated groups,^78^ as indicated by the detection of H_2_O in the effluent gases. Despite their promise, these postsynthetic strategies remain underexplored, especially considering their potential to enhance the density of acidic or redox-active sites in MXenes.

Postsynthetic thermal treatment with NH_3_ represents a common approach to introducing N into MXene structures, leading to the formation of carbonitride phases through partial substitution of C atoms by N. Initially, −NH_2_ groups are likely adsorbed onto the surface before N atoms are incorporated into the carbide lattice, likely beginning at the particle edges. Nitrogen doping is expected to influence the electronic properties of MXenes, including the work function and electron density, thereby potentially improving electron-donating ability and introducing basic sites or local defects. Although detailed mechanistic understanding remains limited, a related study showed that Co­(NO_3_)_2_-assisted hydrogenation of N_2_ to NH_3_ on Co–Mo_2_C involves nitrate-derived nitrogen species that participate in the reaction via a Mars–Van Krevelen-type mechanism.^106^

Looking ahead, defect and doping engineering is anticipated to become a widely used strategy for enhancing MXene catalytic activity. This includes extending dopants beyond transition metals to nonmetals such as P, S, and B, and applying advanced techniques such as plasma treatment and atomic layer deposition. Rational design and controlled implantation of active sites through such methods could yield MXenes as highly tunable catalysts with diverse chemical functionalities.

### 2.4 Tailoring MXene Structure for Thermal/Photothermal Use: Thickness, Conductivity, Morphology Control

Upon etching, the resulting MXene clay is generally constituted by the stacking of multiple MXene sheets due to van der Waals forces and hydrogen bridges. The MXene samples at this stage are denoted as the accordion phase and are characterized by an interlayer distance in the range of 1 nm, as determined by XRD and visible by electron microscopy. The exact interlayer distance depends on the surface terminations, the intercalation of cations that may be present in the medium, and even the etching duration. The surface area of these MXene clays, as measured by isothermal gas adsorption, is generally very small, typically only a few square meters per gram.^79^ However, it has been found that these MXene clays already exhibit catalytic activity, for instance, in the case of Ti_3_C_2_ clay for guanylation of aromatic amines.^80^

Exfoliation of MXene clays into few-layer or single-layer MXenes often requires the use of expanding agents and sonication.^81^ Quaternary ammonium ions and dimethylsulfoxide (DMSO) are widely used to expand the interlayer distance in MXene clays and, in this way, facilitate successful exfoliation.^82^ However, it is likely that these expanding agents, which are difficult to remove from the MXene sample after sonication, could negatively affect catalytic activity by blocking active sites. In fact, ^13^C NMR spectroscopy reveals the presence of a significant proportion of DMSO in the Ti_3_C_2_ sample obtained via DMSO-assisted exfoliation, which remains in the material even after washing.^80^ Thus, the advantage of the larger surface area gained by exfoliation can be offset by the blockage of active sites by strongly bound expanding agents.

The typical lateral size of MXenes is in the micron range. This means that only basal sites can typically be considered in catalysis, due to the small proportion of peripheral atoms. However, it is well established in heterogeneous catalysis that decreasing the average particle size can significantly increase catalytic activity, by increasing the proportion of unsaturated peripheral atoms that act as active sites. In the case of MXenes, sonication of exfoliated samples leads to a decrease in lateral size from microns to below 100 nm. In contrast to other applications, such as film formation, in which larger lateral sizes are preferable, the opposite likely applies in catalysis, where smaller lateral sizes are more suitable and edges can behave as active sites due to incomplete coordination of the exposed elements. However, studies investigating how lateral size affects the catalytic stability of MXenes are still lacking, but they would be valuable in supporting the role of peripheral atoms. The influence of MXene stability as a function of lateral size is also an important factor to be addressed.

The metallic nature and electrical conductivity of MXenes can be important in photothermal applications, in which a light absorber thermalizes the energy of photons. Due to their metallic character with very narrow bandgaps and their black appearance, MXenes have been identified as excellent broadband light absorbers capable of efficiently converting absorbed photons into heat, primarily through ultrafast electron–phonon coupling. Importantly, the reported light-to-heat conversion efficiencies strongly depend on the experimental configuration, spectral range, and definition of efficiency. In particular, for natural solar light to reach the Earth’s surface, it is important to absorb IR light, which represents about 46% of the total solar energy.^83^ Recent experimental and theoretical studies have confirmed the remarkable light-to-heat conversion capability of MXenes, while also emphasizing that the reported efficiencies depend strongly on measurement conditions and sample characteristics. For example, Li et al. reported an internal light-to-heat conversion efficiency approaching unity for Ti_3_C_2_ dispersions under laser irradiation (473–785 nm) in a localized droplet-heating configuration, with an uncertainty of approximately ± 5%, and a solar-driven water-evaporation efficiency of about 84% under one-sun illumination.^84^ It should be emphasized that this value refers to the fraction of absorbed photon energy converted into heat under highly confined conditions, rather than a device-level or solar-to-thermal efficiency under standard illumination. In parallel, Finite-Difference Time-Domain (FDTD) simulations have shown that Ti_3_C_2_T_*x*_-based hybrid absorbers (e.g., Ti_3_C_2_T_*x*_/W architectures) can achieve broadband solar absorptivity exceeding 95% over the 300–2500 nm range when layer thickness and optical constants are optimized.^85^ Taken together, these findings indicate that MXenes are highly efficient broadband absorbers capable of converting a large fraction of incident solar energy into heat, although the absolute efficiency varies with wavelength range, layer thickness, surface terminations, and experimental configuration. To provide a quantitative perspective and avoid overgeneralization, representative light-to-heat efficiencies reported for MXene-based materials are summarized in Table 1 together with the corresponding measurement methods, illumination conditions, and uncertainty sources, enabling a more quantitative and contextualized comparison across studies. Films of MXenes with appropriate vacuum insulation can be used as coatings to design efficient solar ovens that reach temperatures above 250 °C under natural sunlight.^86^ Therefore, for photothermal applications, the narrow bandgap and metallic/thermal conductivity of MXenes are especially advantageous. These coatings can be prepared by depositing black MXene inks on glass surfaces. In addition, the 2D morphology of MXenes makes these materials appropriate to establish junctions and interfacial contact with other materials, resulting in a heterojunction combining the unique MXene properties with those of other components, thereby boosting the efficacy of the resulting composite.

**Table 1:** Reported Solar-to-Thermal (Light-to-Heat) Conversion Efficiencies of Representative MXene-Based Materials

MXene system	Morphology/thickness	Spectral range/illumination condition	Measurement method	Reported efficiency (%)	Ref.
Ti_3_C_2_ dispersions	Few-layer flakes in aqueous droplet (∼nm)	473–785 nm laser irradiation	Localized laser droplet-heating experiment	≈100 (internal efficiency)	^84^
Ti_3_C_2_ dispersions (film on water)	Thin floating film	1 sun (1 kW m^–2^)	Solar water-evaporation test	≈84	^84^
Ti_3_C_2_T_*x*_/W hybrid multilayer	Simulated 2D/3D absorber	300–2500 nm (simulated solar spectrum)	FDTD electromagnetic simulation	95–97 (absorptivity)	^85^
Ti_3_C_2_T_*x*_/microcrystalline cellulose composite aerogel	3D Porous aerogel (6.4 wt.% MXene)	1 sun (1 kW m^–2^)	Solar evaporation (mass loss + IR imaging)	91.3 (1 sun)	^86^
				92.8 (3 sun)	
Vertically aligned rGO/Ti_3_C_2_T_*x*_ hydrogel	3D Porous hydrogel architecture	1 sun (AM 1.5 G, 100 mW cm ^–2^)	Solar steam-generation test (mass-loss + thermal profiling)	≈93.5	^87^
MXene-decorated 3D honeycomb fabric	3D Textile evaporator	1 sun (AM 1.5 G)	Solar steam generation (mass loss + thermal camera)	93.5	^88^
MXene/MnO_2_@luffa sponge (LS) nanocomposite	Hierarchical porous layer	1 sun (1 kW m^–2^)	Solar evaporation test + temperature mapping	85.3	^89^

In summary, the synthesis and structure of MXenes critically determines their physicochemical properties and, consequently, their catalytic performance. Top-down etching methods, whether based on fluoride-containing aqueous solutions or dry molten salt approaches, not only define the nature and distribution of surface terminations but also introduce structural defects, vacancies, offering opportunities for single-atom incorporation. Postsynthetic treatments, including chemical, thermal, and doping strategies, further expand the tunability of MXenes, enabling controlled modulation of active sites. Moreover, morphological parameters such as flake thickness, lateral size, and surface cleanliness significantly impact both thermal and photothermal catalytic behavior. Collectively, this section highlights that rational control over synthesis conditions is essential to unlocking the full potential of MXenes as catalysts.

## 3 Characterization Techniques and Structure–Property Relationships

Understanding the structure–activity relationships of MXenes requires identifying the nature, location, and functionality of their catalytically active sites. This knowledge could eventually lead to more advanced MXene catalysts, especially engineering for increasing the density of the active sites required in the reaction. In this section, the discussion is organized according to the distinct roles that MXenes and their parent MAX phases play in heterogeneous catalysis. Section 3.1 discusses intrinsic active sites within MXenes, including exposed metal centers, vacancies, and surface terminations, together with the catalytic behavior of MAX phases, thereby providing a basis for understanding how etching and structural transformation give rise to active sites in derived MXenes. Section 3.2 focuses on MXenes as supports for external active species such as SAs, NPs, or molecular complexes, emphasizing how their structural and electronic properties govern metal–support interactions and catalytic behavior. Section 3.3 summarizes the advanced characterization tools, ranging from atomic-resolution microscopy and spectroscopy to theoretical simulations, which enable precise identification of active sites and correlation with catalytic performance. Together, these subsections establish a coherent framework linking the evolution of structure from MAX phases to MXenes with their catalytic functions.

### 3.1 Active Sites in MXenes and Their Characterization

Regarding structural active sites, surface functional groups, accessible bare M elements, and vacancies associated with both T and M atoms are first discussed. A section on the role of internal X as sites in Mars-Van Krevelen-type mechanisms is also included. The comparison of the performance of MXenes with the catalytic activity of the MAX phase is also briefly mentioned. The second part of this section refers to the unique structural features that make MXenes special supports for SAs and metallenes.

Insights into the nature of MXene active sites have also been informed by studies on molecular complexes of early transition metals, which serve as homogeneous analogues with comparable coordination environments and redox flexibility. For example, Ti- and Mo-based molecular complexes exhibit similar *d*-orbital configurations and metal–ligand interactions to those present on MXene surfaces, providing useful guidance for understanding adsorption geometries, oxidation-state changes, and intermediate stabilization. Introducing this comparative perspective conceptually bridges homogeneous and heterogeneous catalysis and provides a coherent framework for interpreting MXene reactivity.

#### 3.1.1 Surface Functional Groups as Active Sites

Structural M elements in MXenes bonded to surface terminations can behave as catalytically active sites for certain processes. In most cases reported in the literature, the M–T active sites correspond to the T groups that are installed on the surface during the etching process. Surface chemistry is critical, controlling the physical, adsorptive and chemical properties of MXenes. Terminations alter the work function (3.5–6.2 eV), hydrophilicity, and zeta potential of MXenes. The hydrated interlayer galleries easily undergo up to 2–4 nm of expansion without compromising MXene structure integrity. From the catalytic point of view, certain surface functional groups and their vacancies can behave as acid or basic sites.

Unlike many 2D materials, MXenes are intrinsically hydrophilic; combined with their negative surface charge, this property enables stable dispersions in water and green solvents—an advantage for scalable processing into inks, electrodes, and macroscopic architectures,^90^ but also for catalysis in liquid phase reactions. In the most common procedure using fluorinated etching agents in the aqueous phase, the surface terminations are −O, −OH, and −F.^91^ Particularly, oxygenated groups could behave either as acidic sites (M–OH) or as basic sites (−O−). However, acidity-basicity measurements of these materials indicate a very low density of acid and basic sites—much lower than expected based on the population of oxygenated groups.^92^ This suggests that surface oxygenated groups are essentially neutral or possess only weak strength.

Even if devoid of acidity or basicity, M–T groups may exhibit other types of activity. In one example, Ti_3_C_2_ prepared from Ti_3_AlC_2_ by HF etching was found to catalyze epoxidation of styrene using H_2_O_2_ as the oxidant (Figure 11).^93^ The catalytic process resembles those reported for Ti atoms anchored on silicalite and other porous silicas, in which the mechanism involves the formation of a titano-hydroperoxide intermediate via substitution of the Ti–OH groups.^24^ However, in contrast to Ti-beta and other titanosilicates, Ti_3_C_2_ exhibits poor selectivity in the epoxidation of styrene, suggesting the presence of additional undesired active sites or an alternative reaction mechanism. Thus, it would have been important to detect the ≡TiOOH intermediate and to correlate the physicochemical properties of Ti_3_C_2_ with its catalytic efficacy for this reaction. The Ti_3_C_2_ catalyst also deactivates upon consecutive reuse.^93^ As discussed in the Introduction, MXenes have limited stability under oxidizing conditions, often transforming into their corresponding metal oxides. Therefore, it would have been important to verify whether the observed deactivation results from the instability of Ti_3_C_2_ under the reaction conditions and if TiO_2_ is formed because of carbide layer oxidation.

**Figure 11:**
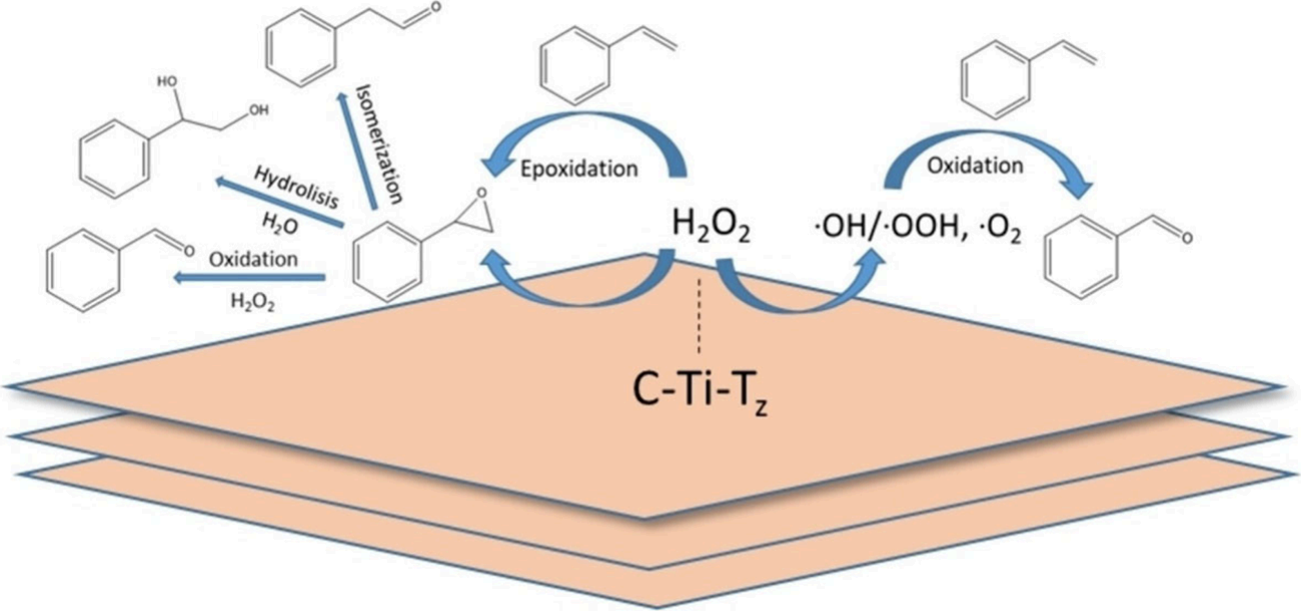
Cartoon illustrating the catalytic activity of Ti_3_C_2_ in styrene epoxidation by H_2_O_2_. Reproduced with permission from ref. 93. Copyright 2024 Wiley.

In another example also involving M–T sites in oxidation reactions using molecular O_2_ as the oxidant, it was found that V_2_C could promote the aerobic oxidation of indane at 120 °C to the corresponding alcohol/ketone mixture.^94^ In this case, modification of the surface terminal groups by thermal treatment under hydrogen (to reduce oxygenated groups) or with APS (to convert −OH into = O) led to a decrease in the catalytic activity observed for V_2_C synthesized by HF etching. Thus, this study represents an attempt to correlate MXene properties with its catalytic efficacy, although the surface modification treatments resulted in lower activity than the material directly obtained from the V_2_AlC etching. Upon reuse, the catalytic activity gradually declined from 41 to 35% indane conversion.^94^ Importantly, TEM characterization showed that although the 2D morphology was maintained, the material became mostly amorphous during the reaction, indicating significant structural changes (Figure 12). Therefore, it appears that the M–OH sites responsible for catalytic activity under oxidative conditions may also participate in the transformation of MXene into the corresponding metal oxide.

**Figure 12:**
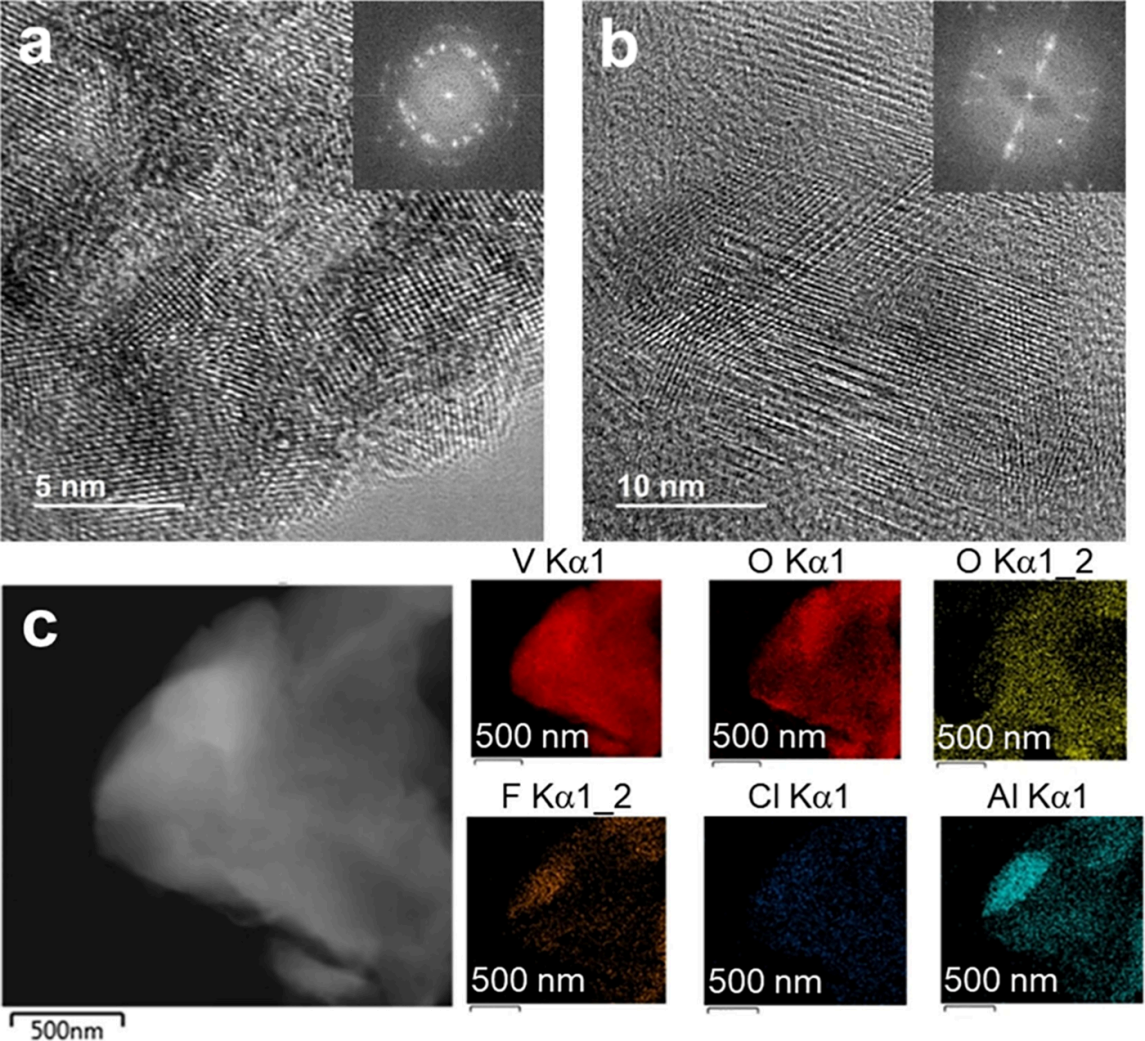
(a, b) High-resolution TEM images of the V_2_C MXene used in the study. The insets correspond to the selected area electron diffraction patterns of the sample showing its crystallinity. (c) TEM and EDS images of the reused V_2_C samples. Reproduced with permission from ref. 94. Copyright 2024 Wiley.

Density functional theory (DFT) calculations suggest that surface hydroxyl (−OH) and oxygen (−O−) groups could also serve as active centers for hydrogenation reactions.^95^ In a pioneering study, it was found that Ti_3_C_2_T_2_ and Ti_3_CNT_2_ convert furfural to hydroxymethylfuran using hydrogen gas or isopropanol as reducing agents.^95^ Ti_3_C_2_T_2_ undergoes deactivation, while Ti_3_CNT_2_ was considerably more stable.^95^ The higher stability of Ti_3_CNT_2_ over Ti_3_C_2_T_2_ was justified based on the X-ray photoelectron spectroscopy (XPS) data shown in Figure 13. There, a significant decrease in the intensity of the Ti 2p peak was observed for Ti_3_C_2_T_2_ but not for Ti_3_CNT_2_. Accordingly, it was proposed that this deactivation is due to the strong adsorption of organic compounds on the surface of Ti_3_C_2_T_2_ and the occurrence of a small percentage of Ti leaching, as determined by analysis of the liquid phase. In comparison with Ti_3_C_2_T_2_, Ti_3_CNT_2_ does not exhibit this Ti 2p peak intensity decrease, implying weaker adsorption of organic species on its surface. Accordingly, it seems that product or byproduct adsorption energies control the performance and stability of the MXene for this hydrogenation reaction.

**Figure 13:**
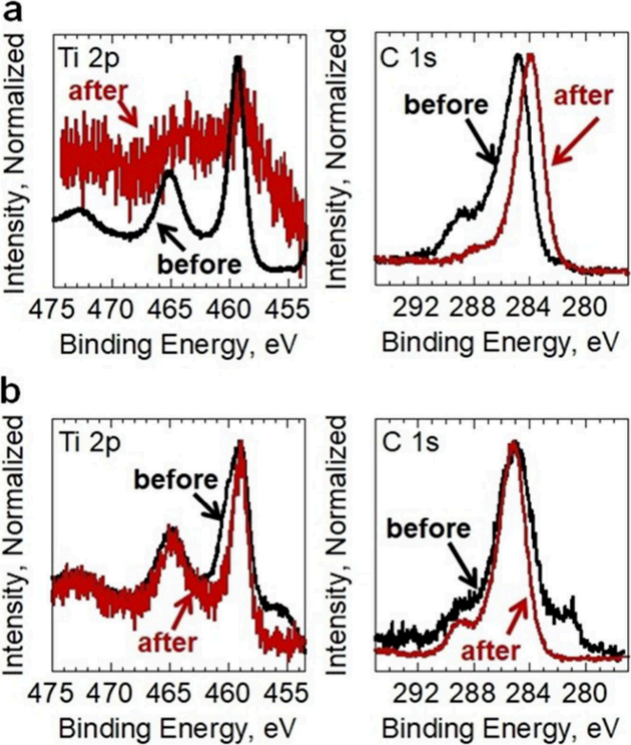
XPS core levels of Ti 2p and C 1s for (a) Ti_3_C_2_T_2_ and (b) Ti_3_CNT_2_ before and after reaction, showing the notable loss of intensity of the Ti 2p peak for Ti_3_C_2_T_2_. Reproduced with permission from ref. 95. Copyright 2020 Wiley.

Similarly, periodic DFT calculations on a series of 2D MXenes based on carbides and nitrides indicate that interaction of molecular hydrogen with their surface should lead to dissociation of molecular hydrogen with an almost negligible barrier.^96^ In a certain way, the surface becomes functionalized with metal hydride groups even at low temperatures. Furthermore, these calculations show that Fe_2_C, W_2_N, and Mo_2_C may behave as promising catalysts for hydrogenation reactions. This work provides theoretical evidence that these three MXenes can behave as potential solid catalysts for hydrogenation reactions. Experimental validation of these calculations will show the importance of DFT studies in leading the design of the most active MXene catalysts.

Overall, surface terminations in MXenes, particularly oxygenated functional groups such as −OH and −O–, play an important yet complex role in catalysis. While their intrinsic acidity or basicity appears weak based on experimental measurements, these groups can still participate in redox reactions and influence catalytic behavior. The catalytic activity of M–T sites, however, is often accompanied by structural instability under oxidative conditions, highlighting the need for careful stability assessment. Further experimental studies are required to validate theoretical predictions and to disentangle the contributions of surface terminations from other potential active centers.

#### 3.1.2 Defect Sites as Active Sites

##### 3.1.2.1 Atom Vacancy-Induced Defect Sites

Atom vacancies can correspond to any of the elements present in MXenes, including M, X, and the surface terminal groups T. In the most common etching treatments of the MAX precursors to prepare MXenes, removal of the “A” element is the main process taking place, forming, for instance, AF_3_ that evolves as a gas or generates water-soluble AF_4_^–^ anions. However, although in much lower proportion than AF_3_, similar fluorinated products of the “M” element, giving rise to analogous MF_3_ or MF_4_^–^ species, can also occur to some minor extent. Additionally, X atoms and T groups can also be removed during etching. These unwanted processes generate atom vacancies and defects in the resulting MXene sheet.

##### 3.1.2.2 M Vacancy-Induced Defect Sites

Metal vacancies in MXenes are generally considered active centers where substrates and reagents can be adsorbed due to the under-coordination of neighboring M atoms in the structure. In addition, these sites can anchor atoms of other metal elements to form SAC centers, thereby promoting improved dispersion, or they can serve as interaction points where MXene sheets interact with clusters or NPs, enhancing their stability and catalytic efficiency. Moreover, beyond local effects, metal vacancies alter the electronic properties of MXenes, influencing global features such as work function and the material’s ability to donate or accept electron density from supported active metal species. This can enhance charge transfer during catalytic processes, leading to improved reaction rates.

The unique arrangement, unsaturated coordination, and electron density of atoms around vacancies can facilitate specific catalytic mechanisms. By controlling the concentration and distribution of vacancies through the selection of etchant concentration, temperature, and etching duration, it seems possible to tailor, to some extent, the properties of MXenes and optimize their performance for specific catalytic applications. Furthermore, due to their 2D morphology, these defects are readily accessible to reactants and substrates.

When Ti_3_C_2_ MXene is derived from the parent Ti_3_AlC_2_ precursor by etching Al with HF acid and subsequent exfoliation, controlled treatment with H_2_O_2_ at varying concentrations promotes its partial oxidation, transforming it into titanium oxide (TiO_*x*_) nanoclusters (NCs) anchored on a carbon-rich, silk-like substrate.^97^ The etching and exfoliation processes generate highly reactive Ti vacancies within the MXene structure, which apparently serve as ideal nucleation sites for TiO_*x*_ NCs under oxidative environments.

Electron paramagnetic resonance (EPR) spectroscopy showed a clear signal at *g* = 1.946 for the exfoliated Ti_3_C_2_ MXene flakes. Observation of this signal provides evidence for the presence of single Ti vacancies or vacancy clusters formed during the etching procedure. The EPR signal is indicative of the existence of Ti^3+^ defects.^97^ HF etching of nascent MXene could lead to breakage of Ti–Al bonds, resulting in the leaching of TiF_*x*_^4–*x*^ species and the formation of Ti vacancies on the MXene sheet. In that way, EPR spectroscopy can be used as a qualitative method to demonstrate the presence of Ti atom vacancies. Although less studied, EPR spectroscopy can also be applied to identify vacancies in MXenes other than Ti_3_C_2_, such as those containing V or Cr, which also involve paramagnetic oxidation states.

Since these defects are highly oxyphilic, they are expected to form oxides, particularly upon contact with chemical compounds such as H_2_O_2_. According to DFT calculations, surface Ti vacancies in exfoliated Ti_3_C_2_ act as key reactive sites for the formation of TiO_*x*_ clusters through oxidation. The presence of surface Ti vacancies causes distortions to neighboring atoms and leads to clear electronic delocalization.^98^ While Ti_3_C_2_ prepared by 40% HF etching displays an EPR signal at *g* = 1.946, indicating the presence of single atom vacancies or vacancy clusters, after H_2_O_2_ oxidation, the resulting Ti_3_C_2_ sample displays a strong signal at *g* = 2.009 (Figure 14), indicating that the surface Ti vacancies exist in a new coordination environment. Accordingly, it can be expected that vacancies enhance the natural tendency of MXenes to undergo oxidation, and they should lead to a decrease in catalytic stability under oxidative conditions.

**Figure 14:**
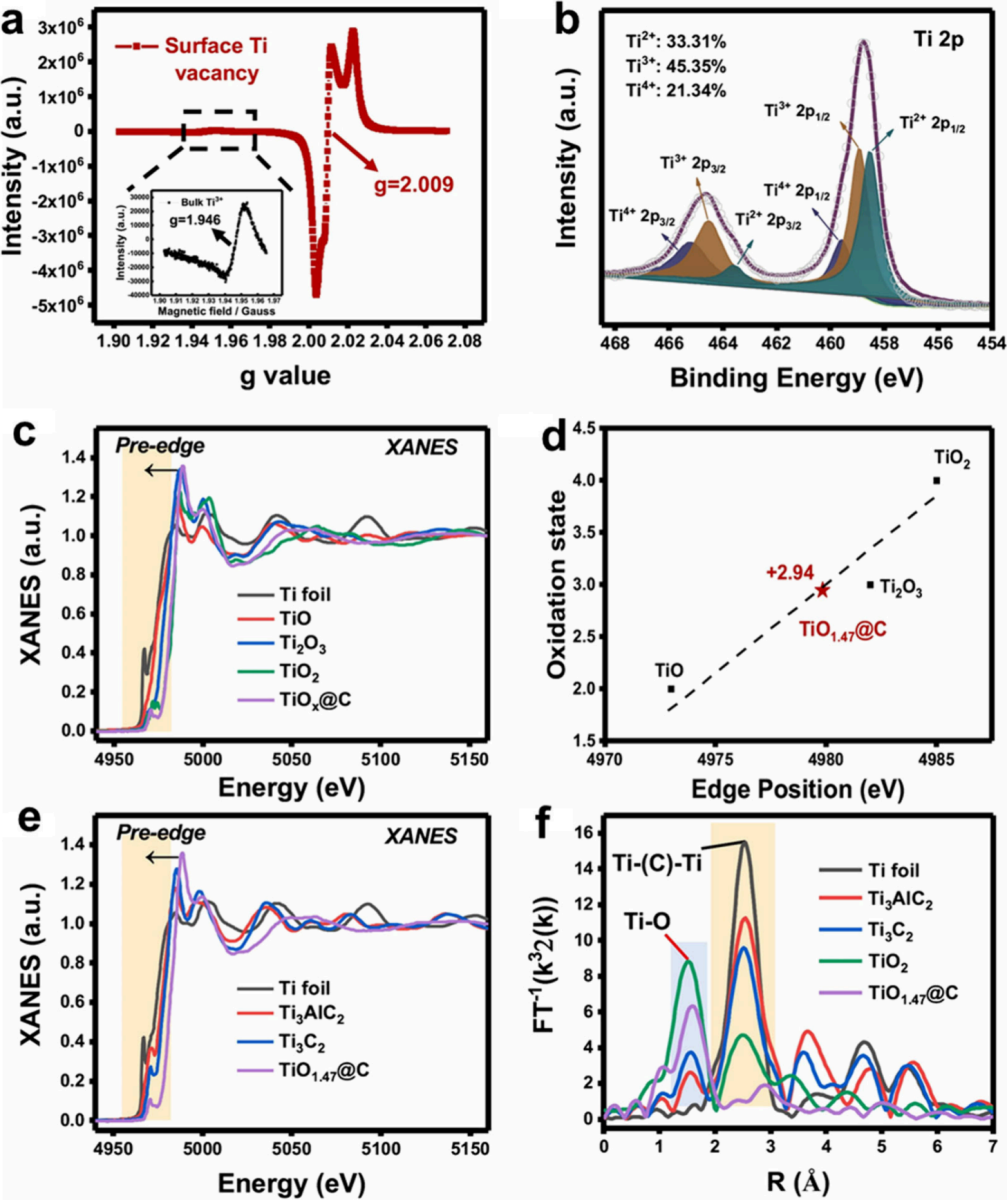
Chemical state and atomic local structure of TiO_*x*_@C catalyst formed from Ti_3_C_2_ during the Fenton reaction. (a) EPR spectra (77 K) of TiO_*x*_@C showing a clear surface Ti vacancy signal (*g* = 2.009) and a significantly weaker bulk Ti^3+^ signal (*g* = 1.946, Inset). (b) High-resolution XPS spectrum of Ti 2p. The percentage of valences of Ti element was calculated as Ti^2+^: 33.31%, Ti^3+^: 45.35%, and Ti^4+^: 21.34%. (c) Normalized Ti K-edge XANES spectra of Ti foil, TiO, Ti_2_O_3_, TiO_2_, and TiO_*x*_@C. (d) Estimation of the titanium oxidation state in TiO_*x*_@C. According to the XANES spectra of Ti from the edge position of references to TiO, Ti_2_O_3_, and TiO_2_, Ti was calculated to be in an average of 2.94^+^ oxidation state in TiO_*x*_@C, with *x* = 1.47. (e) Normalized Ti K-edge XANES spectra of Ti foil, Ti_3_AlC_2_, Ti_3_C_2_, and TiO_1.47_@C, respectively. (f) The *k* ^3^-weighted FT spectra from Ti K-edge extended X-ray absorption fine structure (EXAFS). Reproduced with permission from ref. 97. Copyright 2023 National Academy of Sciences.

The presence of multiple oxidation states for Ti in Ti_3_C_2_ MXene is revealed by X-ray Absorption Near Edge Spectroscopy (XANES) at the Ti K-edge (Figure 14), which shows contributions from Ti^0^, Ti^2+^, Ti^3+^, and Ti^4+^. This multivalence is key to enabling the Fenton-like catalytic activity of Ti_3_C_2_, making possible single-electron transfer with H_2_O_2_.^97^ From these XANES measurements, an average Ti oxidation state of +2.94 was estimated, indicating a blending of valences that appears to be critical for the performance of Ti_3_C_2_ as a Fenton-like catalyst.^97^ Accordingly, it can be predicted that MXene activity as Fenton catalyst will increase along the proportion of low Ti oxidation states, allowing to correlate the structure of MXene with its activity. In Fenton catalysis, ·OH radicals are generated via one-electron transfer from the catalyst to H_2_O_2_, resulting in the cleavage of the O–O bond. The catalytic cycle is completed when the site receives an electron from another H_2_O_2_ molecule acting as a reducing agent and becomes oxidized to O_2_. The cycle is shown in Scheme 2, in which TiO_*x*_@C denotes the material derived from Ti_3_C_2_ during the catalytic cycle in the presence of H_2_O_2_. To act as a catalyst for this redox cycle corresponding to H_2_O_2_ disproportionation, the work function of the MXene derived material should play a key role, since it must be between the E^0^ potential of H_2_O_2_ being oxidized to O_2_ and being reduced to H_2_O. However, it is important to emphasize that the catalytic stability of TiO_*x*_@C under oxidative conditions must be carefully evaluated to confirm its stability for this reaction.

**Scheme 2:**
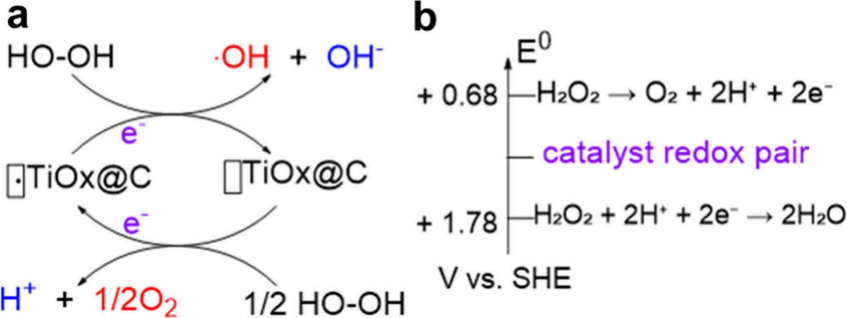
(a) Proposed Mechanism of the Catalytic Turnover Promoted by Unpaired Electrons Observed by EPR in TiO_*x*_@C Having Ti Vacancies.^a^ (b) Standard Redox Potential of the H_2_O_2_ Redox Pairs Involved in the Fenton, Indicating That Catalyst Sites Should Have an Intermediate Redox Potential

a H_2_O_2_ undergoes dismutation becoming reduced to ^·^OH radicals and oxidized to O_2_, the cycle being triggered by electron shuttle from and to TiO_*x*_@C for reduction and oxidation, respectively.

##### 3.1.2.3 Surface Termination Vacancy-Induced Defect Sites

T groups on the surface can also be missing, leaving accessible M atoms that can interact with substrates and reagents. In comparison to molecular organometallic complexes, DFT calculations indicate that a single bare M atom is generally not sufficient to promote a catalytic cycle due to the rigid configuration of the M atom within the MXene structure that does not allow the coordination changes occurring in the mechanism of molecular organometallic analogues. Thus, calculations in MXene models indicate that at least two or more of these T-free M atoms are frequently required to cooperate in the catalytic cycle. This is because M atoms are typically able to absorb only a single reagent or substrate molecule due to structural constraints. Neighboring M atoms then allow bond formation between closely located reagents and substrates bonded to different M atoms. DFT calculations support that these M atoms lacking surface terminations can act as active sites in oxidative aniline coupling promoted by Nb_2_C,^92^ hydroamination of C≡C triple bonds,^99^ and in guanylation of carbodiimides^80^ (Scheme 3). An example of this cooperative mechanism is provided in Figure 15.^80^ This contrasts with analogous mechanisms in molecular complexes, in which a single M atom can coordinate with multiple reactive molecules. However, frequently the reaction intermediates in molecular complexes have similar structures to those calculated as optimal in MXene models. As commented earlier at the beginning of Section 3.1, the structure and performance of organometallic complexes can provide certain guidelines in the design of active sites in MXenes and this is apparently the case for this reaction.

**Scheme 3:**
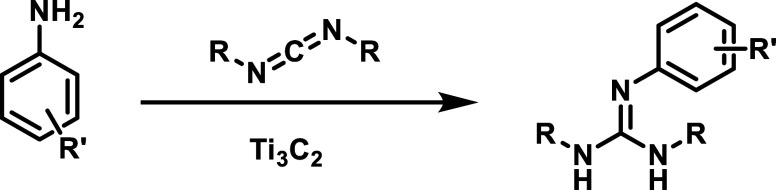
Guanylation Reaction of Carbodiimides by Anilines Catalyzed by Ti_3_C_2_ ^80^

**Figure 15:**
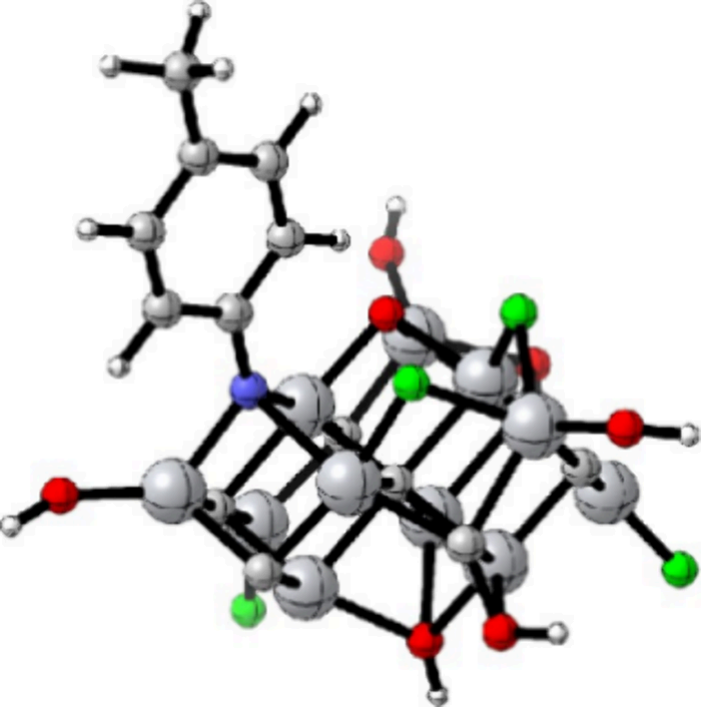
35-Atoms model of the Ti_2_C structure in which toluidine becomes deprotonated by releasing two H^+^ to the −O– surface and becomes bonded to three Ti neighbor atoms lacking surface functional groups. Thus, the model indicates that activation of the amino group cannot occur at a single Ti atom. Color code: Blue = N, Red = O, White = H, Green = F, Gray (small) = C, Gray (big) = Ti. Reproduced with permission from ref. 80. Copyright 2025 Elsevier.

Characterization of surface groups by high-resolution TEM indicates that T vacancies tend to appear as patches in the structure.^91^ This could reflect the greater thermodynamic stability of grouped T defects in comparison to the same number of isolated T defects, lending further credibility to calculations involving cooperative activity among adjacent M atoms.

Surface terminations in MXenes exhibit characteristic vibrational modes in the low-frequency region of Raman spectra.^101^ Changes in this spectral region have been taken as experimental evidence for the participation of these groups in the reaction mechanism. In one example using Ti_3_C_2_ as a hydroamination catalyst, it was observed that aniline adsorption generated surface OH groups, in agreement with a proposed mechanism in which a proton from aniline coordinated to Ti is transferred to an −O– group acting as a basic site (Figure 16).^99^ These variations in Raman spectroscopy offer an opportunity for *in situ* studies of reaction mechanisms.

**Figure 16:**
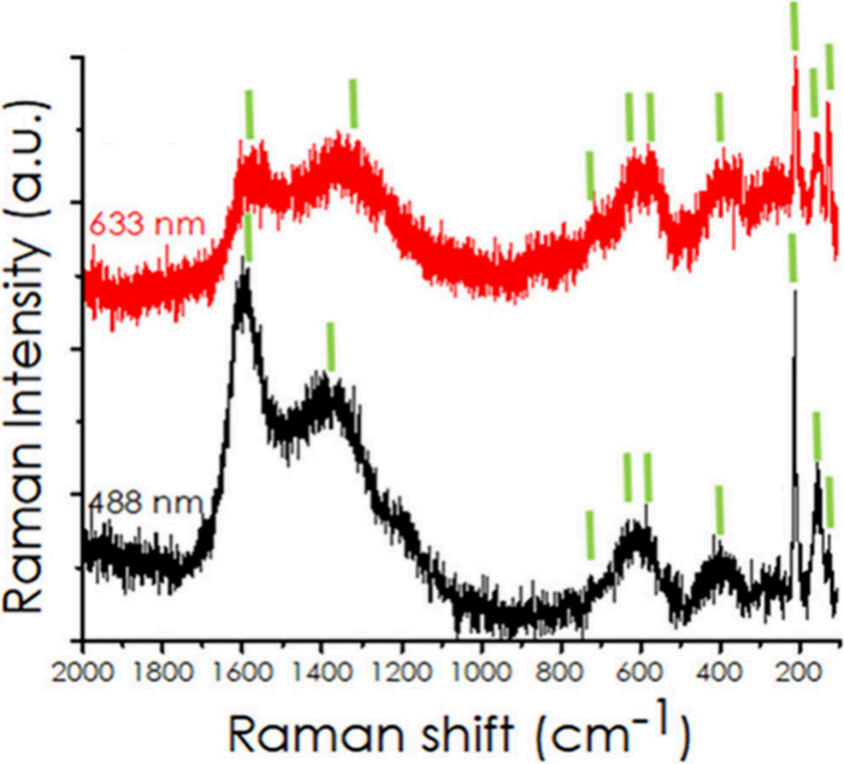
Raman spectra of the Ti_3_C_2_ catalyst recorded at excitation wavelengths of 488 nm (black line) and 633 nm (red line). The green lines correspond from left to right the wavenumber values of 1599, 1389, 720, 622/621, 584, 380, 213, 154, and 128 cm^–1^, respectively. These Raman shifts have been proposed that can be used to follow the reaction mechanism. Reproduced with permission from ref. 99. Copyright 2025 American Chemical Society.

It has been reported that reductive treatments, such as heating MXene at moderate or high temperatures, particularly under a H_2_ or reducing atmospheres, can remove some surface terminations, including some oxygenated groups.^102^ One study has provided detailed information on the surface modification of Nb_2_CT_*x*_ obtained by HF etching from Nb_2_AlC.^47^ In this example, XANES analysis compared Nb_2_CT_*x*_ data with those of the corresponding metal, metal oxides, and metal carbide to examine the degree of oxidation produced during chemical etching in aqueous conditions. While the shape of the spectra might not differ significantly, the edge energy provides strong evidence of the differences among carbides, oxides, and MXenes decorated with various terminations, such as −OH, −O, and −F. It was found that Nb_2_CT_*x*_ prepared using 50 wt % HF as the etchant exhibits a slightly higher edge energy than that of NbC, despite showing a similar XANES spectral shape.^47^ This difference in edge energy is mostly attributed to the partial oxidation induced by the presence of surface functional groups or Nb_2_O_5_ on the surface, which are almost absent in the case of NbC.^47^

Negligible changes and no shifts were observed in the M-C peak in XPS when reducing MXenes (Nb_2_CT_*x*_) under 3% H_2_ diluted in He at 350 and 550 °C, indicating that the structure is preserved under high temperatures and highly reductive atmospheres.^47^ Upon exposure of the reduced sample to air, the intensity of the M-C peak in the XPS data decreases, while the one corresponding to the oxide becomes better resolved, suggesting that oxide species enrich the surface.^47^ Reduction at 350 °C desorbs adsorbed oxygen species and most surface terminal −OH/–O groups present on the MXene surface.^47^ Hydrogen temperature-programmed reduction (H_2_-TPR) is typically used to measure the reducibility of oxygen species on a catalyst, indicating the oxygen storage capacity of the material and providing information on its reducibility by H_2_. Following H_2_-TPR of Nb_2_CT_*x*_, a peak indicative of H_2_O at 340 °C appears, which is attributed to the removal of −OH and −O functional groups from the Nb_2_CT_*x*_ surface.^47^ −F terminations can only be fully removed upon exposure to H_2_ atmosphere at 550 °C, implying that −F binds more strongly to Nb compared to other surface terminations. Upon removal of surface functional groups, the exposed MXene M metal becomes coordinatively unsaturated and exists in lower oxidation states, making it more prone to immediate oxidation in air.^47^

This provides a general, yet still underexplored, strategy to generate T vacancies in a controlled manner. If confirmed, surface modification via pretreatment to induce T defects, followed by catalytic application, could serve as a general methodology for preparing MXenes with enhanced catalytic activity. In this way, MXene preparation conditions will correlate with catalytic efficiency for those reactions involving T vacancies as active sites. In fact, DFT calculations predict that surface-free MXenes should exhibit reactivity toward CO_2_ to generate CO^103^ or be able to dissociate N_2_,^104^ both of which are reactions with high energy barriers that often constitute the rate-limiting step in catalytic mechanisms involving these molecules. Figure 17 illustrates some of these theoretical predictions, highlighting the potential of surface modification as a route to develop highly active MXene-based catalysts.

**Figure 17:**
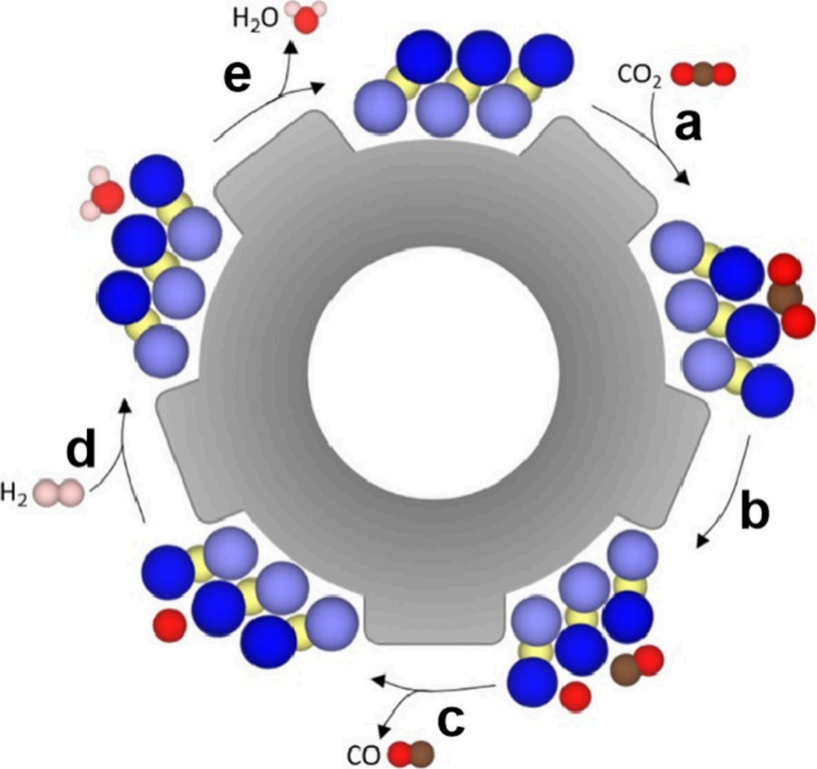
Elementary steps of the reverse water gas shift reaction calculated on bare surface Ti_3_C_2_. The mechanism involves: (a) CO_2_ adsorption, (b) CO_2_ dissociation, (c) CO desorption, (d) hydrogenation of the surface O* species and water formation, and (e) H_2_O desorption, closing the catalytic cycle. Reproduced with permission from ref. 103. Copyright 2021 American Chemical Society.

##### 3.1.2.4 X Vacancy-Induced Defect Sites

Due to their more convenient synthesis, most studies on the use of MXenes as catalysts focus on carbides, while the application of carbonitride or nitride MXenes remains much less explored. Carbonitrides can be obtained from carbide MXenes by nitridation using NH_3_ under controlled conditions. This well-established process demonstrates the possibility of exchanging C atoms of MXene interlayers. In other applications, such as the use of MXenes as photocatalysts, CO_2_ evolution has been observed during the reactions, indicating that some C atoms from the carbide layer become oxidized.^71^ Besides CO_2_, the evolution of CH_4_ has also been reported,^105^ again suggesting the formation of C vacancies.

It appears that X atoms can participate in catalysis by exchanging with other elements present in the medium, particularly N, through a mechanism that resembles the Mars–Van Krevelen process. In this pathway, N is first mobilized from the MXene structure to the product and then replenished by N_2_ from the gas phase. Notably, the catalytic activity of Mo_2_CT_x_ supporting Co NPs for N_2_ hydrogenation to NH_3_ is considerably enhanced when Co­(NO_3_)_2_ is used as a precursor, likely due to the incorporation of N atoms into the X layer (Figure 18).^106^ This observation provides compelling evidence that the X layer is not merely a passive structural component but can actively contribute to the reaction mechanism. Hence, this type of mechanism opens interesting opportunities to form organic products, mainly C1, and nitrogen compounds that still require development.

**Figure 18:**
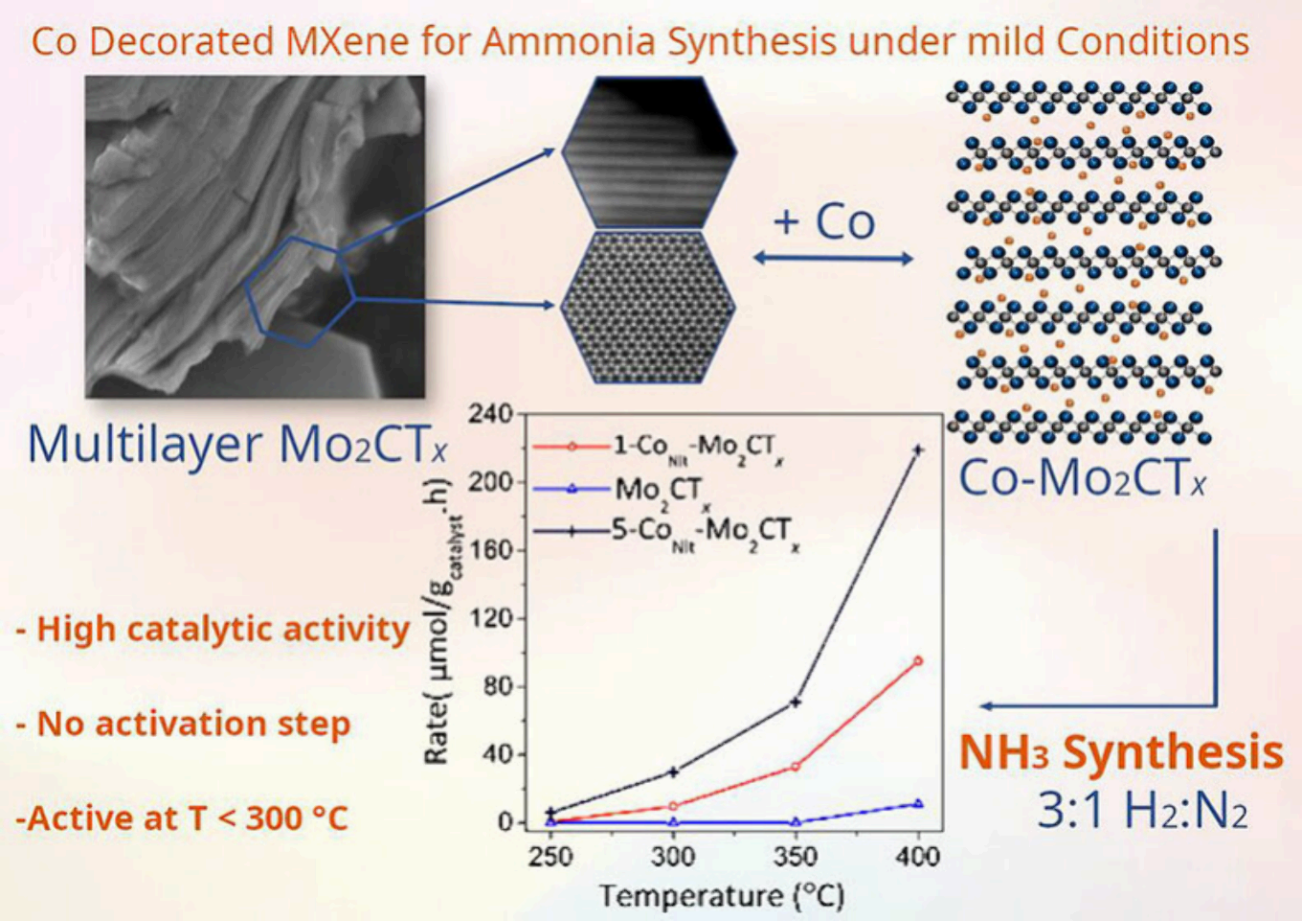
Co NP decorated Mo_2_CT_*x*_ catalyst for NH_3_ synthesis under mild conditions in which the positive influence in the catalytic activity of the presence N in the material has been observed. Reproduced with permission from ref. 106. Copyright 2024 American Chemical Society.

##### 3.1.2.5 Oxygen Vacancy-Induced Defect Sites

As previously mentioned, the stability of MXenes as catalysts under oxidizing conditions is a matter of concern due to their tendency to transform into the corresponding metal oxides. However, these derived metal oxides can possess a significant density of oxygen defects, resulting in 2D nanomaterials with notable intrinsic catalytic activities. One representative example is the oxidative dehydrogenation (ODH) of ethane by Ti_3_C_2_.^107^ During the initial stage of the reaction, a progressive increase in ethane conversion and a carbon balance exceeding 100% were observed.^107^ This phenomenon is attributed to the oxidation of the MXene carbide layer under oxidative atmospheres at high temperatures. TGA of chemically etched Ti_3_C_2_ MXenes under air typically reveals a weight increase of around 600 °C, corresponding to their complete oxidation into TiO_2_ (Figure 19a).^107^

**Figure 19:**
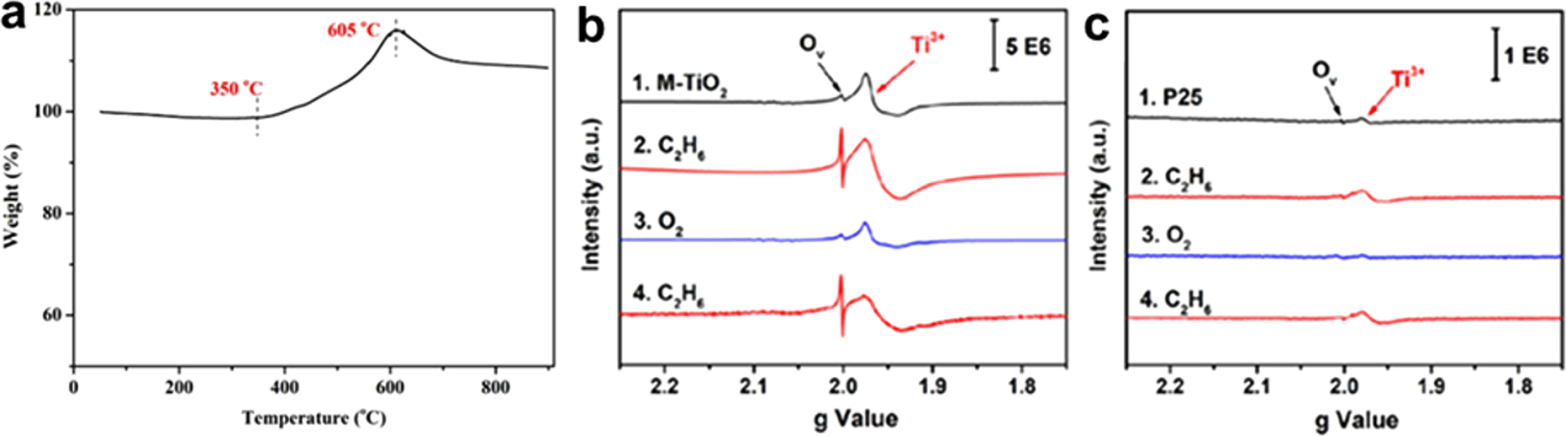
(a) TGA of Ti_3_C_2_T_*x*_ MXene under air conditions with a flow rate of 30 mL min ^–1^. Quasi *in situ* EPR spectra of (b) M-TiO_2_ and (c) P25 after the subsequent treatment with various gases: (1) ODH feed gas, (2) 10 vol % C_2_H_6_/He, (3) 5 vol % O_2_/He, and (4) 10 vol % C_2_H_6_/He at 600 °C for 0.5 h. Reproduced with permission from ref. 107. Copyright 2021 American Chemical Society.

Full transformation of Ti_3_C_2_T_*x*_ under ODH conditions (feed gas: 10 vol % C_2_H_6_ and 5 vol % O_2_ at 600 °C) leads to the formation of a TiO_2_ material with layered morphology, referred to as M-TiO_2_, which contains abundant Ti and oxygen vacancy defects.^107^ Importantly, the catalytic activity of M-TiO_2_ was primarily ascribed to these Ti and O vacancy defects.^107^ Ti vacancies are particularly effective in improving the reducibility of lattice O, thereby lowering the activation of energy for ethane conversion. The process operates through a Mars–Van Krevelen mechanism: O vacancies generated by the reaction of ethane with the lattice O are replenished by O_2_ from the gas phase, thus sustaining the catalytic cycle for the conversion of ethane to ethylene.^107^ It is noteworthy that this mechanism does not require the complete transformation of Ti_3_C_2_T_*x*_ that could partially survive in the process. However, the proposed active sites are associated with M-TiO_2_ rather than with the original MXene phase.

The presence of oxygen defects is very important in the catalytic activity, and it was confirmed by Raman spectroscopy: M-TiO_2_, formed *in situ* from Ti_3_C_2_T_*x*_ oxidation, exhibited shifted and broadened Raman bands of the anatase and rutile phases compared to highly crystalline P25.^107^ EPR spectroscopy also provides insights into defective structures. An intense signal at *g*_*xx*_ = *g*_*yy*_ = *g*_*zz*_ = 1.9875 observed in Ti_2_CT_*x*_ MXene is indicative of Ti^3+^ centers.^108^ These centers, resulting from O vacancies, facilitate the formation of nucleophilic oxygen species. A comparison of the EPR spectra between TiO_2_ derived from MXenes and commercial P25 highlights the presence of O vacancies as well as the existence of Ti^3+^ species when this oxide is derived from Ti_2_CT_*x*_.

Quasi *in situ* EPR spectroscopy of defect-rich M-TiO_2_ reveals a typical redox cycle under different reaction atmospheres, consistent with the Mars–Van Krevelen mechanism (Figures 19b,c). Upon treatment with C_2_H_6_, the signal associated with oxygen vacancies intensifies, suggesting that lattice oxygen is readily extracted to react with ethane, thereby generating additional oxygen vacancies. These materials exhibit properties like reducible metal oxides. In summary, while the instability of Ti_3_C_2_T_*x*_ and the fact that active sites lie outside the MXene structure may seem disappointing, a positive aspect is that Ti_3_C_2_T_*x*_ can serve as a precursor to highly defective TiO_2_ material with promising catalytic properties. Thus, although it can be argued that the strategy of using MXenes as precursor of oxides lacks interest due to alternative, common procedures of metal oxide synthesis, it could be possible, as in the case commented, that the unique properties of the MXene-derived oxide would justify exploiting further MXene oxidation.

Lattice oxygen can serve as an active site in metal-based oxidation catalysts. For example, in reactions such as selective CO oxidation or CH_4_ combustion, lattice oxygen is extracted from the metal oxide surface to oxidize reactants, forming metal–oxygen bonds.^107^ This enables the catalyst to oxidize reactants without requiring external oxygen in the initial stages of the reaction.

Defect engineering in MXenes, including vacancies of metal (M) sites, carbon/nitrogen (X) sites, surface terminations (T), and oxygen in derived oxides, is a key factor in regulating their catalytic performance. These defects, whether intrinsic to the synthesis procedure or induced postsynthetically, create under-coordinated or electronically perturbed sites that can serve as active centers or anchor sites for catalytically active species. While M and T vacancies enable cooperative or Lewis-type interactions with reactants, X vacancies may engage in Mars–Van Krevelen-like mechanisms, and oxygen-deficient oxide derivatives of MXenes offer alternative redox-active surfaces. Importantly, the formation, distribution, and reactivity of these defects are highly sensitive to synthetic parameters and operating conditions, emphasizing the need for precise control and advanced characterization. Harnessing these defect structures effectively will be key to unlocking the full catalytic potential of MXenes in catalysis.

#### 3.1.3 MAX Phase as Catalyst

To overcome the limitations associated with MXene instability, the catalytic activity of MAX phases for ODH has been explored.^111^ For the ODH reaction to occur using Ti_3_AlC_2_ as a catalyst, oxygen adsorption on the surface is mandatory to generate active oxygen species that react with hydrocarbons. In this context, the presence of defects may facilitate oxygen adsorption and thereby enhance the catalytic activity of MAX phases in ODH reactions. The presence of such defects in Ti_3_AlC_2_ can be characterized using high-resolution transmission electron microscopy (HRTEM), where domain, layered, and point defects can be visualized. Additionally, positron annihilation lifetime spectroscopy is a powerful technique that enables detection of internal voids and vacancy defects, as positron trapping in these defects leads to prolonged lifetimes within the characteristic time scale.

In one study, positron annihilation lifetime profiles were analyzed to determine the types and relative quantities of defects in oxidized MXenes, MXenes, and MAX phases. By analyzing the kinetics of positron-electron annihilation and detecting the corresponding g-ray decay from trapped positrons, the position and types of defects can be distinguished. The ratio of relative intensities provides information on the abundance of each defect type. In general, the g-ray signal lifetime is fitted to two components, τ_1_ and τ_2_, corresponding to two types of defects, with relative intensities *I*_1_ and *I*_2_. The shorter lifetime τ_1_ is ascribed with defects in the bulk, while the longer lifetime τ_2_ is ascribed to surface or subsurface defects of the materials. For Ti_3_C_2_ prepared using a 40 wt % HF solution, τ_2_ is approximately 374 ps, suggesting the presence of surface defects. Ti_3_C_2_ displays a shorter τ_1_ compared to Ti_3_AlC_2_, which is attributed to its ultrathin structure and minimal bulkiness. Furthermore, the *I*_2_/*I*_1_ ratio for Ti_3_C_2_ is 2.5 times higher than that of Ti_3_AlC_2_, indicating that HF exfoliation increases the density of surface defects.^97^ Oxidized Ti_3_C_2_ displays an even higher *I*_2_/*I*_1_ ratio, signifying a greater concentration of surface defects than either Ti_3_C_2_ or Ti_3_AlC_2_.^97^

For Ti_3_AlC_2_-catalyzed ODH, the absence of lattice oxygen has led to propose that surface defects are responsible for the formation of a thin surface layer of Ti__1_–y_Al_*y*_O__2_–y/2_ enriched with oxygen vacancies.^111^ The formation of this catalytically active layer was confirmed by high-angle annular dark field-scanning transmission electron microscopy (HAADF-STEM) imaging with associated energy-dispersive X-ray spectroscopy (EDX) elemental mapping (Figure 20).^111^ Furthermore, N_2_O chemisorption combined with XPS was employed to quantify oxygen vacancy concentrations. To generate these vacancies, the MAX phase was prereduced to 500 °C under an ultrahigh vacuum, creating defects that were subsequently exposed to N_2_O. Metallic sites in the material can dissociate adsorbed N_2_O at appropriate temperatures, oxidizing the metal, and enabling quantification of the oxygen vacancy population.^111^

**Figure 20:**
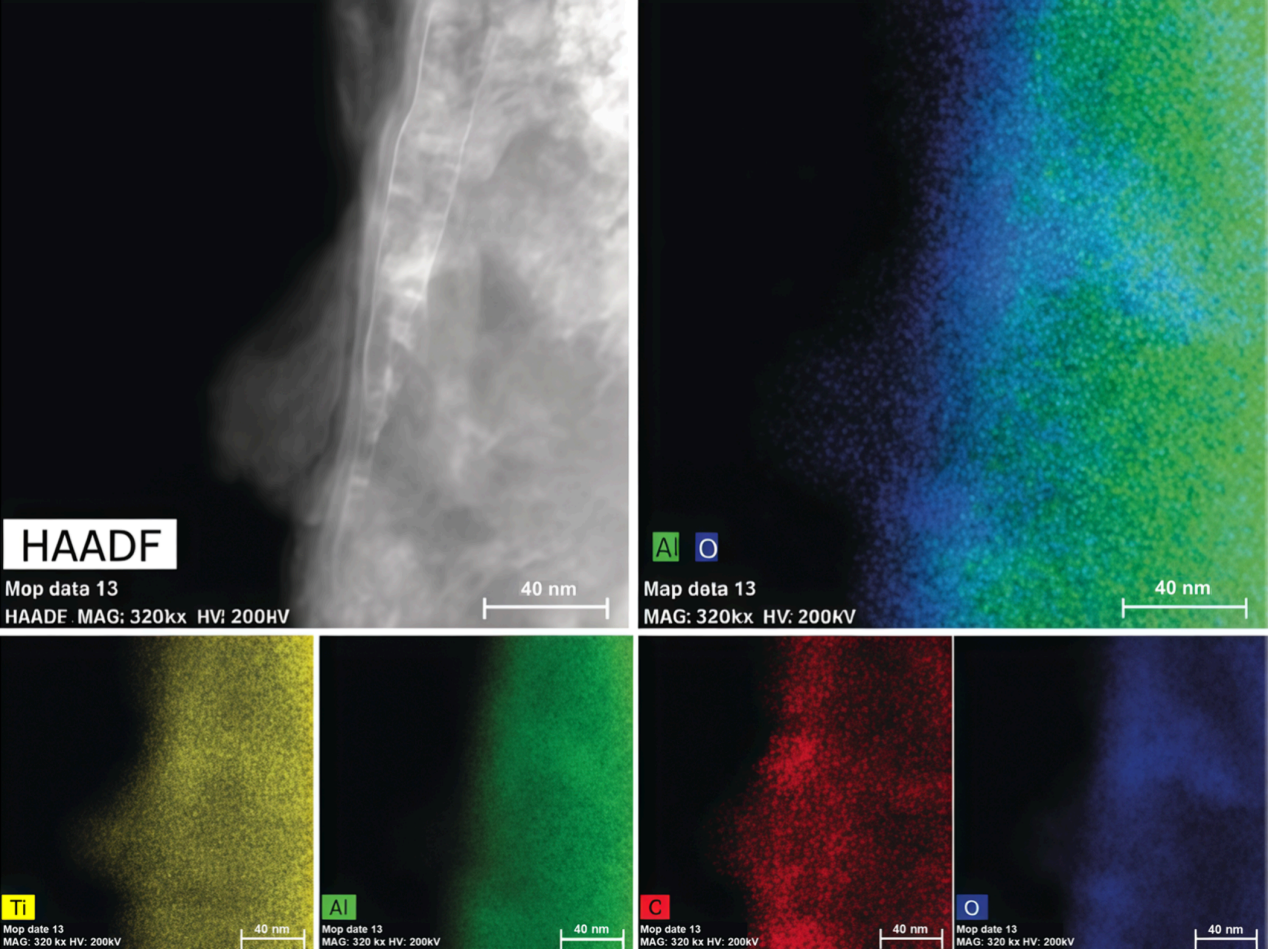
HAADF-STEM image (top left) and elemental maps for Al+O (top right) and Ti, Al, C, and O on the Ti_3_AlC_2_ MAX phase (bottom). Reproduced with permission from ref. 111. Copyright 2018 Wiley.

The catalytic activity of Ti_3_AlC_2_ was tested in the ODH of butane, resulting in the formation of a mixture of butenes and butadiene. The active sites are proposed to be oxygen vacancies on the external Ti_1–*y*_Al_*y*_O_2–*y*/2_ overlayer, which enables efficient operation of a Mars–Van Krevelen mechanism.

In the case of Ti_3_AlC_2_ MAX phase, positron annihilation spectroscopy and *in situ* TEM image have revealed a high density of surface and subsurface defects that correlate with catalytic activity. These findings suggest that MAX phases, when properly activated, can serve as viable catalysts or redox-active supports, expanding their functional role beyond MXene synthesis.

In summary, MXenes exhibit a rich diversity of catalytically active sites, ranging from surface functional groups and structural defects that may consist in vacancies of metal (M), nonmetal (X), and termination (T) atoms. These sites are unavoidably introduced in the etching process of MXene synthesis, but they can be later altered via postsynthetic modifications. Among these sites, metal vacancies are particularly significant—not only for providing direct catalytic activity but also for stabilizing SAs or metal clusters with high dispersion as discussed later. Surface terminations, though often chemically inert, can be tuned or partially removed to create new reactive sites, enabling acid–base catalysis and oxidative or reductive reactions. In addition, the transformation of MXenes under catalytic conditions, such as oxidation into defect-rich metal oxides, opens alternative pathways for activity through dynamic phase evolution and they can serve for the preparation of catalytically active MXene derivatives. Collectively, a comprehensive understanding and rational engineering of these active centers, supported by advanced characterization techniques such as FT-IR, XPS, EPR, XAS, and *in situ* Raman spectroscopy, are essential for unlocking the full potential of MXenes in thermal and photothermal catalysis. By establishing structure–activity relationships, advanced MXene catalysts can be prepared by intentional modification of their preparation procedure or applying postsynthetic treatments.

### 3.2 Characterization of MXenes as Supports

MXenes distinguish themselves from conventional supports (such as oxides, carbons, and nitrides) through their ability to combine metallic conductivity, tunable surface chemistry, and structural flexibility within a 2D framework. These unique characteristics endow them with exceptional potential as catalyst supports.

First, their high electrical conductivity facilitates ultrafast charge migration across the interface, minimizing electron–hole recombination and improving turnover rates in both thermal and photocatalytic reactions. Second, the presence of adjustable surface terminations (−O, −OH, −F, −Cl, etc.) provides a rich platform for chemical bonding and coordination with metals, enabling fine control over metal oxidation states and dispersion.^112^ Third, abundant M-site vacancies and structural defects act as robust anchoring centers for SAs or small clusters, preventing sintering and ensuring long-term stability. Fourth, the atomic thinness and high surface accessibility of MXene layers allow a large fraction of the surface to participate in catalysis, while their high thermal conductivity supports efficient heat dissipation under reaction conditions. Fifth, the strong MSIs characteristic of MXenes, driven by charge transfer and orbital hybridization, further tune interfacial electronic structures and optimize adsorption energies and reaction barriers. Finally, the dynamic redox nature of transition metal carbides and nitrides enables the formation of adaptive interfacial phases during reaction, a property rarely accessible in inert supports. Collectively, these features rationalize why MXenes outperform many classical supports in both activity and stability.^62^

#### 3.2.1 M Vacancies as Nests for the Immobilization of Metal Atoms, Clusters, and NPs

Besides their active role in catalysis, M vacancies can also play a crucial passive role in enhancing MXene functionality as supports, especially in SAC. M vacancies provide ideal anchoring sites that allow metal atoms to bond directly with the MXene carbon atoms, forming strong metal–carbon bonds that prevent agglomeration. This stabilization ensures high dispersion of SAs, maximizing their catalytic activity while maintaining accessibility to reactants. Furthermore, the M-deficient MXene surface possesses a high reducing capability, enabling it to reduce metal precursors directly upon contact without requiring additional reducing agents, even non-noble metals. This facilitates the synthesis of SACs through a straightforward, room-temperature process. The vacancies thus enable simultaneous metal cation adsorption and reduction, making it an efficient and environmentally friendly approach to synthesizing SACs. Additionally, surface-localized vacancies in 2D MXenes are fully accessible to reactants, allowing for better interaction with gaseous molecules like CO_2_ and increasing catalytic efficiency. Advanced techniques such as XAS and HAADF-STEM have confirmed the atomic dispersion and coordination environment of metal atoms in these systems. Altogether, the unique structural and electronic properties of M-deficient MXenes make them very suitable platforms for supporting highly active and stable catalytic centers.

In one example, insights into the structure, oxidation state, and bonding environment of single Pt atoms on Ti_3–*x*_C_2_ MXene were obtained using XAS.^113^ XANES at the Pt L_3_-edge indicated that the oxidation state of Pt in Pt/Ti_3–*x*_C_2_ lies between 0 and +4, as the absorption edge is located between those of metallic Pt and PtO_2_, indicating a partial positive charge on the Pt atoms.^113^ In addition, the higher edge energy compared to metallic Pt indicates electron transfer from the MXene to the Pt atoms, consistent with electron donation from the carbide layer to Pt. This electron redistribution, facilitated by the Ti_3–*x*_C_2_ support, stabilizes Pt in a partially oxidized state, which is beneficial for catalytic applications.^113^ EXAFS data provide information about the local bonding environment of these Pt SAs. The Fourier transform of the EXAFS typically displays peaks that correspond to specific bonds and coordination environments. In that way, for Pt­(SA)/Ti_3–*x*_C_2_, two main peaks at approximately 1.5 and 2.2 Å were attributed to Pt–C and Pt–Ti bonds, respectively, confirming that Pt atoms are atomically dispersed and occupy Ti vacancy sites, forming stable bonds with surrounding C and Ti atoms on the MXene surface. Particularly relevant is the absence of Pt–Pt contributions at about 2.8 Å, which is consistent with the lack of Pt NPs or clusters and confirms true atomic dispersion on the MXene, a feature that is crucial for maximizing metal utilization and catalytic performance.

Similarly, Ti vacancy defects created during Ti_2_AlN etching served as anchoring sites for Co SAs, preventing their agglomeration. This anchoring can be simply achieved by sonicating an aqueous MXene dispersion containing Co^2+^ salts and is critical for stabilizing Co in the SA form, thus optimizing catalyst active sites. Ti vacancies provide well-defined coordination sites that increase the binding energy between metal atoms (e.g., Co, Cu, Fe) and the MXene surface, effectively suppressing migration and clustering that commonly limit the stability of single-atom catalysts on conventional supports. In addition to XAS, high-angle annular dark field aberration-corrected scanning transmission electron microscopy (HAADF ac-STEM) can help visualize Co atoms anchored within metal vacancies in MXenes. This method, complemented by EDX spectroscopy, provides atomic-level imaging, confirming SA nature. However, given the similar atomic masses of Ti and Co, it is always advisible to complement HAADF ac-STEM with XAS analyses, which offer more conclusive evidence regarding the coordination environment of Co with Ti, N, and surface functional groups. In this regard, the conclusion that Co does not coordinate with the nitrogen present in the Ti_2_N structure but it is embedded within Ti vacancies would be more convincingly supported by additional XAS data.^114^

#### 3.2.2 Metal–Support Interactions

Metal dispersion is a critical factor in achieving high activity per metal atom. Interaction with the support is a widely used strategy to prevent the agglomeration of metal NPs under reaction conditions, thereby maintaining a high number of exposed, catalytically active metal atoms. Achieving strong MSI has been a long-standing objective in heterogeneous catalysis involving supported metal and metal oxide NPs and clusters. MSIs play a pivotal role in determining the dispersion, stability, and catalytic performance of metal species supported on MXenes. These interactions range from weak physical adsorption to strong electronic coupling and even reactive alloy formation at the interface. This MSI, arising from electron density transfer and *van der Waals* forces, can stabilize NPs and clusters against sintering, influence their morphology, creating catalytically active interfaces, and allowing fine-tuning of catalytic performance. In this context, the use of MXenes as supports has drawn considerable attention, since they can precisely confine and stabilize SACs. By selecting the appropriate MXene type and metal adatom, the catalytic properties of MXene-based materials can be tailored for various catalytic reactions.

Different MXene hosts exhibit distinct MSI behaviors, which can now be correlated quantitatively with their intrinsic electronic structures and chemical compositions. For instance, Ti_3_C_2_T_*x*_, owing to its moderate work function and strong oxyphilicity, facilitates electron donation to supported metals but is prone to oxidation; Nb_2_CT_*x*_, with its higher work function and metallic nature, promotes stronger charge transfer and interfacial alloying, favoring the stabilization of metallenes and intermetallic interfaces; while Mo_2_CT_*x*_, being more reducible and carbide-like, provides an electron-rich environment conducive to forming reactive interfaces for hydrogenation and CO_2_ reduction. The relative alignment of Fermi levels, surface terminations, and defect densities among these MXenes dictates the strength and direction of charge transfer, ultimately defining the nature and durability of the MSI.^115^

Quantitative computational studies provide strong support for the magnitude of charge transfer. For example, first-principles calculations on transition-metal adatoms on M_2_C/M_2_CO_2_ MXenes report Bader charge transfers in the range of ∼ 0.2 to 0.6 e^–^ per adatom and adsorption energies of 2–6 eV, with stronger adsorption correlating with larger electron transfer to the MXene surface.^116^ Moreover, the magnitude of charge transfer correlates with changes in the *d*-band center of the supported metal and thereby modifies adsorption energies of catalytic intermediates—thus establishing a causal link between charge transfer, electronic structure modulation, and enhanced catalytic activity. For instance, a more negative *d*-band center (due to electron donation) weakens CO adsorption, reducing poisoning and enhancing turnover.^117^

From these comparative observations, several governing principles can be summarized: (i) the work function and Fermi-level alignment between metal and MXene determine the equilibrium charge flow and the oxidation state of active sites; (ii) the type and density of surface terminations modulate the coordination environment and anchoring energy of supported species; (iii) oxyphilicity and reducibility of the MXene M element govern the likelihood of reactive MSI and alloy formation; (iv) vacancy formation energy and local defect topology dictate whether isolated atoms, clusters, or 2D metal layers are stabilized; (v) the high thermal and electrical conductivity of MXenes facilitates efficient electron–phonon coupling and interfacial heat transfer; and (vi) structural accessibility, especially prevention of restacking, maximizes utilization of interfacial sites. These principles provide a unified framework for the rational design of MXene–metal interfaces with targeted functionalities.

In addition, a preliminary classification of metal/MXene combinations with strongest MSI can be proposed. Late transition metals with high d-orbital occupancy (e.g., Ni, Co, Pd, Pt) typically exhibit stronger interaction with early transition-metal-based MXenes (e.g., Ti_3_C_2_, Nb_2_C) because of favorable Fermi-level alignment and orbital overlap. In contrast, more electropositive metals (e.g., Sc, Y) show weaker interaction and lower charge transfer. This trend is consistent with high-throughput DFT screening, which found that metal adatom Bader charges correlate with adsorption energies across many MXene supports.^116,118^

The nature and strength of the interaction are influenced by surface functional groups, the reduction conditions, and the redox properties of the MXene. Characterization techniques such as XPS, XAS, and H_2_-TPR provide critical insight into the oxidation state, coordination environment, and surface reducibility, thereby guiding the rational design of metal–MXene interfaces. Importantly, under appropriate thermal treatment, these interactions can drive the transformation of 3D metal NPs into 2D metallenes or trigger reactive MSIs that yield bimetallic interfaces with superior catalytic activity.

Depending on the surface functional groups, a high density of sites with strong anchoring to the MXene structure can be obtained. First-principles calculations predicted that certain configurations of transition metals on O-terminated MXenes, especially those involving Sc and Ti, display high adsorption energies.^119^ Higher binding energies between metals and the MXene surface generally correlate with increased charge transfer from the metal to the MXene. Transition metals like Sc and Ti show particularly strong interactions due to this electron transfer, enhancing their stability. Although these calculations suggest that bare MXenes provide the strongest MSIs, O-terminated MXenes still provide adequate anchoring for certain transition metals, making O-functionalized surfaces a practical, albeit less effective, alternative to anchor metals on MXenes.

Strong MSIs can also be established by high-temperature treatments, during which metal species anchored on the MXene surface may undergo migration and restructuring from highly reducible oxides. Further temperature increases may induce alloying between the incorporated metal and the M element of MXene. This alloying process is known as *reactive MSI*. Typically, such interactions are promoted under H_2_ atmosphere, which reduces the metal oxidation state and facilitates alloy formation with the MXene M component.

This section describes the characterization techniques used to identify and confirm the presence of MSIs. We focus on three types: (i) interactions between the metal and surface functional groups, (ii) interactions between the metal and the M element of the MXene resulting in strong MSIs, and (iii) reactive MSIs resulting in alloy formation.

##### 3.2.2.1 Interactions between the Metal and Surface Functional Groups

XPS is a surface-sensitive technique that specifically probes solid surfaces using soft X-rays. It provides information on the elemental composition, chemical states, and electronic structure of the elements present at the material surface. This technique complements the information provided by XAS, which yields similar information but from the bulk of the material rather than just the surface, as in the case of XPS.

One example illustrates the use of both XPS and XAS to investigate MSIs corresponding to Cu on Ti_3_C_2_T_*x*_. XPS is sufficiently sensitive to differentiate between oxidation states of elements (e.g., Cu^0^/Cu^I^ vs Cu^II^) based on characteristic shifts in binding energy. Determining the oxidation state is crucial for characterizing active sites involving transition metals, as their oxidation state significantly affects catalytic activity. In XAS, the two most important regions are the near-edge XANES and the extended (X-ray Absorption Fine Structure; EXAFS) absorptions, which provide complementary insights to those of XPS by probing the electronic and local structural environment of elements within a catalyst. On one hand, XANES offers an overview of oxidation state and electronic structure, in which shifts in the absorption edge position reflect changes in oxidation state, while the shape and intensity of its extended absorption features provide information about unoccupied electronic states and coordination symmetry.

In that context, the interactions between copper SA (Cu-SA) supported on Ti_3_C_2_T_*x*_ synthesized via molten salt etching of the corresponding Ti_3_AlC_2_ precursor were studied by XPS and XAS. The XPS data revealed binding energy peaks indicative of a mixed Cu^I^ and Cu^II^ oxidation state, confirming the stabilization of Cu in that form. To further assess the strength of interactions between the Cu atoms and the MXene support, XANES and EXAFS analyses at the Cu K-edge were critical for understanding the local atomic structure and bonding environment of Cu. XANES confirmed that Cu existed in a mixed +1/+2 oxidation states. EXAFS data revealed that Cu was coordinated to three oxygen atoms, forming stable active sites suitable for APS activation, thereby indicating strong interactions between the isolated Cu atoms and the O-terminated Ti MXene surface.^120^ This example highlights the complementary role of XPS and XAS in elucidating MSIs in MXene-based catalysts.

The surface of MXenes is generally enriched with functional groups, such as −F, which are crucial for maintaining structural integrity and dispersing supported metals. In some cases, these surface groups can also chemically react with the supported metal. For instance, when Ti_3_C_2_T_*x*_ is combined with Pr­(NO_3_)_3_ and subjected to hydrothermal treatment, PrF_3_ nanosheets form on the MXene surface, resulting in a highly active heterojunction catalyst with stable Pr^3+^/Pr^4+^ redox sites.^164^ The formation of PrF_3_ was confirmed by XRD, which showed distinct PrF_3_ diffraction peaks and the absence of other crystalline phases, confirming successful synthesis and integration of PrF_3_ onto the MXene surface. XPS data further supported this by identifying peaks corresponding to Pr^3+^ and Pr^4+^ oxidation states. The presence of both oxidation states aligns with PrF_3_ formation and confirms enhanced redox capability of the catalyst. Notably, the Pr^3+^/Pr^4+^ redox couple plays a central role in accelerating electron transfer within the catalyst, which is a critical step for improving dehydrogenation kinetics.

##### 3.2.2.2 Interactions between the Metal and the M MXene Component

Hydrogen treatment of Ti_3_C_2_ at 300 °C results in water loss and surface defunctionalization, primarily through the removal of −OH functional groups, which leads to a measurable decrease in interlayer spacing. H_2_-TPR analysis serves as an indicator of surface reducibility; lower temperatures of H_2_ uptake suggest easier reduction of the MXene surface.^121^ This process can effectively remove −OH species from the MXene surface.^122^ H_2_ reduction pretreatment is generally found to improve the catalytic performance of metals supported on MXenes, as it significantly promotes strong MSIs and facilitates electrons transfer at the metal–MXene interface. For instance, the interaction between Pt and the MXene support is significantly enhanced by H_2_ reduction pretreatment, which increases the proportion of surface oxygen (O*) relative to −OH groups and promotes the formation of Pt^2+^ species.^123^ These active centers play a critical role in facilitating low-temperature oxidation of benzene by promoting O_2_ activation and dissociation, leading to the conversion of benzene into CO_2_.^123^ The H_2_ reduction of MXenes increases the amount of mobile surface oxygen (vacant surface groups) and decreases the concentration of chemisorbed oxygen (O_II_). The observed catalytic activity after reductive pretreatment of Ti_3_C_2_ in oxidation reactions is mostly attributed to reactions between substrates and active oxygen species that remain on the MXene surface at reaction temperatures. SAs can occupy these vacant centers and assist with the diffusion of active O* species from subsurface or bulk layers. For instance, Pt sites on MXene have been shown to activate vacant oxygen sites and O_II_ species on the catalyst surface, enabling synergistically interactions with adsorbed benzene molecules and thereby improving catalytic activity in low-temperature benzene mineralization.^123^

H_2_-TPR profiles can also be used to assess the reducibility of metals supported on doped MXenes. In the case of N-doped MXenes, the profiles indicate that supported metals exhibit a lower reduction temperature compared to those on undoped MXenes (Figure 21).^124^ This improved reducibility is attributed to the modified electronic environment introduced by N doping, which enhances the interaction between the MXene support and the supported metal.^124^ This study, however, shows that thermal treatment can also promote the evolution of some CO and CO_2_, as well as CH_4_ (Figure 21).

**Figure 21:**
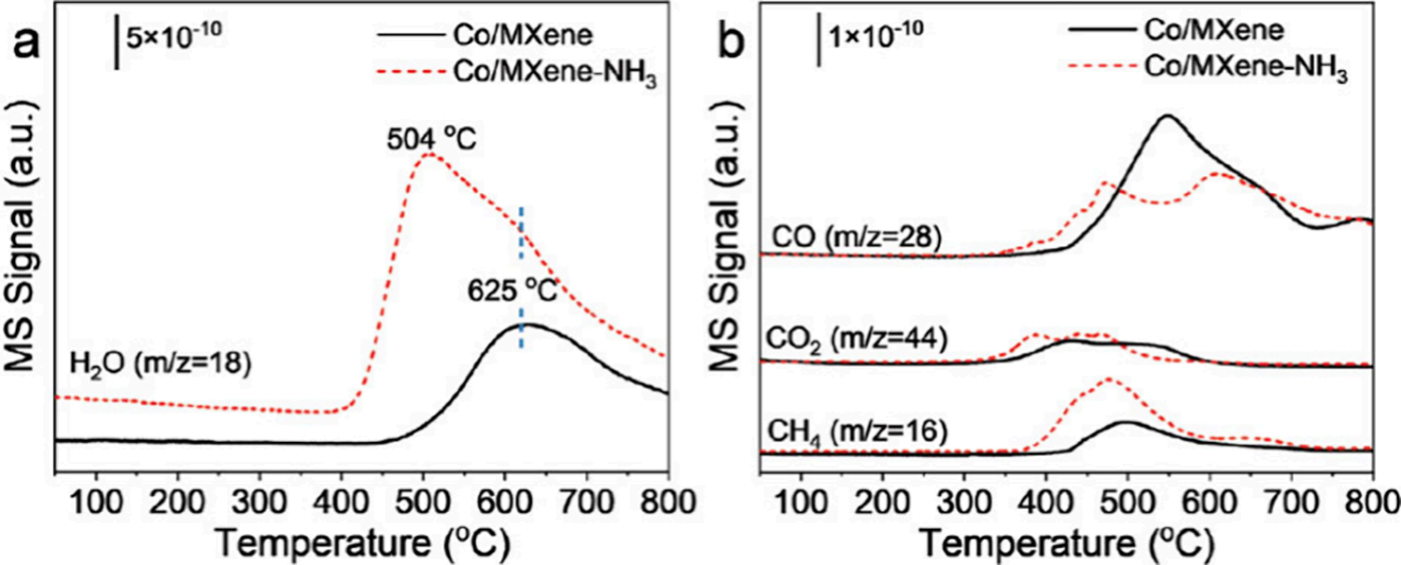
H_2_-TPR profiles of Co/MXene and Co/MXene-NH_3_ catalysts. (a) H_2_O mass spectra (*m*/*z* = 18), and (b) mass spectra of CO_2_ (*m*/*z* = 44), CO (*m*/*z* = 28), and CH_4_ (*m*/*z* = 16) showing the influence of N-doping on Co activity. Reproduced with permission from ref. 124. Copyright 2021 Wiley.

In general, reductive thermal treatments are employed to establish strong MSIs by cleaning the surface terminations, facilitating the formation of a metal nanolayer on the MXene surface. This thermal reduction process promotes electron transfer between the MXene and the supported metal and helps stabilize the 2D structure of the metal on the MXene. Under certain conditions, the reduction step can strip away surface terminations, leaving almost clean M surfaces that can strongly interact with the deposited metal.^125^ Strong MSIs serve as driving forces that control the growth of metal layers on the MXene surface in the form of a 2D heterostructure, frequently referred to as metallenes when the thickness corresponds to only a few atomic layers due to their morphological resemblance to graphene or MXenes.^126^ Metallenes are ultrathin 2D metallic nanostructures composed of one to several atomic layers, characterized by high surface atom exposure, abundant unsaturated coordination sites, and adjustable in-plane strain. Owing to their atomically thin morphology, they exhibit unique electronic configurations distinct from bulk metals or NPs. According to Wei et al.,^126^ MXenes can stabilize such 2D metallic layers through strong interfacial coupling and charge transfer, as exemplified by the formation of Pd metallenes on Nb_2_C via galvanic replacement. This ability highlights the potential of MXenes as versatile hard templates for engineering unique metal–MXene heterostructures with high stability and tunable reactivity.

The overcoating of the supported metal onto the MXene surface is a clear reflection of favorable MSIs that outweigh the cohesive forces between metal atoms. In this context, it was recently demonstrated that the presence of minimal surface functional groups on Nb_2_C helps stabilize Pd in the form of a 2D metallene structure, whereas the presence of functional groups such as -Br, -Cl, or -O favors the formation of NPs.^126^ This change in morphology is common across many supports and indicates that a bare MXene surface with no functional groups maximizes the interaction between Nb and Pd, thereby favoring the formation of 2D Pd overlayers. Through a galvanic replacement reaction, Nb^2+^ acts as a reducing agent for Pd^2+^ due to the lower reduction potential of the Nb^5+^/Nb^2+^ redox couple. When Pd^2+^ ions come into contact with the Nb_2_C surface, they are spontaneously reduced to Pd atoms, forming a thin and highly dispersed 2D configuration. Nb atoms in Nb_2_C interact strongly with Pd atoms, providing the necessary energy to overcome Pd–Pd cohesive forces that would otherwise promote the formation of 3D Pd NPs.^126^ Molecular dynamic simulations corroborate the experimental evidence, predicting that strong MSIs on bare Nb_2_C can induce a structural transformation of Pd from 3D NPs to 2D metallenes. Figure 22 illustrates this morphology change, which is indicative of strong MSI.

**Figure 22:**
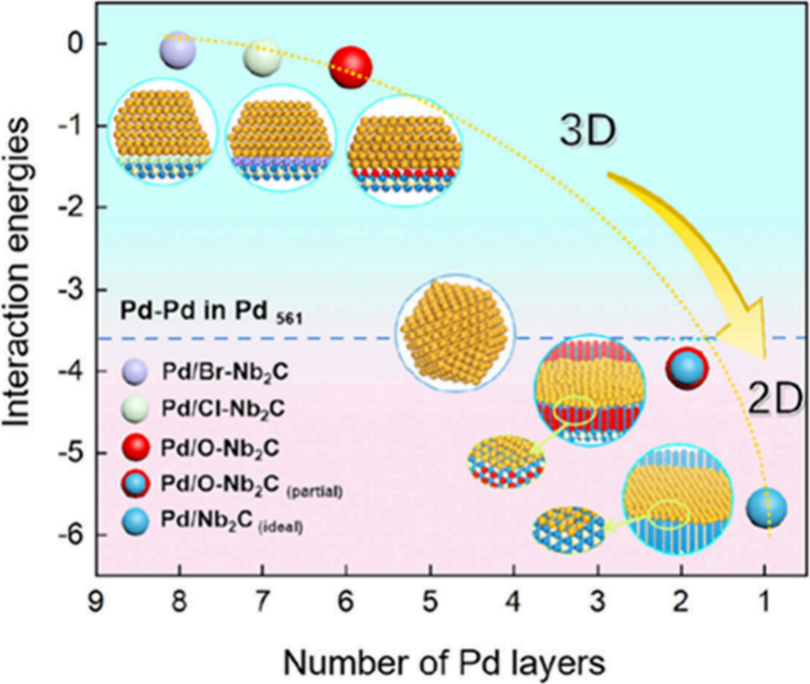
Relationship between the interaction energies between Pd and Nb_2_C supports and the number of Pd layers in the molecular dynamics simulations of Pd/MXenes catalysts. Reproduced with permission from ref. 126. Copyright 2023 Nature Portfolio.

##### 3.2.2.3 Reactive Metal–Support Interactions

As previously noted, the MSI in the case of MXenes can be strong enough to overcome metal–metal bond energy. In extreme cases, in which such interaction is particularly strong, a chemical reaction may occur at the interface between the early transition metal M of the MXene and the supported metal species. As previously commented when discussing the case of metallenes on MXenes, this chemical reaction is particularly likely upon removal of the terminal groups, which exposes the redox-active bare M atoms of the MXene structure to the incoming metal. Such highly reducible and reactive surfaces make MXenes promising candidates to undergo reactive metal–support interfaces, in which the introduction of another metal element can form ad-metal/interfaces with interesting catalytic activity. Characterization of these interfaces is of great interest, as they are believed to endow the material with specific catalytic performance compared to analogous systems lacking such interfaces.

A reactive MSI has been observed between Pt and Ti_3_C_2_, leading to the formation of the intermetallic compound Pt_3_Ti, which exhibits optimal catalytic sites for formaldehyde oxidation.^127^ The reactive interaction modifies the electronic properties of Pt by alloying it with Ti from the MXene structure, enhancing the Pt site effectiveness in converting volatile organic compounds (VOCs) into carbon dioxide.^127^ Furthermore, such interactions stabilize Pt_3_Ti alloy zones on the Ti_3_C_2_ support, creating a robust interface between the intermetallic phase and the MXene, thereby reducing NP aggregation. This stabilization is crucial under reaction conditions, as it may prolong catalyst lifespan by preventing deactivation.

Evidence for the formation of intermetallic compounds through reactive MSIs is typically obtained via XAS and XPS; in some cases, when the intermetallic phase is sufficiently abundant, additional confirmation is provided by XRD and HAADF-STEM. In one example, the reactive interaction between Pt and a Nb_2_CT_*x*_ support, and the associated changes in NP composition, were probed by *in situ* Pt L_III_-edge XAS and XPS.^47^ Compared to Pt supported on Al_2_O_3_, which is a nonreducible support, Pt supported on Nb_2_CT_*x*_ displayed a more intense and narrower XAS white line, indicating the absence of Pt–Pt bonds.^47^ EXAFS also revealed changes in the metal–metal distance region, where Pt–Nb interference weakened the Pt–Pt signal. Alloy formation was further confirmed by a positive shift in the Pt 4*f*_7/2_ binding energy in XPS, typically found in Pt-metal alloys such as Pt–Sn, Pt–Co, Pt–Ru, and Pt–Ti. Collectively, these characterization data support the formation of a Pt–Nb interface (Figure 23).

**Figure 23:**
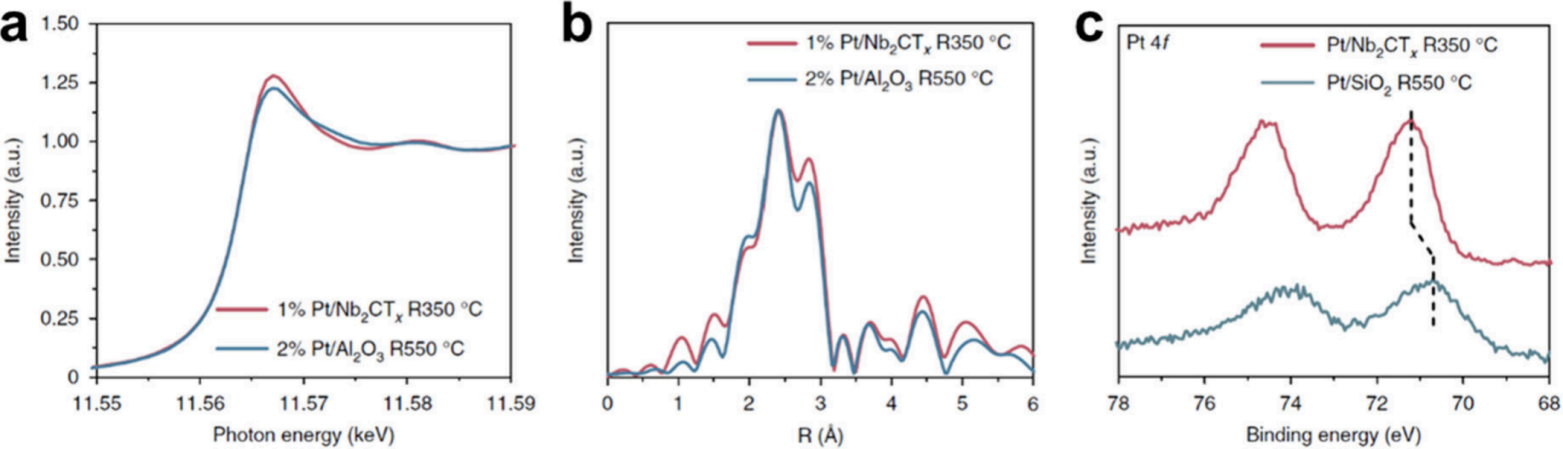
*In situ* XAS and quasi *in situ* XPS of the 1% Pt/Nb_2_CT_*x*_ catalysts. (a) *In situ* XANES spectra of the Pt L_III_ edge of the 2% Pt/Al_2_O_3_ sample treated at 550 °C and fresh 1% Pt/Nb_2_CT_*x*_ treated at 350 °C in 3% H_2_/He. (b) Fourier transform plot of the *k* ^2^ EXAFS for the 2% Pt/Al_2_O_3_ sample treated at 550 °C and for fresh 1% Pt/Nb_2_CT_*x*_ treated at 350 °C in 3% H_2_/He. (c) *Quasi in situ* XPS spectra of Pt 4f of Pt/SiO_2_ reduced at 550 °C and 1% Pt/Nb_2_CT_*x*_ treated at 350 °C. Reproduced with permission from ref. 47. Copyright 2018 Nature Portfolio.

Pt NPs appear to be supported on three-atom thick Nb_2_CT_*x*_ sheets. However, Pt layers near Nb undergo reaction, forming the interfacial region, becoming decomposed and generating discontinuities in the layered Nb_2_CT_*x*_ structure, as can be observed in TEM (Figure 24).^47^ After high-temperature reductive pretreatment, surface functional groups are removed, exposing free Nb sites on the Nb_2_CTx surface. Sacrificial layers, as observed in HAADF-STEM of Pt/Nb_2_CT_*x*_, are thought to result from this reactive MSI at the interface between the Nb MXene support and the Pt NPs. The newly formed terminal Nb atoms are in contact with the Pt–Nb surface alloy and form interfaces that exhibit strong affinity for H_2_O and -OH groups. In Pt/Nb_2_CT_*x*_, the active sites of the water–gas shift reaction are believed to be located at these metal–support interfaces, which facilitate H_2_O dissociation.^47^ The dynamic redox interchange between Nb^5+^ and Nb^4+^ species during reaction suggests the participation of lattice oxygen in a Mars–Van Krevelen-type mechanism, consistent with *in situ* XAS and XPS observations. The 2D architecture of Nb_2_CT_*x*_ further promotes this redox cycling by enabling short oxygen diffusion paths and efficient electron transport between Nb and Pt centers, distinguishing MXene-supported catalysts from bulk metal carbides. Since reactive MSIs stabilize and disperse NPs and increase the number of interfaces, they improve H_2_O coverage and make the Pt/Nb_2_CT_*x*_ significantly more effective for the water–gas shift reaction than Pt/Al_2_O_3_.

**Figure 24:**
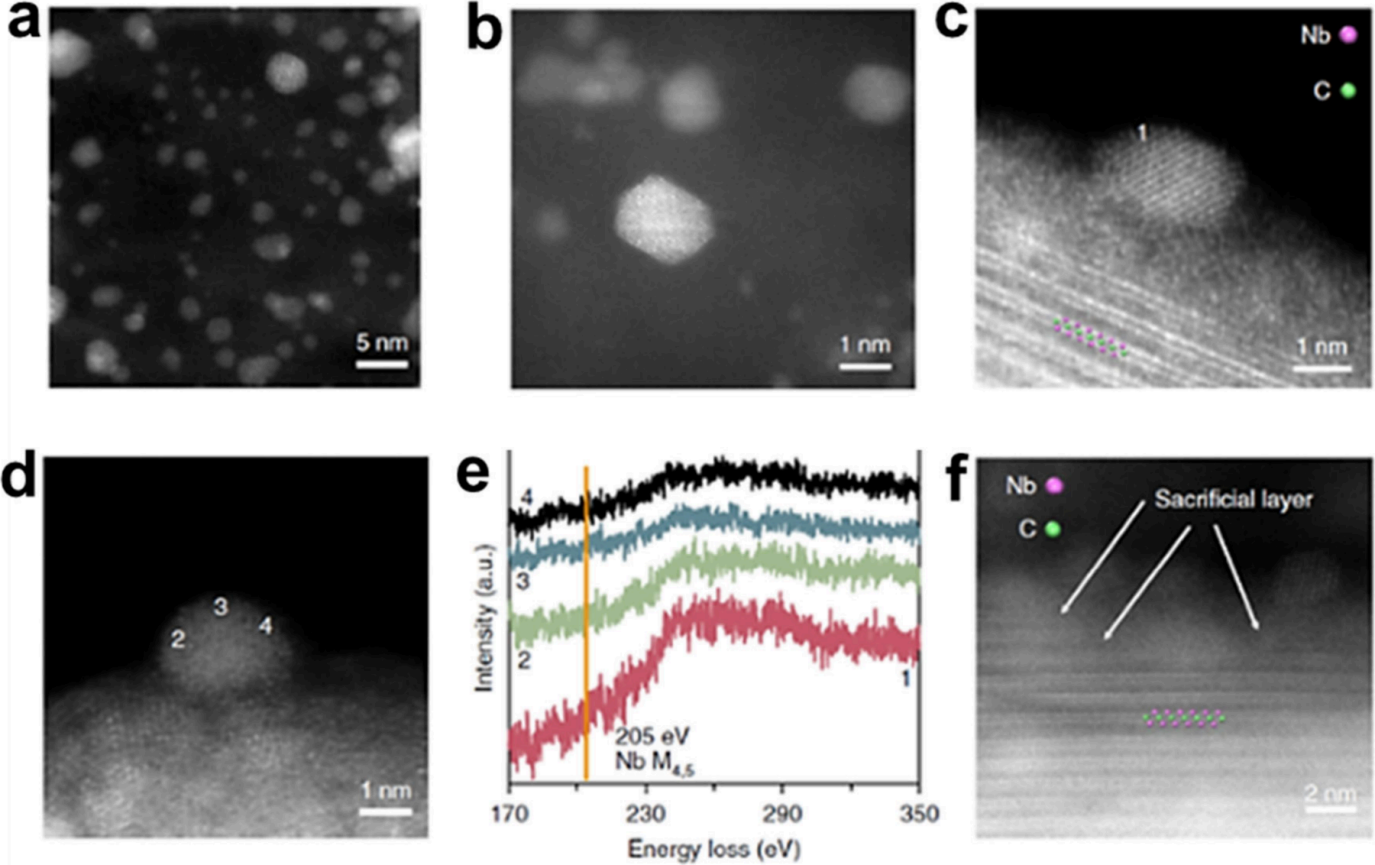
Electron microscopy and spectroscopy of the spent 1% Pt/Nb_2_CT_*x*_ catalyst. (a, b) HAADF-STEM images of the 1% Pt/Nb_2_CT_*x*_ catalyst after being used in the reverse water gas shift. (c, d) HAADF-STEM images of typical NPs supported by Nb_2_CT_*x*_ MXene. The majority of each particle is hanging over the vacuum to avoid niobium interference from the support. (e) EELS images acquired at several points on the particle surface, the locations of which are shown by corresponding numbers in (c) and (d, f), HAADF-STEM image showing discontinuous Nb_2_CT_*x*_ MXene layers. Reproduced with permission from ref. 47. Copyright 2018 Nature Portfolio.

One of the general problems of using MXenes as support for metal NPs is the limited accessibility to active sites. Prior to metal deposition, MXenes are generally delaminated using sonication or organic solvents. In these processes, the concentration of MXenes tends to be kept low to ensure colloidal stability. However, if subsequent thermal treatments are performed to reduce the metal, the MXene sheets tend to aggregate and collapse into a stacked structure. When layers are stacked, only the outer layers and edges remain accessible for reactions. In comparison, individual sheets expose both sides of each layer, enabling better interaction between the supported metal particles and reactant molecules. Evidently, unstacked materials should exhibit higher catalytic efficiency and faster reaction kinetics. Solid oxides have been reported to help prevent stacking and maintain exfoliated MXene sheets.^128^ In this way, dispersing Mo_2_CT_*x*_ nanosheets onto metal oxide particles leads to better separation and exposure of the metal sites on the MXene layers, maximizing the available surface area for substrate and reagent access. Thus, the main role of the metal oxide particles is to prevent excessive stacking of MXene layers, which could otherwise reduce the accessibility of active sites and limit the catalyst effectiveness. Figure 25 illustrates the concept of using metal oxides to prevent the stacking of MXene sheets.

**Figure 25:**
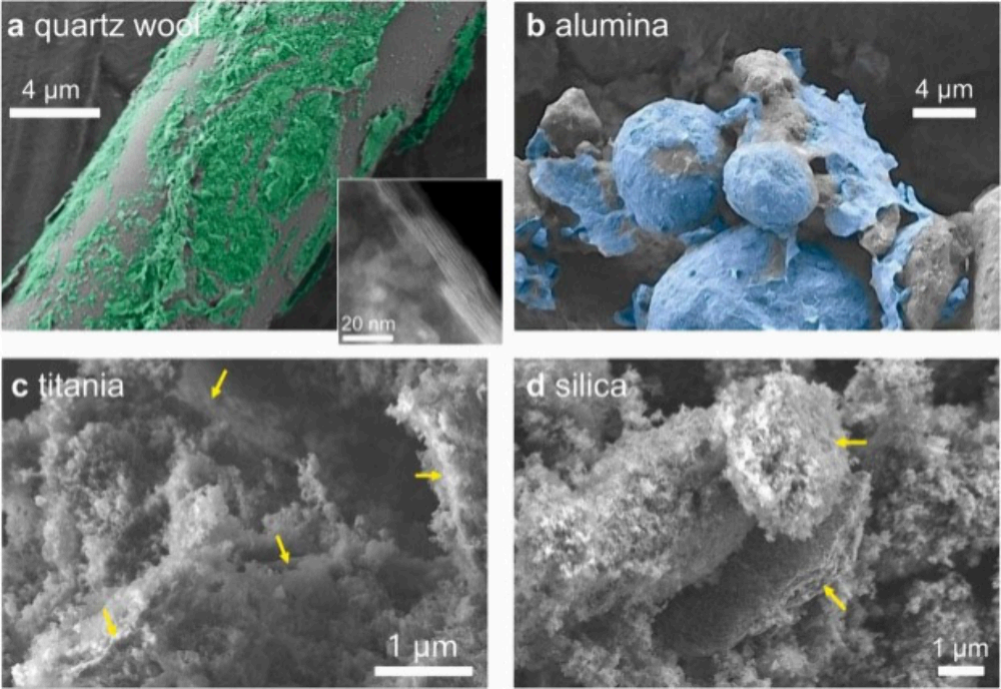
SEM characterization of unstacked catalysts. SEM images of 0.1Pt/Ti_3_C_2_T_*x*_ unstacked with several oxides as indicated in the image. The inset in (a) is an ADF-STEM image of MXene multilayers (0.1Pt/PMX2) deposited on quartz wool. Green and blue colors in (a) and (b) indicate MXene sheets or flakes. Arrows in (c) and (d) point to MXene flakes. Reproduced with permission from ref. 128. Copyright 2023 Elsevier.

In one representative example, silica was used as a support to disperse Mo_2_CT_*x*_ nanosheets, thereby increasing the accessible surface area for Cu loading on Mo_2_CT_*x*_ and ensuring higher catalytic activity. During the preparation, Cu preferentially migrated to Mo_2_CT_*x*_ under reductive conditions, with the presence of silica enhancing the stability and activity of the Cu–Mo_2_CT_*x*_ catalyst for CO_2_ hydrogenation.^129^ Copéret et al. reported that Cu was initially dispersed on both Mo_2_CT_*x*_ and silica; however, a preferential migration and enrichment of Cu onto the MXene surface occurred during the H_2_ reduction treatment. This favorable distribution was confirmed by EDX, which showed a higher intensity of the Cu signal on the Mo_2_CT_*x*_ sheets compared to the silica particles (Figure 26).^129^ This preferential dispersion was likely due to the strong interaction between Cu and the partially reduced Mo_2_CT_*x*_, which exhibited a higher affinity for Cu, particularly under reductive conditions at elevated temperatures. Altogether, MXenes represent an advanced support platform with tunable surface chemistry and strong metal-binding capability, suitable for developing next-generation heterogeneous catalysts.

**Figure 26:**
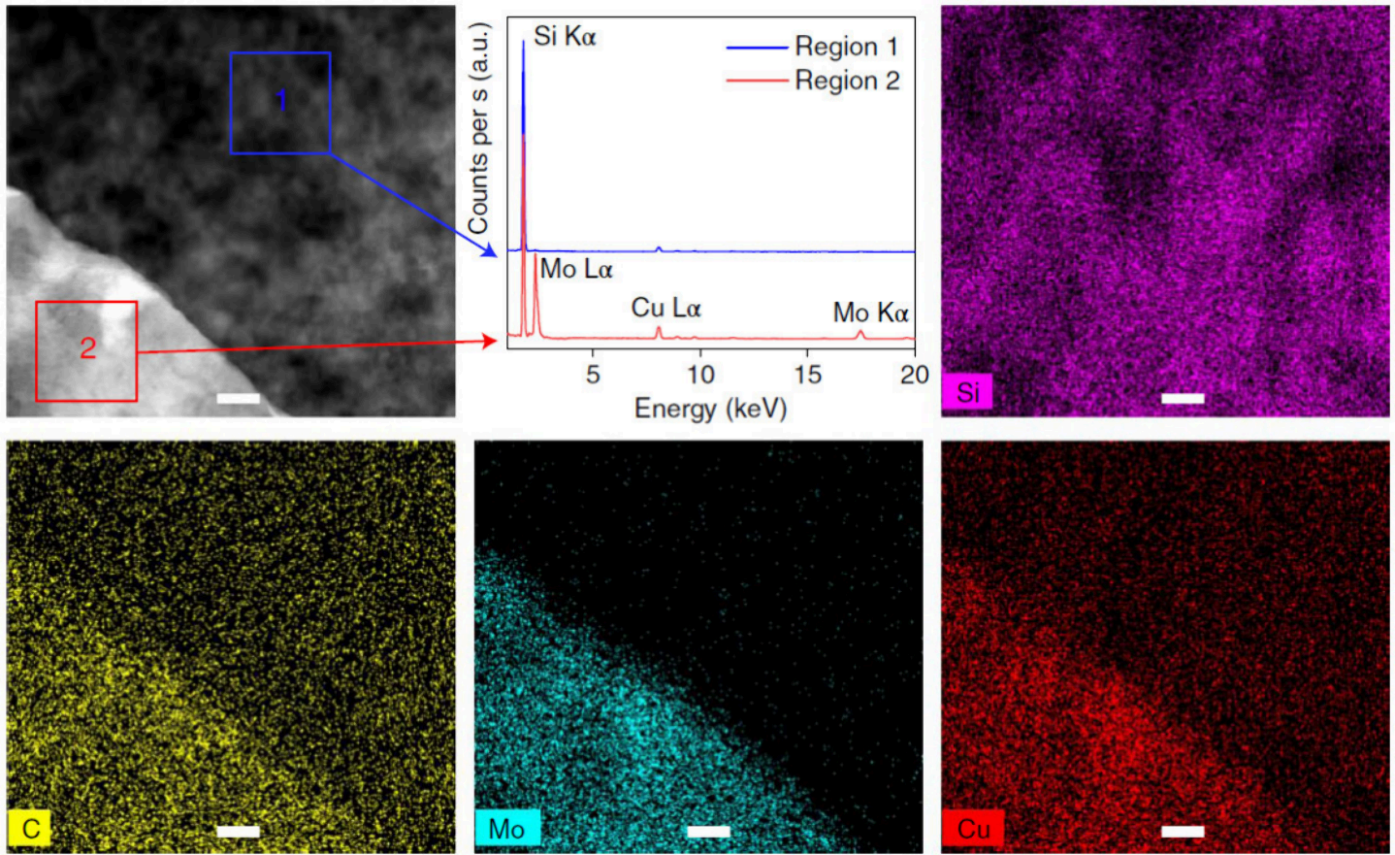
EDX showing the enrichment of copper on the MXene. TEM image (upper, left frame) marking two square regions in where elemental analysis was performed (upper, central panel) and corresponding elemental mapping of the TEM image for the elements indicated in eahc image. Reproduced with permission from ref. 129. Copyright 2021 Nature Portfolio.

Despite these rapid advances, the methodological rigor of MXene-based catalytic studies remains limited, with most works still at an early validation stage. Common shortcomings include the absence of leaching and heterogeneity tests, insufficient verification of true heterogeneous catalysis, and a lack of TOF normalization based on active-site quantification. To ensure reproducibility and comparability, future research should incorporate hot filtration or three-phase control tests, ICP-OES/ICP-MS analysis of postreaction solutions, and active-site quantification using CO chemisorption, XAS coordination-number fitting, or CO-DRIFTS titration. In addition, reporting initial rates, error margins, and turnover frequencies based on well-defined active sites should become standard practice. Complementary postreaction characterizations (e.g., XPS, HAADF-STEM, *in situ* XAS) are strongly recommended to confirm structural integrity and exclude homogeneous contributions.

In summary, MXenes offer a versatile and chemically dynamic platform for supporting metal atoms, clusters, and NPs in heterogeneous catalysis. Their unique 2D structure, surface terminations, and defect sites, especially M vacancies, not only serve as anchoring points for atomically dispersed metals but also facilitate charge transfer and interface formation. By integrating the comparative understanding of different MXene hosts, the governing principles of metal–support interactions, and rigorous catalytic validation, a more mechanistic and quantitative framework for MXene-supported catalysis can now be established. A range of spectroscopic and microscopic tools such as XAS, XPS, HAADF-STEM, and EDX are essential for elucidating the local bonding environment, oxidation states, and distribution of metal species on MXene surfaces. Furthermore, engineering the MXene support via reductive treatments, surface defunctionalization, or hybridization with oxides can improve metal dispersion and prevent layer restacking, maximizing accessibility and catalytic efficiency. These advances underscore the importance of precise structural and electronic characterization in optimizing MXenes as high-performance catalyst supports.

Building upon these insights, the following subsection (Section 3.3) provides a focused overview of the advanced characterization tools most relevant for identifying, quantifying, and correlating the catalytic active sites in MXenes described in the previous subsections. By bridging experimental techniques with mechanistic understanding, this section establishes a foundation for linking structural descriptors to catalytic performance.

### 3.3 Advanced Characterization Tools for Identifying Catalytic Active Sites in MXenes

As illustrated in previous Figure 27, active sites on MXenes can be either inherent to their structure or introduced through the addition of an extrinsic component. The structural sites can include the most abundant M elements bound to surface terminal groups or structural defects. These defects may consist of anomalous surface terminations or their vacancies, as well as vacancies of the M element or doping/vacancies in the carbide/nitride layer. Peripheral atoms may also exhibit catalytic behavior due to their anomalous termination. In addition to having intrinsic catalytic activity, MXenes possess unique properties as supports for SAs, clusters, and NPs, in which the catalytic activity resides in components that do not form part of the MXene composition. Along with discussing the nature of the catalytic sites, this section also covers characterization techniques used to identify and quantify these centers. These include techniques commonly employed in materials science to determine the structure of solids, as well as catalytic-specific methods used to titrate acid-basic sites or assess the reducibility/oxidizability of materials.

**Figure 27:**
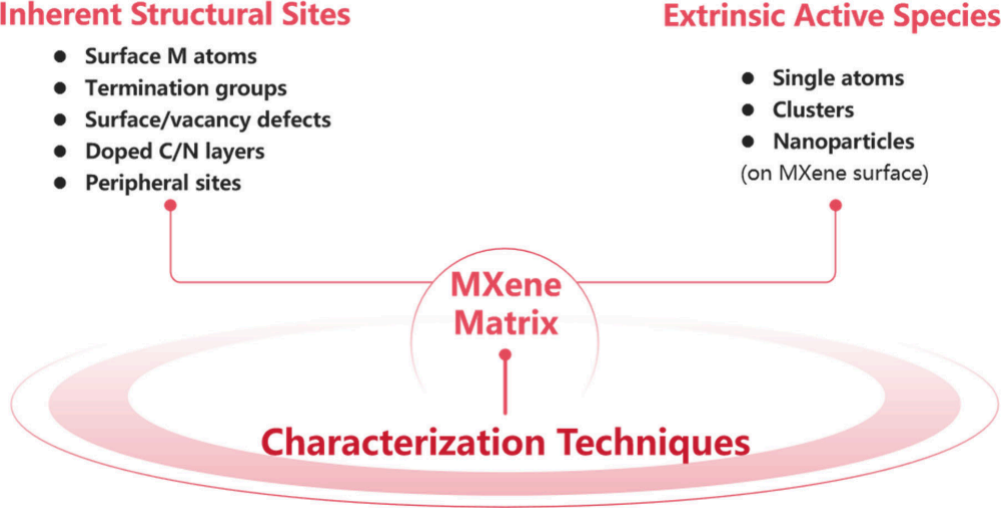
Classification of possible catalytic sites on MXenes depending on whether they form part of the composition of the MXene or not.

As noted in Section 2.1, a general route to obtain MXenes is via selective etching of the corresponding MAX precursors to remove the “A” element. The harsh conditions used in this etching process to eliminate Al layers from MAX phases can sometimes strip away transition metal atoms from the MXene structure, leaving behind single vacancies or multivacancy clusters. These defects are typically unstable and tend to be highly oxyphilic and reactive, leading to the formation of oxide patches via hydrolysis or oxygen chemisorption. The vacancies generated during etching may serve as active sites for catalytic reactions or anchoring points for immobilizing single metal atoms or clusters, which can themselves act as active sites and contribute to efficient catalytic performance.

Understanding the defect structure, the interaction between defects and surface terminations, and the evolution and dynamics of defects under various conditions, such as temperature and time, is essential for the development of MXene-based catalysts with tunable properties. This remains a major challenge and requires the integration of advanced characterization techniques.

#### 3.3.1 Characterization of Acidity and Basicity of MXenes

Acid and basic sites, either Brönsted or Lewis, are a common type of centers that can catalyze a significant number of organic reactions, including electrophilic additions to multiple bonds, eliminations, aldol reactions, condensations, etc.^130^ Pyridine adsorption/desorption monitored by FT-IR spectroscopy is a routine technique commonly used to examine the nature of acidic sites on solid catalysts.^131^ This method can distinguish between Brönsted and Lewis acid sites and can serve to classify the sites as weak, medium, or strong depending on how the pyridine bands decrease in intensity as a function of the desorption temperature. Bands of adsorbed pyridine appearing in FT-IR spectroscopy around 1450 and 1588 cm^–1^ are specific to the interaction of pyridine with Lewis acid sites. Using this technique, it was observed that Nb_2_CT_*x*_ exhibits strong Lewis acid sites, which were attributed to surface defects formed during chemical etching and the unsaturated coordination of partially oxidized Nb^3+^-O and Nb^4+^-O, as well as oxidized Nb_2_O_5_ (Figure 28a).^132^ Brönsted acid sites have also been observed for Nb_2_CT_*x*_ using pyridine as a probe, but the exact structure of these sites is still unveiled.

**Figure 28:**
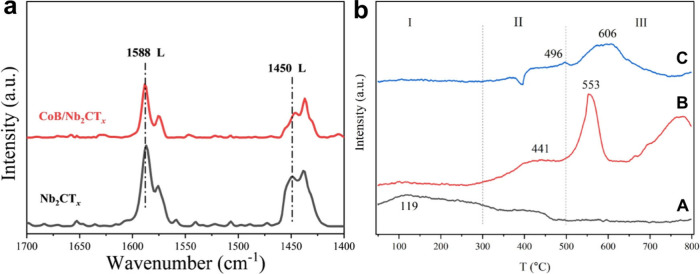
(a) Pyridine FT-IR spectra of Nb_2_CT_*x*_ and CoB/Nb_2_CT_*x*_. (b) NH_3_-TPD profiles of A) CoB, B) Nb_2_CT_*x*_ and C) CoB/Nb_2_CT_*x*_. CoB corresponds to a Co alloy with B formed by reduction of Nb_2_C-adsorbed Co^2+^ ions with KBH_4_. Reproduced with permission from ref. 132. Copyright 2024 Springer.

One of the main problems of the pyridine adsorption/desorption technique for characterizing and quantifying acidity in MXenes is the low IR transmission of these materials, which generally limits the applicability of this spectroscopy. In this regard, NH_3_-TPD is an alternative technique used to determine the strength of acidity and quantify the acid sites in solid materials, the main difference from the pyridine method being that NH_3_-TPD cannot distinguish between Brönsted and Lewis acid sites. In NH_3_-TPD, NH_3_ is adsorbed onto a clean solid at ambient temperature, and then the sample is subjected to gradual heating at a constant rate, quantifying the amount of NH_3_ desorbed as a function of the temperature. Peaks observed between 100 and 300 °C indicate the presence of weak acid sites, those between 300 and 500 °C correspond to moderately strong acid sites, and those between 500 and 700 °C are attributed to strong acid sites. NH_3_-TPD titration of a Nb_2_CT_*x*_ sample has shown two peaks centered at 440 and 553 °C, indicating that this Nb_2_CT_*x*_ sample is dominated by medium and strong Lewis acid sites (Figure 28b).^132^ However, the population of these acid sites is very low, typically on the order of 10 μmol g^–1^, which is 2 to 3 orders of magnitude lower than that of typical solid acids, such as zeolites and aluminosilicates, which ranges from 300 to 1000 mmol g^–1^ depending on the Si/Al ratio of the zeolite.

In heterogeneous catalysis, it is well-known that samples of the same material can exhibit different populations and strength distribution of the acid sites depending on the details of the preparation procedure, and it should be expected that this also occurs on MXenes. Therefore, the presence, population, and strength distribution of acid sites in MXenes should not be taken for granted, and it is necessary to determine the acidity of each sample under study until a better understanding of the acid–base properties of these materials is achieved.

Besides acidity, solid surfaces can also process basic sites. The population and strength of these basic sites can also be determined by TPD, using CO_2_ as the probe molecule for basicity. These measurements have also been performed for Nb_2_CT_*x*_ samples, revealing that the density of basic sites can be higher than that of acid sites, reaching approximately 30 mmol g^–1^. Considering the wide scope of acid–base-catalyzed reactions, it would be important to determine the structure of these acid/basic sites and devise methods to increase their numbers and control their strength, as this would broaden the applicability of MXenes as catalysts for a wide range of reactions. In one reported case, we have claimed that the combination of acid and basic sites in Nb_2_CT_*x*_ is responsible for its high TOF in aldolic condensation.^92^ Solids that simultaneously possess appreciable populations of both acid and basic sites are denoted as “bifunctional” and can exhibit remarkable activity, often exceeding that of materials with exclusively strong acids or bases sites. This is the case of Nb_2_CT_*x*_, which, based on acid–base titration, has been reported to exhibit catalytic activity, as measured by TOF for the aldol condensation of benzaldehyde and furfural, that is higher than that observed for zeolites or MgO under the same conditions.^92^ However, even though the TOF per acid/base site of Nb_2_CT_*x*_ is higher than that of other solids, the low population of such sites still necessitates the development of strategies to enhance performance on a per-mass basis.

Overall, the acidity and basicity of MXenes, which are primarily influenced by surface terminations and defect structures, play a crucial role in their catalytic behavior. While FT-IR spectroscopy with pyridine as probe and NH_3_-TPD have confirmed the presence of medium-to-strong Lewis acid sites, particularly in Nb_2_CT_*x*_, the overall site density remains significantly lower than in conventional solid acids. Similarly, CO_2_-TPD analysis indicates the presence of basic sites with higher density than acid sites, enabling potential bifunctional catalysis. However, the variability in acid–base properties depending on synthesis and treatment conditions underscores the necessity for careful, sample-specific characterization. Strategies to enhance site density and tailor their strength will be essential for unlocking the full catalytic utility of MXenes in acid–base-driven reactions.

#### 3.3.2 Thermoprogrammed Reduction and Oxidation

Besides acidity and basicity, other general types of active sites frequently found in solids are redox centers, typically associated with transition metal elements having various possible oxidation states. This type of sites can be studied by thermoprogrammed reduction and oxidation techniques.^133^ In thermoprogrammed techniques, a known amount of the material is exposed to a stream of a reducing or oxidizing agent, while the temperature is gradually increased at a constant rate. The most common reducing gas is hydrogen (H_2_-TPR), while oxygen is the preferred oxidizing gas (thermoprogrammed O_2_ oxidation, O_2_-TPO). By using thermal conductivity detectors, as those used in gas chromatography, the amount of reducing or oxidizing agent is continuously monitored, and it can be quantified when it decreases due to reaction with the solid. This allows a quantitative measurement of the redox centers in the material per mass unit. In that way, by exposing MXene to a H_2_ stream at increasing temperatures, the onset temperature at which this MXene reacts with H_2_ can be determined, but also what is the total H_2_ consumption per unit mass. This allows to determine the stoichiometry of the reduction process assuming that the metals are the elements responsible for H_2_ consumption. Considering that the current main application of MXenes as solid catalysts is hydrogenation, H_2_-TPR provides valuable experimental information to address the reaction mechanism. Analogously, O_2_-TPO gives important data about MXene stability against oxidation.^36^

#### 3.3.3 Characterization Techniques for Heteroatom Doping

Doping MXenes with heteroatoms is crucial for optimizing their catalytic properties, stability, and selectivity, opening possibilities for advanced applications such as CO_2_ hydrogenation catalysis and others. This doping strategy enables fine-tuning of MXenes for specific reactions by controlling surface chemistry and electron distribution. N-doping of MXenes by treating a MXene sample with NH_3_ flow at high temperature (MXene-NH_3_) can be experimentally confirmed by XPS, monitoring the N 1s core-level spectrum, whose deconvolution indicates the presence of different types of nitrogen atoms (Figure 29).

**Figure 29:**
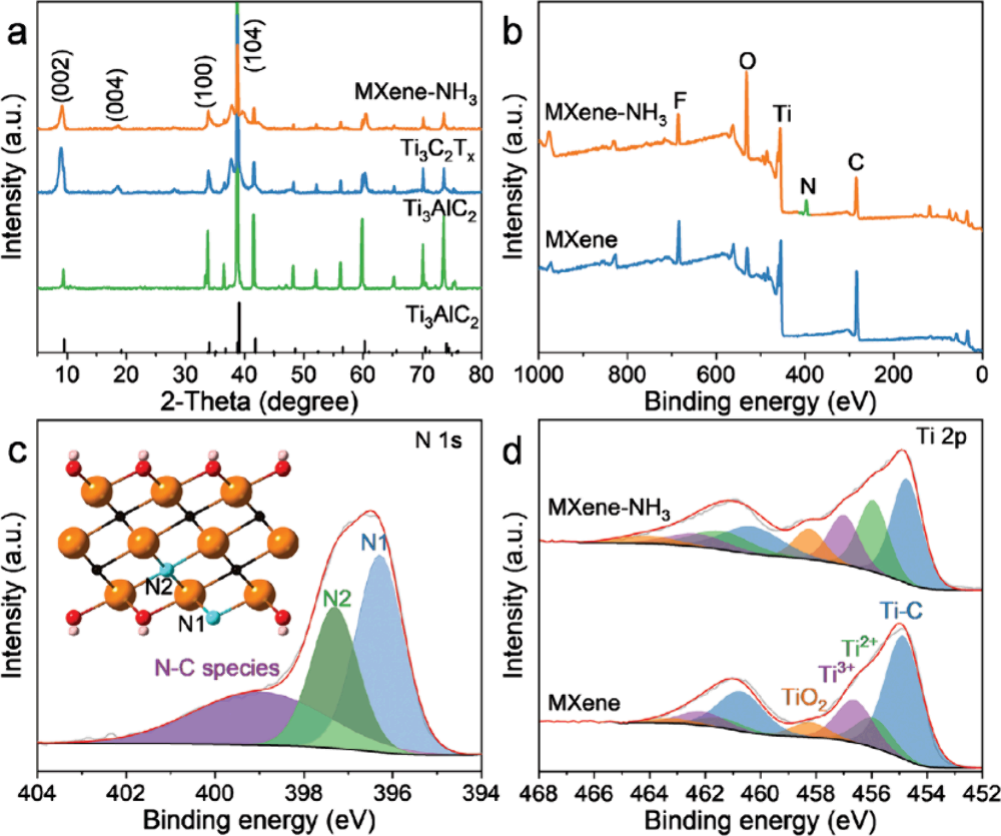
(a) XRD patterns of Ti_3_AlC_2_, Ti_3_C_2_T_*x*_, and MXene-NH_3_; PDF #52-0875 corresponding to Ti_3_AlC_2_. (b) XPS survey for MXene and MXene-NH_3_, (c) high-resolution N 1s spectrum of MXene-NH_3_, and (d) high-resolution Ti 2p spectra of MXene and MXene-NH_3_. Reproduced with permission from ref. 124. Copyright 2021 Wiley.

By altering the electron density distribution, doped heteroatoms change the redox properties of MXenes, making them more effective for reactions that require electron transfer, such as hydrogen evolution or catalytic CO_2_ reduction. For instance, N-doping in Ti_3_C_2_T_*x*_ increases its reducibility, which improves the MXene ability to facilitate redox reactions and interact with metal NPs.^124^ Doping can also introduce or stabilize vacancy sites (e.g., Ti or C vacancies in Ti_3_C_2_T_*x*_), which serve as highly active sites for adsorbing and activating small molecules like CO_2_, H_2_, and N_2_.^106^ These defects increase the interaction with reactants, lower activation barriers, and improve reaction kinetics. By modulating the surface properties of MXenes, doping can steer reaction pathways toward desired products. In a study on Co/MXene, N-doping led to a higher proportion of CH_4_ formation in CO_2_ hydrogenation, whereas undoped MXene as a Co support predominantly formed CO.^124^ This selectivity change results from modified MSIs that affect the adsorption and activation of reaction intermediates, enabling more efficient and selective catalytic processes. Doping introduces additional active sites and modifies the electron density around the metal centers in MXenes, thereby improving their catalytic performance. In the case of Co/MXene-NH_3_, N-doping leads to the formation of surface TiO_2_ and Ti vacancies, which enhance interactions with the Co NPs, facilitating the CO_2_ hydrogenation reaction and shifting the product selectivity from CO to CH_4_.

Heteroatom doping can be intentionally performed before catalytic reactions, or it may occur *in situ* during the reaction, each having distinct implications for MXene performance and stability. Prereaction doping is designed to introduce active sites or to adjust the catalysts electronic structure and surface properties in anticipation of a specific reaction. It typically involves chemical treatments, such as annealing in NH_3_ or nitrogen atmospheres for N-doping, or wet impregnation of dopant precursors followed by reduction or calcination for others. Predoping can create stable active sites that are less prone to deactivation during the reaction, as the catalyst structure is already optimized. For example, as noted above, N-doping in Co-MXene shifts CO_2_ hydrogenation selectivity toward CH_4_ over CO by modifying the interaction between Co sites and the MXene support.

Doping can also occur during the reaction due to exposure to reactants or products that incorporate heteroatoms into the catalyst over time. *In situ* doping can happen when catalysts are exposed to a reaction environment containing dopant atoms (e.g., N or O from gases like NH_3_ or NO_2_) at high temperatures, causing the incorporation of these atoms into the catalyst surface. In some cases, the reaction modifies the catalyst surface in beneficial ways, such as forming active doped sites that were not initially present in the material or exposing fresh active sites that enhance reaction kinetics. For instance, during NH_3_ synthesis using Co supported on Mo_2_CT_*x*_, partial nitridation of the MXene structure occurs, specifically in the Co-decorated Mo_2_CT_*x*_ catalyst variants prepared using Co­(NO_3_)_2_, as the Co source (1-CoNit-Mo_2_CT_*x*_ and 5-CoNit-Mo_2_CT_*x*_). Postreaction analysis indicated a partial replacement of carbon by nitrogen in the MXene lattice, which is critical for achieving enhanced catalytic activity. This nitridation was only observed in the active Co–Mo_2_CT_*x*_ catalysts used in NH_3_ synthesis and not in the nondecorated MXene samples. Nitridation was evidenced by elemental analysis, where the nitrogen content in postreaction samples suggests partial replacement of carbon by nitrogen.^106^ Furthermore, XPS of the used catalysts revealed a new peak at around 397.6 eV in the Mo 3p core-level spectrum, corresponding to Mo–N bonding, with a binding energy consistent with known values in molybdenum nitrides, providing convincing evidence for the nitridation process in the MXene structure.^106^

In summary, heteroatom doping has emerged as a powerful strategy for tailoring the catalytic performance of MXenes by modulating their surface chemistry, electronic structure, and defect profile. Experimental techniques such as XPS and elemental analysis provide direct evidence of successful doping, while postreaction characterizations confirm the dynamic evolution of doped sites under operating conditions. Both prereaction and *in situ* doping approaches can enhance MXene activity, selectivity, and stability by introducing new active sites, strengthening MSIs, or facilitating specific reaction pathways. Particularly, N doping has proven effective in promoting CO_2_ hydrogenation toward CH_4_ formation and enhancing metal dispersion. These findings underscore the crucial role of rational heteroatom engineering in developing next-generation MXene-based catalysts for energy and environmental applications.

Since TON and TOF calculations require the quantitative estimation of the density of active sites, this number must be obtained using those characterization techniques that are most appropriate to provide this quantitative measurement. The selection of the technique depends on the mechanism operating in the reaction that indicates which are the sites to be titrated. For acid–base catalyzed reactions, acid–base titrations are the most suited, while for redox processes, such as hydrogenation and oxidation, H_2_-TPR and O_2_-TPR should be able to quantify the number of sites. When the active sites are defects of metal atoms, CO adsorption measurements appear to be better suited to measure the sites.

## 4 Best Practices in Thermal and Photothermal Catalysis with MXenes

After discussing the active sites present on MXenes, their characterization and quantification, the following paragraphs provide a succinct overview of best practices in catalysis, specifically applied to MXenes as thermal catalysts. Of particular importance is how to assess the relative catalytic activity of MXenes with respect to known benchmark catalysts, as well as the evaluation of their stability under reaction conditions. One key issue in MXene catalysis is the general variability of their catalytic activity depending on the exact preparation conditions, which raises concerns about data reproducibility across different synthesis batches and between laboratories. Data reproducibility is a general challenge in heterogeneous catalysis, but it is especially critical in the case of MXenes, given that the nature and distribution of surface terminations, as well as defect densities, are highly dependent on the specific protocol used in their preparation and manipulation. This is compounded by the challenges already discussed regarding the characterization and quantification of active sites present in the material in low-density. Another specific concern for MXenes is storage and their use as oxidation catalysts. Factors like exposure to ambient oxygen and moisture, temperature of storage and elapsed time since preparation can lead to partial MXene oxidation that can influence the catalytic activity. MXene samples should be preferentially stored under inert atmosphere, in fresh and dry ambient conditions and used shortly after preparation. Under oxidizing conditions, catalytic studies must provide rigorous and careful proof of structural stability. This section of the review also includes a separate discussion focused on the photothermal properties of MXenes. In photothermal processes, thermal reactions are driven by the conversion of photon energy into heat, which requires specific metrics and experimental validation of the light effect beyond simple thermalization. Scheme 4 provides a concise overview of the stability and artifacts that can affect MXene catalysis. As it has been emphasized in Section 2, the preparation procedure determines the nature of the surface terminal groups and the density of defects, and these parameters determine the catalytic activity of a particular MXene batch. In addition, storage and time elapsed since the preparation is another factor to be considered, since oxidation of a MXene sample can occur at longer time scales upon exposure to oxygen and in the presence of moisture. A good practice is to use freshly prepared batches or to ensure that no oxidation has occurred since sample preparation. As indicated in Scheme 4, sonication and dispersion conditions can also affect the lateral size of MXene particles and can increase the relative contribution to the catalytic activity of edges vs. the less reactive basal plane. Edges expose C and N atoms that are highly relevant in certain types of catalysis, particularly those occurring through a Mars–Van Krevelen mechanism, but can also introduce basic sites. Thus, a good practice should indicate the average particle size distribution of the MXene batch used as catalyst, since it can be anticipated that basal and edge catalysis should be very different. Related to the previous factor, another possible artifact can come from the residual presence of intercalant agents used in the exfoliation process. Intercalants such as DMSO or quaternary ammonium salts are known to be very difficult to remove from the MXene sample by washing or other means. They can block active sites and influence the catalytic activity. Solid-state ^1^H and ^13^C NMR spectroscopy techniques should detect these species, and their quantification by elemental analysis should give an estimation of their content. Finally, good practice requires characterization of the MXene sample used in catalysis by the same means as the fresh sample (XRD, XPS, TEM/SAED, EPR for vacancies, ICP for leaching, acidity, basicity, etc.) to assess the possible changes occurring in the composition and the structure of the MXene during catalysis (Scheme 4).

**Scheme 4:**
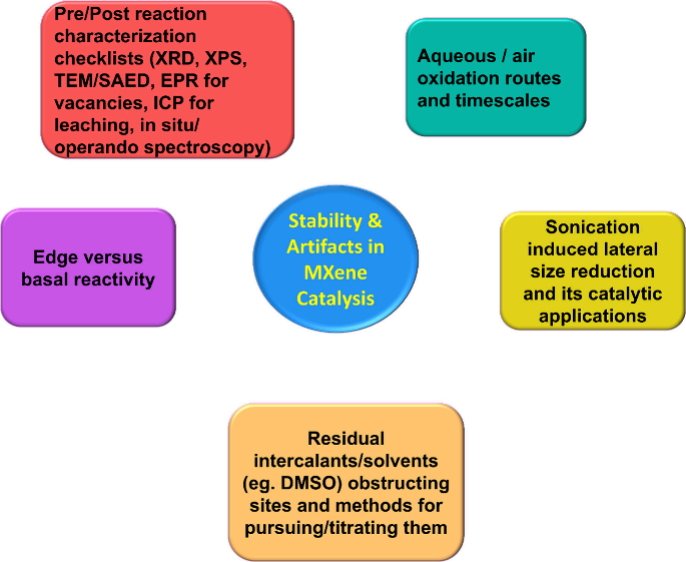
Concise Overview of the Stability and Artifacts in MXene Catalysis

### 4.1 Catalytic Evaluation Protocols: TOF, Conversion, and Stability

Reactions using MXenes as catalysts can be carried out in the gas or liquid phase with substrates and reagents dissolved in a suitable solvent. These processes can be performed in batch or continuous-flow reactors, where parameters such as temperature, pressure, catalyst-to-substrate ratio, and space velocity significantly affect substrate conversion and product selectivity. In liquid-phase reactions, MXenes can be dispersed in the solvent via controlled sonication, with stirring used to maintain a stable suspension. Notably, high-power or prolonged ultrasound treatment may reduce the lateral size of the MXene flakes, and this particle downsizing can positively influence catalytic performance.^80,134,135^

The most reliable figure of merit to compare different catalysts is the TOF-defined as the number of product molecules formed per active site per unit time.^136^ Another useful metric is the TON, representing the average number of catalytic cycles each site undergoes during the reaction time. Both TOF and TON calculations require an estimation of the density of active sites per gram of catalyst.^136^ As discussed in Section 3, even though the exact nature and uniformity of active sites may vary, certain types of sites—such as acid, basic, or metal-based—can be quantitatively determined. For example, catalytic activity can often be correlated with acid site density, as it is known from other catalytic systems. This allows for estimating active site densities through NH_3_ or pyridine titration (Section 3.3.1), and, in turn, calculating TOF values, assuming that the reaction is indeed catalyzed by those specific sites. In the hydroamination of terminal alkynes and the guanylation of amines catalyzed by Ti_3_C_2_, a linear correlation has been observed between catalytic activity and the density of acid sites, as measured by NH_3_ titration.^80^ TOF values were calculated by dividing the moles of product by the moles of acid sites and time.^80^ As shown in Figure 30, a linear dependence exists between the initial reaction rate for the guanylation of *p*-toluidine and the density of weak acid sites titrated by NH_3_, while no such correlation is observed for strong acid sites. This correlation has allowed to rank Ti_3_C_2_ as one of the most efficient catalysts reported for amine addition reactions.^80^ The true TOF value may even be higher, considering that NH_3_ titration can detect sterically hindered acid sites that bulkier amine substrates cannot access.

**Figure 30:**
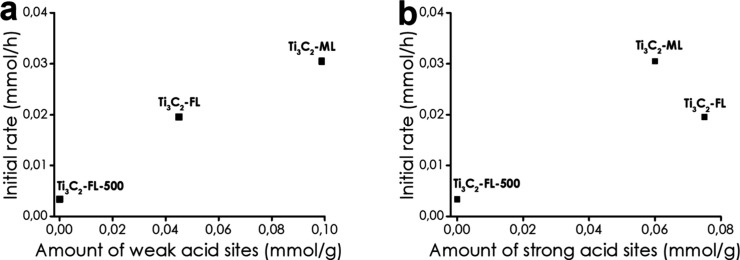
(a) Plot of the initial rate vs the amount of weak acid sites of Ti_3_C_2_-ML, Ti_3_C_2_-FL and Ti_3_C_2_-FL-500. (b) Plot of initial rate-amount of strong acid sites of Ti_3_C_2_-ML, Ti_3_C_2_-FL and Ti_3_C_2_-FL-500. ML and FL refer to multilayer and few layers, respectively. FL-500 corresponds to a few layers Ti_3_C_2_ sample treated at 500 °C. Reaction conditions: 7.5 mg of catalyst, p-toluidine (0.25 mmol), *N*,*N*′-diisopropylcarbodiimide (0.35 mmol), 130 °C. Reproduced with permission from ref. 80. Copyright 2025 Elsevier.

Since the initial rate represents the highest rate in a process lacking an induction period and subsequently decreases until equilibrium is reached, it is convenient to determine the TOF values based on these initial rates. However, to avoid uncertainty and increase the accuracy of the measurement, a certain degree of conversion is advisible. A good practice is to report TOF values and the conversion at which they were estimated. Depending on the analytical procedure, conversions between 5 and 10% are adequate, unless there are specific reasons for estimating turnover at lower conversions. In fact, although high conversions are always the goal in a catalytic process, providing data under operating conditions in which only moderate conversions are reached can better reflect the relative catalytic activity of various materials. This is because a better catalyst still has the potential to achieve higher conversion, which would not be observed if the operating conditions of less active materials already allow them to reach full conversion.

Stability of active sites is another important issue in catalysis. In batch reactions, catalyst stability can be ascertained by using the same catalyst sample in several consecutive reaction runs and observing whether the temporal profiles of substrate conversion and product evolution remain unchanged. A typical protocol consists of recovering the solid catalyst, washing it with fresh solvent, drying it at ambient or moderate temperature, and reusing it. For complete catalyst recovery, filtration or centrifugation are commonly employed. Identical time–conversion plots confirm that the reused sample retains both the initial reaction rate and the final conversion and yield. Reporting only the final yield at a given time is not sufficient to demonstrate catalyst stability upon reuse, because the rate at which this yield was reached, particularly the initial rate, is not provided. In comparison, continuous flow reactions are better suited for assessing catalyst stability, as they allow for monitoring conversion and product selectivity over extended time-on-stream periods. After prolonged catalytic use, the MXene sample should be characterized using the same techniques applied to characterize the fresh material, trying to determine any change in composition or structure. This is especially important for MXenes, as it must be confirmed that the carbide or nitride structure has been preserved and that there is negligible amorphization or transformation into other phases. Surface functional groups likely undergo changes during the reaction, and these surface terminations should also be recharacterized for spent MXene catalysts.

All in all, accurate evaluation of MXenes as thermal catalysts requires standardized protocols that account for their structural complexity and sensitivity to preparation conditions. Determination of TOF based on initial reaction rates at low conversions (typically 5–10%) offers a robust metric for comparing catalytic activities across different systems. However, the uncertainty about the nature of the active sites complicates the wide acceptance of these data. Stability testing, both in batch and continuous flow setups, is essential to assess catalyst durability and reusability, with time–conversion profiles providing more reliable insights than end point yields alone. Postreaction characterization of reused MXenes is crucial to verify structure preservation, particularly under oxidative or reductive conditions that may alter surface terminations or induce amorphization. These best practices ensure reproducibility, accurate performance benchmarking, and deeper mechanistic understanding of MXene-based catalysts.

### 4.2 Photothermal Reaction Metrics

Photothermal reactions convert the energy of light into heat at the point in which the photon is absorbed.^137^ These localized hot spots can be used to promote thermal catalysis, using light instead of conventional heat as the energy source for the reaction. In this way, the local temperature at the reaction site, where photons are thermalized, can be much higher than in other parts of the material or even higher than the overall reactor temperature. Although the photothermal reaction proceeds mainly through the same mechanism as the thermal reaction, assistance of hot electrons or charge separation can accelerate the process.^138,139^ The optical spectrum of the material indicates which wavelengths are absorbed by the solid and which are transmitted, scattered or reflected, together with the corresponding absorption coefficients for each wavelength. This optical absorption spectrum in the UV–Vis-NIR range can be recorded in transmission mode for soluble compounds, in suspension for colloidal solids, or in diffuse reflectance mode for powders. MXenes typically exhibit an absorption band in the UV region corresponding to ligand-to-metal M–O electronic transition, together with broad absorption in the visible range reflecting their metallic nature.^55^ Metallic MXenes also often exhibit a plasmonic band in the red region of the visible spectrum, attributed to the lateral confinement of free electrons in the 2D structure, similar to the behavior observed in metal NPs.^140,141^

Light absorption across the full spectral range is responsible for the black visual appearance of MXenes, both as powders and inks. In addition, this broadband absorption, together with the metallic character and high lateral thermal conductivity, places MXenes among the best materials for converting light into heat.^84^ The ultrafast hot electron–phonon coupling enables rapid relaxation of electron energy into lattice vibrations, resulting in sunlight-to-heat conversion efficiencies that often exceed 90%. The low heat capacity characteristic of most MXenes and metals causes rapid temperature rises upon illumination, reaching values above 300 °C under one-sun power conditions. Figure 31 shows one example of the use of MXene for solar-driven steam generation.^142^

**Figure 31:**
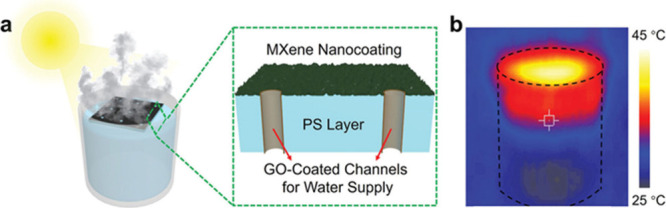
Enhanced solar–thermal conversion for efficient solar steam generation. (a) Schematic illustration of the solar steam-generation device with MXene nanocoating for high solar–thermal conversion. (b) Infrared image demonstrates the temperature distribution of the steam-generation device floating on the water bath in a beaker after 30 min one-sun illumination equivalent to 1.0 kW m^–2^. The black dashed lines represent the approximate edges of the beaker. Reproduced with permission from ref. 142. Copyright 2019 Wiley.

In this regard, MXenes share their solar thermalizing capability with conventional 3D bulk transition metal carbides, with TiC being one of the preferred materials for such applications.^143^ The high stability of MXenes under intense light irradiation and their ability to withstand high temperatures in the absence of oxygen contribute to their excellent photothermal performance over extended operation periods.

In sum, MXenes demonstrate strong potential in photothermal catalysis due to their ability to absorb a wide range of light wavelengths and convert photon energy into localized heat. Their electronic structure, coupled with efficient energy dissipation pathways, supports rapid thermal response under illumination. This enables reaction temperatures suitable for catalytic transformations without external heating. Importantly, their structural robustness under light exposure allows for sustained operation, highlighting their applicability in solar-driven thermal processes.

### 4.3 Guidelines on Experiment Reproducibility and MXene Degradation Tests

It has been found that the catalytic activity of a given MXene depends considerably on the exact preparation procedure and the way the sample has been treated.^94^ This is not surprising, considering that catalytic activity is influenced by surface functional groups and defects, whose exact distribution and nature are difficult to control exactly. This introduces a certain degree of uncertainty in the reproducibility of experimental data, which must be minimized by providing detailed information on the preparation, separation, and treatments of the MXene samples used. Details such as the procedure for adding the MAX precursor to the etching solution, the addition time, etching duration, sonication power, mode, and time can all influence the generation of defects and the surface composition. Comparing activity data from different independently prepared batches that are intended to exhibit identical performance is essential for confirming the reproducibility of catalytic behavior. This is particularly important in the case of MXenes, since as discussed earlier, defects, vacancies, and dopants may serve as the actual active sites in catalysis. Due to their generally low density, such centers are difficult to characterize and quantify, and their generation typically occurs in a stochastic manner.

After exhaustive catalytic use, MXene samples should be subjected to the same characterization techniques as those used for fresh samples, in order to identify changes and understand possible causes of catalyst deactivation. Blank experiments in the absence of one of the substrates should demonstrate the stability of the MXene under reaction conditions in the presence of other reagents over extended periods. These control tests are particularly important when MXenes are exposed to potential oxidizing agents. As with any solid catalyst, hot filtration tests, in which the MXene is removed at the reaction temperature after reaching a certain conversion level and the reaction is allowed to proceed without the solid, provide strong evidence for the heterogeneous nature of the catalysis.^144^ Furthermore, analysis of the clear solution should confirm the absence or quantify the amount of leached “M” metal. If any M metal is detected, even in small amounts, it is advisible to perform a control reaction using a soluble salt of M at a concentration equal to or slightly higher than that measured in the leaching test. This helps determine the extent to which dissolved M species contribute to the observed catalytic activity.

Ensuring reproducibility and structural integrity is particularly critical in MXene-based catalysis due to the sensitivity of their activity to preparation methods, defect distribution, and surface chemistry. Accurate reporting of synthetic parameters, systematic batch-to-batch comparison, and postreaction characterization are essential to validate catalytic performance and stability. Furthermore, rigorous control experiments, including hot filtration, leaching analysis, and blank tests, are necessary to confirm the heterogeneous nature of the catalysis and to exclude contributions from dissolved species. These practices are indispensable for the reliable evaluation and future advancement of MXenes in thermal and photothermal catalytic applications.

### 4.4 Challenges of MXene Stability under Thermal/Oxidative Conditions

The fact that MXenes are obtained from their corresponding MAX precursors, which are prepared by metallurgic synthesis at temperatures of about 1500 °C, suggests a high degree of thermal stability. Accordingly, the thermal stability of MXenes is often taken for granted. Specific studies of their thermal stability under inert conditions have shown that, beyond the removal of some surface terminations, the MXene structure is maintained up to approximately 700 °C.^145,146^ At higher temperatures, a phase transition to bulk TiC has been observed.^146^

However, thermal stability must always be assessed in relation to the surrounding environment. Heating under air typically results in the complete oxidation of MXenes to the corresponding transition metal oxides. Similar oxidation occurs under hydrothermal conditions, and the process is further promoted by increased ionic strength from added salts.^147^ Even boiling MXene suspensions in aerated organic solvents can result in their full transformation into metal oxides.^94^

This inherent tendency of MXenes to form metal oxides presents a significant limitation for their use in oxidation catalysis.^94^ Nevertheless, as the field evolves, strategies to enhance their oxidative stability are expected to emerge. These may include identifying MXene compositions with greater resistance to oxidation, increasing the number of layers, and engineering more robust surface terminations.^36^ Such developments would help broaden the applicability of MXenes in oxidative catalytic reactions.

Although MXenes exhibit excellent thermal stability under inert conditions, their structural integrity is significantly compromised under oxidative or hydrothermal environments, where they readily convert into corresponding metal oxides. This inherent instability currently limits their applicability in oxidation catalysis. Addressing this challenge requires a deeper understanding of the oxidation mechanisms and the development of stabilization strategies such as surface termination control, layer number optimization, and compositional tuning, in order to extend the use of MXenes in thermally and chemically harsh catalytic systems.

All in all, a comprehensive framework for evaluating MXenes in thermal and photothermal catalysis is established, underscoring the necessity of standardized metrics such as TOF, conversion rates, and recyclability tests to assess catalytic activity. In photothermal systems, careful optical characterization and quantification of photon-to-heat conversion are essential to isolate true photothermal effects from mere thermal contributions. Given the strong dependence of catalytic behavior on surface terminations, structural defects, and preparation protocols, detailed reporting of synthesis and treatment parameters is crucial to ensure reproducibility of catalytic activity. Challenges associated with MXene degradation under thermal and oxidative environments have also been discussed, highlighting current material limitations and pointing toward potential strategies for improving structural robustness. Together, these considerations offer a set of best practices for advancing reliable and reproducible MXene-based research in catalysis.

## 5 Catalysis by MXenes: Mechanism-Oriented Classification

Although MXenes have been shown to be among the best-performing electrocatalysts^52,53^ for reactions of much current interest, including hydrogen and oxygen evolution reactions and electrochemical reductions, and are increasingly used as photocatalysts,^55^ their application in thermal catalysis remains considerably less explored.^22^ As discussed in previous sections, MXenes can possess intrinsic structural active sites associated with M-T functional groups, bare M metal atoms, X dopants, atom vacancies, and other defects. Beyond their structural roles, MXenes also offer significant potential as solid Lewis acids and heterogeneous catalysts for hydrogenation reactions. MXenes have also been used in oxidation catalysis, either in the absence of supported metals or with these sites. However, as it has been emphasized and due to the tendency of carbides to undergo oxidation to oxides, caution must be taken in this type of reactions, since a limited stability has always to be considered, providing convincing evidence of the MXene stability and advancing reasons why MXene oxidation does not occur. This tendency to undergo oxidation also applies during sample storage, prior to the use of MXenes in catalysis, as already indicated in Section 4.

### 5.1 Acid/Base Site Catalysis: Origin from Surface Terminations

Two-dimensional materials like MXenes have increasingly been employed as heterogeneous catalysts in recent years.^180^ The most widely studied MXene, Ti_3_C_2_T_*x*_, has been found to exhibit acidic character, presumably derived from surface acid functionalities such as −OH and surface T group vacancies. In a recent study, the catalytic activity of Ti_3_C_2_T_*x*_ and its modified forms was evaluated in the ring-opening of styrene oxide by alcohols, a benchmark reaction to assess the density of acid–base sites.^148^ In general, the catalytic data indicated that both activity and selectivity are highly influenced by surface modification of the MXene, meaning that in this way the acid and basic sites can be altered, thereby allowing to establish a structure–activity correlation. It is believed that MXene contains strong acid sites (both Lewis and Brønsted), which are responsible for the ring-opening and accompanying isomerization reactions, while oxidized form of MXenes, such as MXene-TiO_2_ composite, possesses weaker acid sites that mainly promote ring-opening with high selectivity to the mono O-alkylated product. Through appropriate surface oxidation, the nature and density of acid sites can be controlled, thereby improving the yield of the mono-O-alkylated product to over 80% (Scheme 5). Characterization data indicate that the formation of a thin oxide layer on the surface of Ti_3_C_2_T_*x*_ (Scheme 4) is essential for promoting the ring-opening of styrene oxide through a S_N_1-type mechanism consistent with acid site catalysis. In S_N_1 mechanism acid sites interact with the oxygen atom in epoxide, increasing the positive charge density of the most substituted carbon of the epoxide ring. This leads to the preferential alcohol attack at the most substituted carbon position. Interestingly, the performance of Ti_3_C_2_T_*x*_ (TOF: 55 h^–1^) was superior to the benchmark solid catalyst, Ti-MCM-41 showing a TOF value of 29 h^–1^ (Table 2). It is worth noting that ring-opening reactions are of considerable synthetic importance and have wide industrial applications, particularly in the production of polyols and other bulk chemicals.

**Table 2:** Performance Data of Reactions Being Catalyzed by MXenes in Comparison with a Benchmark Catalyst

MXene	Reaction	Site quantification method	TOF/TON	Benchmark material	Catalyst stability	Ref
Ti_3_C_2_T_*x*_	Ring-opening of styrene oxide	Pyridine DRIFTS	55 h^–1^	Ti-MCM-41 afforded 29 h^–1^	NR	^148^
Nb_2_C	Aldolic condensation	NH_3_- and CO_2_-TPD	855 h^–1^	Comparable to MgO or HZSM-5	Five uses, TEM and XPS	^92^
Ti_3_C_2_	Guanylation of carbodiimides	NH_3_-TPD	114 h^–1^	Cu/Graphene gives a TOF of 17 h^–1^ (calculated from ref. 149)	Four uses	^80^
Ti_3_C_2_T_*z*_	Hydrogenation of furfural	DFT	145 mmol_furfural_ g_catalyst_^–1^ h^–1^	Pd-Ir(Pd:Ir 1)/SiO_2_ 1.1 × 10^–4^ h^–1^ from ref. 150	XRD, XPS	^95^
Ta_2_C	Electrocatalytic reduction of 4-nitrophenol	NR	NR		NR	^151^
Ti_3_AlC_2_ MAX	ODH of butane	DFT	NR	VO_2_-hexagonal mesoporous silica 28 h^–1^ at 540 °C from ref. 152	NR	^111^
Ti_2_CT_*x*_	ODH of *n*-butane	EPR	31 mmol C_4_·Ti atoms^–1^·min^–1^		NR	^108^
Ti_2_CT_*x*_	Propane dehydrogenation	First-principles calculations	NR		NR	^109^
0.1Pt/PMX	Semihydrogenation of butadiene	STEM-ADF	-		Post reaction STEM On-stream stability (12–24 h)	^128^
0.5 wt.% Pd/Nb_2_C	Semihydrogenation of phenylacetylene	DFT	10372 h^–1^	Pd/Al_2_O_3_ at 50 °C, 1 bar, 96% selectivity to styrene 3960 h^–1^ from ref. 153	Six uses, TEM	^126^
H_2_-Pd/Nb_2_C	Semihydrogenation of phenylacetylene	TEM, ICP	7263 h^–1^	H_2_-Pd/Nb_2_C demonstrated a 15-fold higher TOF value than a Lindlar catalyst	Five cycles	^154^
RuNCs/Ti_3_C_2_T_*x*_-3	Hydrogenation of quinoline	TEM, ICP	7.8 h^–1^ ^a^	Pd NPs on amine-rich mesoporous silica hollow nanospheres 5052 h^–1^ taken from ref. 155	Six recycles, post reaction XRD	^156^
Pt/Ti_3_C_2_T_*x*_D-AB	Hydrogenation of 4-chloronitrobenzene	ICP, chemisorption	4.95 × 10^4^ h^–1^ ^a^	Ni SAs supported on N-doped carbon 8.4 h^–1^ from ref. 157	Six cycles, XRD, TEM	^158^
Ag/r-Ti_3_C_2_T_*x*_	Reduction of 4-nitrophenol	ICP, TEM	1109 h^–1^	Cu_3_(PO_4_)_2_ 1091.6 h^–1^ from ref. 159	Five cycles, XRD	^160^
Rh_2_@V_2_CO_2_	Ethane dehydrogenation	First -principles calculation	NR		NR	^161^
RhNi/Ti_3_C_2_	Decomposition of hydrazine hydrate	TEM, ICP	857 h^–1^	Rh_34_Ni_66_@ZIF-8 140 h^–1^ from ref. 162	Six cycles	^163^
Ti_3_C_2_@PrF_3_-1%	Dehydrogenation of AlH_3_	NR	NR	H_2_O can decompose AlH_3_	XPS	^164^
Pt/MXene-H_2_O_2_	Hydrolysis of ammonia borane (AB)	TEM	272 min^–1^	PtNi@TiO_2_ 1055.2 mol_H2_ mol_Pt_^–1^ min^–1^ from ref. 165	NR	^166^
Ru/TASC-NaOH	Hydrolysis of AB	ICP	582 min^–1^		Six cycles	^167^
Pd_0.7_Cr_0.3_/NH_2_-MXene	Formic acid dehydrogenation	TEM, ICP	1906 h^–1^	Pd supported on amino sepiliolite (pH 8.6) 5587 h^–1^ from ref. 168	Five cycles, TEM	^169^
Pt/Mo_2_TiC_2_T_*x*_	Dehydrogenation of ethane and propane	Pt dispersion; H_2_ and CO chemisorption	1.2 s^–1^	PtIn-SiO_2_	Maintained activity for 24 h on stream	^125^
Bi@V_2_CO_2_	CO_2_ Hydrogenation	DFT calculations	-	Bi catalysts mostly used in electrocatalysis (see ref. 170)	NR	^171^
Ni@Ti_3_C_2_O_2_	CO_2_ Hydrogenation	First-principles calculations	-	Ni catalysts mostly for CO_2_ hydrogenation to CH_4_ (see ref. 172)	NR	^173^
Mo_2_TiC_2_O_*x*_	CO_2_ adsorption	DFT calculations	-		NR	^174^
Cu/Mo_2_CT_*x*_/SiO_2_	CO_2_ Hydrogenation			Cu-ZnO/Al_2_O_3_ 5 or 0.5 s^–1^ at 0.01 or 0.02% conversion from ref. 175	Time on stream (TOS) of more than 20 h; TOS of 100 h shows a stable methanol steady-state value (STY)	^129^
Co/MXene-NH_3_	CO_2_ Hydrogenation			Co/CeO_2_ 2 min^–1^ toward CH_4_ from ref. 176	XRD, XPS	^124^
Pt/Ti_3–*x*_C_2_T_*y*_	N-Formylation of aniline with CO_2_	STEM, ICP		Pd–Au/carbon nanotubes 2.6 h^–1^ calculated from ref. 177	Five uses	^113^
2%Pt/Ti_3_C_2_-R	Oxidation of formaldehyde	ICP, DFT calculations	60 mmol_FA_ mol o^–1^ s^–1^	Pt 1%/MgO 2.87 s^–1^ from ref. 178	TOS of 50 h	^179^
Co/Ti_2‑x_N	Degradation of pollutants				Four cycles	^114^
Cu-SA/Ti_3_C_2_T_*x*_-900	Degradation of bisphenol-A	ICP	4.71 × 10^–2^ min^–1^			^120^

a Calculated from the reported activity data. NR stands for not reported.

**Scheme 5:**
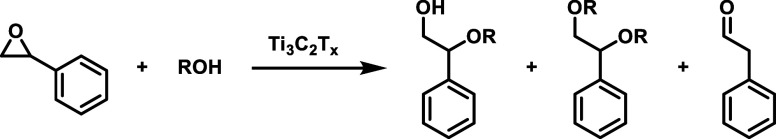
Ring Opening of Styrene Oxide Catalyzed by MXene-Based Solid Catalyst

As discussed earlier, acidity can be measured by NH_3_-TPD and pyridine adsorption/desorption, while basicity can be estimated by CO_2_-TPD. The simultaneous presence of both acid and basic sites has been proposed as the origin of the remarkable catalytic activity of Nb_2_C in aldol condensations (Scheme 6), a general reaction for C–C bond formation from aldehydes and ketones.^92^ In bifunctional acid–base catalysis, the acid sites activate one of the aldehydes, while the basic sites interact with the acidic alpha hydrogen assisting enolate formation. In this way, even if acid or basic sites are weak, the double activation for each substrate results in an efficient catalyst. This is apparently the case of Nb_2_C where no strong acid or basic sites are present. In this case, TOF values were estimated from quantification of acid sites, which are on the order of ∼ 10 μmol g^–1^ for the samples studied,^92^ 1 to 2 orders of magnitude lower than those of conventional solid acids. However, the TOF value of Nb_2_C ranks it as more active per site than typical solid acids (e.g., zeolites) or bases (e.g., MgO), with the enhanced activity attributed to the bifunctional acid/base nature of Nb_2_C (Table 2).^92^ Nonetheless, increasing the specific activity per unit mass remains a key challenge, requiring deeper understanding of the site structure and strategies to enhance their density. This can be achieved by establishing structure–activity correlation that should provide information on the structure of the acid and basic sites and tools to tune their strength.

**Scheme 6:**
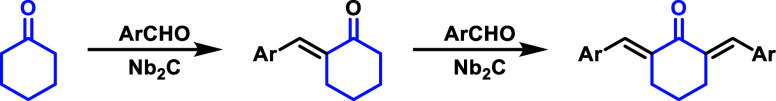
Aldolic Condensation of Cyclohexanone with Benzaldehyde

The mild acidity of Ti_3_C_2_ also appears suitable for promoting aminations of multiple bonds, such as the hydroamination of terminal alkynes (1-hexyne) by aliphatic amine (n-butylamine) (see Scheme 7) and aromatic amines^99^ and the guanylation of carbodiimides^80^ (Scheme 3). In these reactions, excessively strong acid sites would bind too tightly to the amine through its lone pair, blocking the active site and inactivating the amine. For this reason, sulfuric acid and other strong liquid acids are not suitable catalysts for hydroamination reactions. In both transformations, high TOFs, comparable to or better than benchmark homogeneous or heterogeneous catalysts often exceeding 114 h^–1^, have been reported. This TOF value is superior compared to Cu/Graphene solid, showing a TOF value of 17 h^–1^ (Table 2). A linear relationship between initial reaction rates and the density of weak acid sites supports the conclusion that these moderate acid sites are catalytically relevant ones. In heterogeneous catalysis using solid acids, a linear correlation between initial reaction rate and the total population of acid sites of a given strength is considered as convincing experimental evidence of the type of site participating in the reaction mechanism. The process is highly sensitive to steric hindrance in the transition state, which explains the poor reactivity of internal alkynes and the regioselectivity toward the anti-Markovnikov product.

**Scheme 7:**
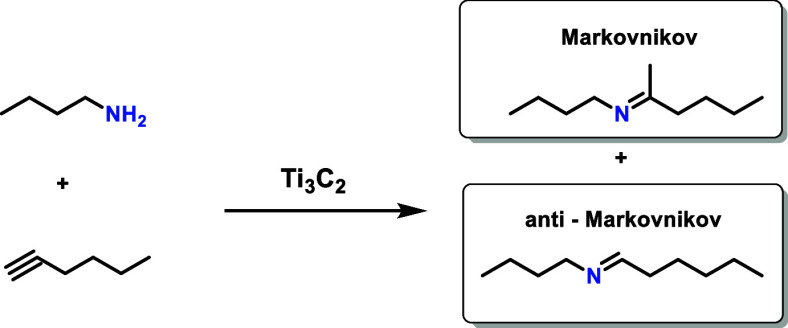
Hydroamination of 1-Hexyne with n-Butylamine Catalyzed by Ti_3_C_2_ with High TOF Values in the Range from 300 to 100 h^–1^

Naguib and co-workers have reported the synthesis, characterization and catalytic performance of Ti-based carbide and carbonitride Ti_3_C_2_T_*z*_ and Ti_3_CNT_*z*_ MXenes in the hydrogenation of furfural using H_2_ or 2-propanol as reducing agents.^95^ Under the optimized reaction conditions, Ti_3_C_2_T_*z*_ and Ti_3_CNT_*z*_ showed 145 and 126 mmol_furfural_ g_catalyst_^–1^ h^–1^, respectively with H_2_ as the reducing agent. This performance is significantly higher than a TOF value achieved with Pd-Ir­(Pd:Ir)/SiO_2_ 1.1 × 10^–4^ h^–1^ (Table 2). On the other hand, 88 and 72 mmol_furfural_ g_catalyst_^–1^ h^–1^ TOFs were observed using Ti_3_C_2_T_*z*_ and Ti_3_CNT_*z*_ as catalysts, respectively with 2-propanol as reducing agent. In all cases, the major product was furfuryl alcohol with a selectivity between 50 and 40%, being the highest with Ti_3_C_2_T_*z*_ and H_2_ as reagent. The other main product observed was 2-methylfuran. Reusability tests indicated that Ti_3_CNT_*z*_ shows better stability than Ti_3_C_2_T_*z*_ (Figure 32). Powder XRD of the Ti_3_C_2_T_*z*_ shows deactivation and is due to the intercalation of reaction products in MXene (Scheme 4). Ab initio calculations indicate that metal and Bronsted acid sites on the MXene surface can heterolytically dissociate H_2_ and favor proton and hydride transfer pathways to promote selective hydrogenation. This mechanistic proposal is reminiscent that frustrated Lewis acid–base pairs known to operate in organocatalysis, in which the bare M atom having high electron density is acting as basic site. With this understanding, higher activity could be achieved upon appropriate generation of surface group vacancies to expose M atoms and by introduction of acid sites.

**Figure 32:**
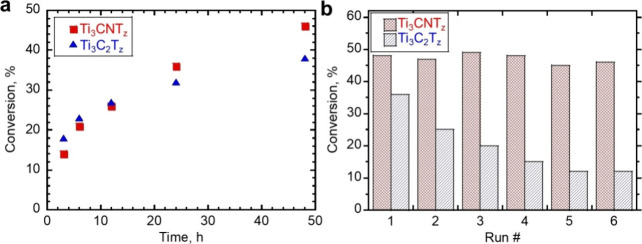
Catalytic performance of Ti_3_C_2_T_*z*_ and Ti_3_CNT_*z*_ a) reaction profiles, and b) stability test. Reproduced with permission from ref. 95. Copyright 2020 Wiley.

Besides etching with fluoride reagents, MXenes can also be obtained by strong alkaline etching of the MAX precursor. This procedure has consequences in the surface functionalization that should favor preferentially −OH groups with an impact on catalytic activity. In a recent example, 2D tantalum carbide MXene was synthesized through the fluorine-free etching method using KOH as etchant. The resulting Ta_2_C exhibits catalytic performance in the reduction of 4-nitrophenol by sodium borohydride (SB) as a reducing agent.^151^ In conditions of a large excess of SB typical for determining maximum reaction rates, Ta_2_C MXene showed pseudo-first-order kinetics, and the rate constant can be used as a quantitative metric to rank the catalytic activity of Ta_2_C among the best performing catalysts. Ta_2_C exhibited complete reduction of 4-nitrophenol after 17 min, 2,4-dinitrophenol after 25 min and 2,4,6-trinitrophenol after 36 min.^151^ This example indicates again the role of surface terminations, Ta–OH in this case, to promote hydride reduction reactions in the absence of metal NPs through a mechanism that probably involves the heterolytic cleavage of the B–H bond. It would have been important to correlate the catalytic activity of these Ta_2_C samples with the density of Ta–OH groups to provide some experimental evidence that these groups are acting as catalytic sites.

In sum, MXenes exhibit promising acid–base catalytic behavior primarily originating from surface terminations (such as −OH) and defect-induced active sites. Ti_3_C_2_T_*x*_, with its moderate acidity, effectively catalyzes reactions like styrene oxide ring-opening and hydroamination of terminal alkynes, where strong acid sites would generate byproducts or would hinder activity by overadsorbing amines. Nb_2_C, on the other hand, benefits from the coexistence of acid and base sites, showing high TOFs in aldol condensation reactions. The intrinsic activity per site often exceeds that of conventional catalysts such as zeolites and MgO. However, the overall catalytic performance remains limited by the relatively low density of active sites. Therefore, strategies that enhance site density while preserving the favorable bifunctionality of MXenes will be critical for advancing their application in thermally driven transformations.

### 5.2 Surface Defects as Active Centers

As previously noted, MAX precursors can exhibit catalytic activity due to surface defects. Among the various reaction types catalyzed by MAX phases, ODH is particularly relevant, as it presents one of the most important petrochemical processes and is typically carried out using metal oxides with oxygen vacancies.^111^ In this context, Ti_3_AlC_2_ MAX phase has been reported as a heterogeneous solid catalyst for the ODH of butane (Scheme 8).^111^ The observed catalytic data revealed that Ti_3_AlC_2_ afforded 35% selectivity toward butenes and 25% selectivity toward butadiene at 10% conversion, with an O_2_/butane ratio of 0.25:1 at 550 °C (Table 3). Notably, the butane conversion increased to 20% and 24%, respectively, at higher O_2_/butane ratios of 0.5 and 1, without significant loss in selectively for butenes and butadiene. The catalyst exhibited good stability, maintaining product selectivity over extended testing. Interestingly, XRD analysis of the spent solid showed no major structural changes, confirming the phase stability (Scheme 4). Although the Ti_3_AlC_2_ MAX phase ideally lacks lattice or structural oxygen, its unique combination of defects and a very thin, presumably nonstoichiometric, oxide surface layer covering the MAX particles, which contains oxygen vacancies, gives rise to O-containing active sites responsible for the catalytic activity observed in this reaction.

**Scheme 8:**
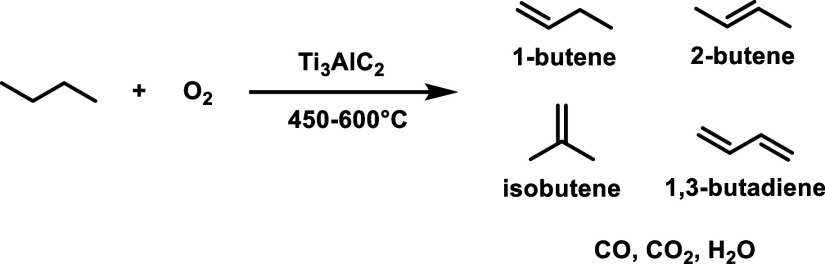
Catalytic ODH of Butane to Give Butenes, 1,3-Butadiene, CO, and CO_2_ ^a^

a This reaction can be promoted by Ti_3_AlC_2_.

**Table 3:** Performance of the Ti_3_AlC_2_ in Butane ODH^111^

O_2_/butane molar ratio^a^	Butane conversion [%]	Total selectivity of butenes [%]	Selectivity of 1,3-butadiene [%]	Selectivity of propene [%]^b^
0.25:1	10.1	35.0	25.0	1.2
0.5:1	20.3	29.0	21.0	1.4
1:1	24.2	27.0	19.5	1.7
1:1^c^	13.8	20.7	16	1.6

a Reaction conditions: temperature = 550 °C; flow rate= 17 mL min^–1^; catalyst = 0.1 g; total pressure= 1 bar.

b Trace amounts of ethylene and CH_4_ were also produced. The remainder are CO and CO_2_.

c Temperature = 500 °C.

The same research group has also exploited structural vacancies in MXenes as active centers to promote ODH reactions. In this context, the performance of Ti_2_CT_x_ MXene was compared with commercial TiC and TiO_2_ in the ODH of *n*-butane.^108^ XPS and Raman spectroscopy revealed that the as-prepared Ti_2_CT_*x*_ MXene contains surface TiO_2_ in both anatase and rutile forms, resulting from partial oxidation during synthesis. The presence of structural defects (i.e., unpaired electrons) within the Ti_2_CT_*x*_ structure was ascertained by X-band EPR spectroscopy. Among the catalysts tested, Ti_2_CT_*x*_ MXene exhibited the highest activity (31 mmol C_4_ per Ti atom per minute) and the greatest selectivity toward C_4_ mono-olefins. This superior performance is attributed to the high concentration of unpaired electrons, which are proposed to enhance the nucleophilic character of the catalyst. Interestingly, commercial TiC with an identical composition showed significantly lower activity (8.6 mmol C_4_ per Ti atom per minute) and selectivity compared to the MXene-based catalyst (Figure 33). In contrast, a physical mixture of anatase and rutile TiO_2_ demonstrated better performance than pure phase alone; however, its C_4_ formation rate remained well below that of Ti_2_CT_x_ MXene. These data clearly show the better performance of Ti_2_CT_*x*_ MXene with respect to the corresponding MAX or derived products, the activity being correlated with surface defects and partial oxidation. In ODH, adsorbed butane gives a butyl radical and donates a H atom to the surface, this being the rate-determining step. The resulting butyl intermediate will donate another H atom to another surface site, forming butene. The key point in achieving better selectivity is a fast alkene desorption, minimizing overoxidation. Surface hydrogens react independently with oxygen, giving H_2_O as a byproduct. Apparently, surface defects in Ti MXenes are very appropriate sites to accept H atoms from butane, therefore giving a hint for future optimization of the catalytic activity

**Figure 33:**
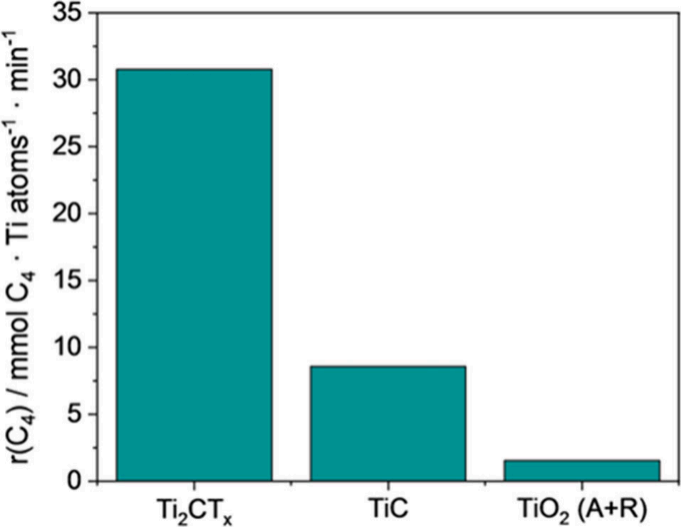
C_4_ olefin formation rate of Ti_2_CT_*x*_ and two other related materials as catalysts after 0.5 h under reaction at 500 °C and O_2_/butane = 1:1. A+R denotes anatase+rutile titania. Reproduced with permission from ref. 108. Copyright 2022 Wiley.

In another report, based on theoretical calculations, hydrogen affinity was proposed as a quantitative activity descriptor to characterize the catalytic activity of various termination configurations of Ti_2_CT_*x*_ (T = −O, −OH) MXenes in propane dehydrogenation reactions.^109^ First-principles calculations demonstrated that hydrogen affinity can be regarded as an intrinsic property of −O and −OH terminations in Ti_2_C MXenes, with the mean hydrogen affinity for the terminated Ti_2_C MXenes showing a linear correlation with the statistical average of their −OH fraction. Furthermore, the C–H activation energies exhibited a clear scaling relationship with hydrogen affinity. Thus, it can be concluded that hydrogen affinity serves as a reliable indicator of MXenes performance in this reaction. These theoretical insights are valuable for guiding the rational design and synthesis of more efficient MXene catalysts and provide insights into the nature of surface terminations acting as catalytic sites.

Building upon the catalytic role of surface defects and vacancy sites, it is important to highlight that such structural irregularities can also enable MXenes to function in oxidation reactions. Although earlier sections have raised concerns about the oxidative stability of MXenes, particularly their susceptibility to degradation under oxidizing conditions, a growing number of studies have nonetheless explored their application in oxidation catalysis. These investigations suggest that defect-rich surfaces, especially those featuring low-valence metal centers, can effectively activate molecular oxygen or related oxidants. A representative example is the use of monolayer V_2_CT_*x*_ nanosheets, synthesized via hydrothermal acid etching and organic macromolecule intercalation, for the oxidation of dibenzothiophene using molecular O_2_ from air at 70 °C.^181^ This reaction is of practical interest for the deep desulfurization of diesel, as the resulting sulfone is water-soluble and can thus be easily separated from the hydrophobic hydrocarbon fuel. Despite their known vulnerability to oxidation, these V_2_CT_*x*_-based materials achieved promising catalytic performance, highlighting the potential of carefully engineered MXene surfaces in aerobic oxidation processes. The performance of FL-V_2_CT_*x*_ (FL: few-layers) reached 100% conversion of dibenzothiophene, whereas ML-V_2_CT_*x*_ (ML: multilayer) achieved only 75% under identical conditions. The superior performance of the few-layer material was ascribed to the existence of low-valence V species accompanied by vacancies on the surface of MXene, which were proposed to exhibit excellent oxygen activation capacities based on first-principles calculations.^181^ The catalyst stability was assessed over six consecutive uses without apparent structural alteration. However, a thorough comparison of all structural properties before and after catalysis would be necessary to conclusively demonstrate the stability of f-V_2_CT_*x*_ under aerobic oxidation reactions. Similarly, V_2_C was found to promote the aerobic oxidation of indane to indanol/indanone mixture, although characterization of the resulting MXene showed its complete conversion to VOx under solventless conditions consisting at 120 °C.^94^

Formic acid is one of the promising hydrogen carriers, which is renewable, safe, and environmentally tolerable. Although noble-metal-based catalysts have been widely reported for the dehydrogenation of formic acid with high activity, the development of noble-metal-free heterogeneous catalysts with high efficiency is always a challenge. In this regard, oxygen coverage on the surface of Ti_3_C_2_T_*x*_ MXenes was modulated, and the resulting solid performance was studied in the formic acid dehydrogenation.^182^ Interestingly, Ti_3_C_2_T_*x*_-250 (the material where Ti_3_C_2_T_*x*_ MXene was treated with air at 250 °C) significantly enhanced the density of surface oxygen atoms without affecting its crystallinity and showed the production of 365 mmol g^–1^ h^–1^ with 100% selectivity at 80 °C. This performance was 2.2 and 2 folds higher compared to the commercial Pd/C and Pt/C solids, respectively (Figure 34). Furthermore, mechanistic studies by *in situ* DRIFT spectroscopy suggest that HCOO* is the intermediate in formic acid dehydrogenation. Interestingly, increasing the oxygen coverage on the surface of Ti_3_C_2_T_*x*_ MXenes not only favors the conversion of HCOO* to CO_2_* by reducing the energy barrier but also weakens the adsorption energy of CO_2_ and H_2_. These results illustrate again the important role of the surface termination in MXene catalysis.

**Figure 34:**
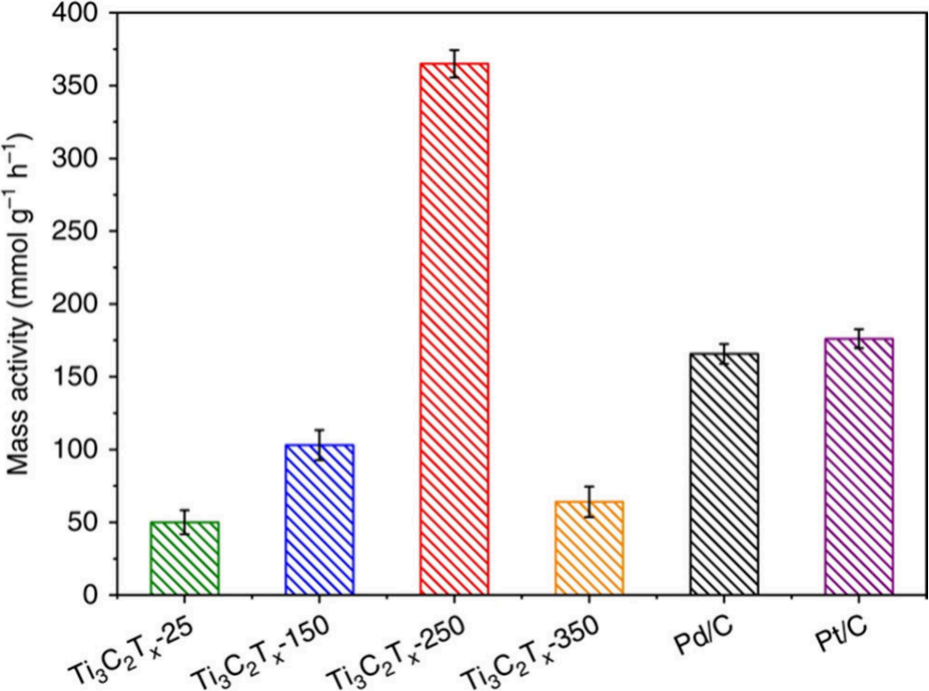
Comparison of the activities of different MXenes with the commercial catalysts. Reproduced with permission from ref. 182. Copyright 2020 Nature Portfolio.

Deeva and co-workers demonstrated that Mo_2_CT_*x*_ is a highly stable and active catalyst for the water–gas shift reaction, achieving >99% selectively toward CO_2_ and H_2_ at 500 °C.^110^ The carbon monoxide conversion over Mo_2_CT_*x*_ begins to decline at temperatures where the interlayer distance between carbide sheets decreases (600–730 °C), as evidenced by XRD-monitored TPR, indicating mass transfer limitations under these conditions. These findings underscore the importance of both thermal stability and structural integrity in designing effective MXene-based catalysts for high-temperature redox reactions.

Overall, surface defects and vacancies endow MXenes with notable catalytic activity, particularly in oxidation reactions. Low-valence metal centers and unpaired electrons facilitate reactant activation, enabling efficient processes such as oxidative dehydrogenation and oxidative desulfurization. Compared to bulk analogs, defect-rich MXenes like Ti_2_CT_*x*_ and V_2_CT_*x*_ show enhanced performance. Despite concerns over oxidative stability, these results highlight defect engineering as a viable strategy to boost MXene reactivity while preserving structural integrity under suitable conditions.

### 5.3 MXenes as Supports for Single Atoms and NPs

The development of SACs in heterogeneous catalysis has opened new research avenues, enabling the highly efficient use of precious metals and supporting promising applications across diverse reactions.^183^ One of the main challenges in SACs is the synthesis of thermally and chemically stable solids that can withstand reaction conditions without undergoing agglomeration or leaching of the SAs. To mitigate these deactivation pathways, the nature and structure of the support are crucial, since it must provide anchoring sites with sufficiently strong interactions to stabilize isolated metal centers. As commented in Section 2, the harsh etching conditions required for MXenes synthesis from MAX precursors generate metal-atom vacancies that are well suited to accommodate SAs. Accordingly, MXenes can serve as a versatile platform for both (*i*) vacancy-anchored SACs (and, in some cases, dual-atom sites) and (*ii*) strongly interfaced metal NPs or NCs whose electronic structures are tuned by MSIs. Thus, as a collateral process accompanying “*A*”-element removal during MAX-to-MXene etching, vacancies are generated in the “*M*” layers. These *M* vacancies can be replenished during etching or subsequent post-treatments by incoming metal species, yielding vacancy-trapped SA (or dual-atom) centers. Alternatively, SACs can be located on top of the surface terminal groups (T_*x*_) while maintaining strong interaction with these terminations. This combination of intrinsic vacancy chemistry,^100^ rich surface terminations (−O, −F, −OH, −Cl), and high conductivity makes MXenes as attractive supports for SAs and supported metal entities, as confirmed by atomic-resolution microscopy and X-ray absorption spectroscopy, which can probe coordination environments even at very low metal loadings. Rather than relying on intrinsic activity of the MXene itself, the predominant role of MXenes in thermal catalysis to date has been to employ them as supports that stabilize and electronically modulate atomically dispersed metals and metal NPs. To improve clarity, this section is organized into discrete subsections according to reaction class (response type), while explicitly highlighting whether the active phase is a SA/dual-atom site or an NP/NC entity.

#### 5.3.1 Design Principles and Anchoring Motifs (SAs versus NPs)

First-principles calculations based on DFT provide fundamental insights into the ability of MXene carbides to stabilize isolated metals.^184^ A detailed and systematic investigation using ten 3d transition metals and nine bare MXene surfaces with M_2_C stoichiometry (M = Ti, V, Cr, Zr, Nb, Mo, Hf, Ta, and W) indicated that 3d metal atoms interact exothermically with MXene supports and that the properties of M@MXene can be tuned by appropriate selection of both the metal and the MXene host.^184^ These findings suggest that atomically dispersed metals can be stabilized on MXene surfaces and that their charge densities can be tuned from partially oxidized to partially reduced states. Notably, Zn atoms anchored on MXenes appear especially promising because clustering is thermodynamically unfavorable and surface diffusion is impeded by moderate energy barriers. More broadly, these calculations establish two recurring stabilization motifs: (*i*) vacancy trapping within the M layer (strong metal–carbide coordination) and (*ii*) coordination to surface terminations (metal–T_*x*_ interactions), which can operate either alone or cooperatively.

A distinguishing feature of MXenes is that the bonds formed between anchored metals and the X/M layers can be stronger and more diverse than those typically achieved on common SAC supports (often limited to three or four N or O donor atoms). In contrast, MXene hosts can offer higher coordination numbers and stronger metal–carbide interactions, which can enhance thermal stability and suppress sintering. For metal NPs and NCs, strong MSIs and, in some systems, reactive MSIs that generate intermetallic interfaces, are frequently invoked to explain improved dispersion, altered adsorption energetics, and enhanced resistance to coking or leaching. These concepts are repeatedly encountered across the reaction classes summarized below. Taken together, these features define a general design framework in which MXenes act not only as structural anchors but also as electronic regulators for both SAs and metal NPs, providing transferable design rules that recur across the reaction classes summarized below.

#### 5.3.2 Hydrogenation and Reductions (SAs, NCs, and NPs)

In Ti_3_C_2_T_*x*_ MXenes, Pt or Pd (0.1–1 wt %) were deposited through wet impregnation via sonication of Pt or Pd chlorides into MXene dispersions.^128^ The resulting catalysts were evaluated in gas-phase butadiene hydrogenation and CO_2_ hydrogenation under flow conditions.^128^ Ti_3_C_2_T_*x*_ MXenes were prepared either by HF etching (CMX, C meaning commercial, MX referring to MXene) or by LiF-HCl etching (PMX, P meaning prepared at Poitiers). Two LiF-HCl samples were obtained, with PMX2 being more defective and oxidized than PMX1. In general, Pd/PMX1 was more effective for butadiene semihydrogenation, whereas Pt/PMX2 performed better in CO_2_ hydrogenation.^128^ Notably, 0.1Pt/PMX SACs exhibited an unusual hydrogenation mechanism compared with alumina-supported catalysts and the 1Pt/PMX analog, yielding higher selectivity to 2-butenes without butane formation. Remarkably, 0.1Pt/PMX achieved CO selectivities of 98–99% in the reverse water–gas shift reaction, with a Pt molar activity exceeding that of tested oxide-supported catalysts. These observations suggest that the oxidation state of surface Ti plays a significant role in selectivity control.

Hydrogenation of multiple carbon bonds is a vital transformation in organic synthesis. MXenes have been employed as solid supports for metal NPs, where the NPs are anchored via electrostatic interactions and subsequently used in selective hydrogenation.^158^ A galvanic replacement strategy was used to construct tripodal Pd metallenes on Nb_2_C MXenes at room temperature (Pd/Nb_2_C).^126^ DFT calculations and molecular dynamic simulations supported strong interactions between Pd and Nb atoms. Due to lattice mismatch, Pd metallenes adopt a nonplanar chairlike configuration, resulting in a unique tripodal geometry and remarkable catalytic performance. A 0.5 wt % Pd/Nb_2_C catalyst achieved 96% selectivity in the semihydrogenation of phenylacetylene with a TOF value of 10372 h^–1^. This selectivity is significantly higher than that of conventional Pd NPs, which often promote overhydrogenation. In addition, H_2_-Pd/Nb_2_C demonstrated a 15-fold higher TOF than a Lindlar catalyst (Table 2). DFT calculations suggested that weaker adsorption and enhanced diffusion away from Pd centers suppress overhydrogenation. The catalyst maintained 80% conversion and 96% selectivity over six cycles, and the Pd metallene size distribution remained unchanged. However, given the known oxidation sensitivity of MXenes, stability should be substantiated by complementary structural and chemical analyses (Scheme 4).

Three Pd/Nb_2_C catalysts prepared using different reduction methods (SB, pyrolysis, and H_2_ treatment) were evaluated in phenylacetylene semihydrogenation.^154^ Among them, H_2_-Pd/Nb_2_C showed superior performance, achieving a TOF of 7263 h^–1^ and 93.5% selectivity to styrene, and retained activity over five cycles (Figure 35). The superior performance of H_2_-Pd/Nb_2_C was ascribed to a strong MSIs and facile electron transfer from Nb to Pd, generating electron-rich Pd species and a unique Pd-Nb_2_C interface.

**Figure 35:**
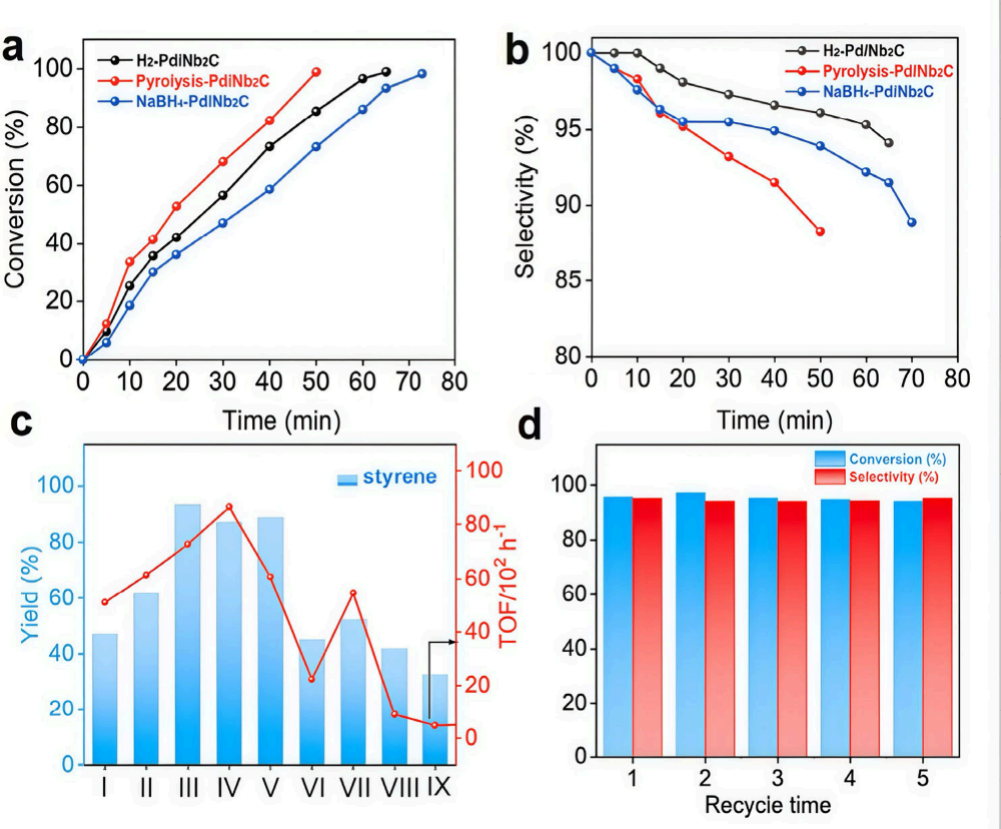
(a) Conversion of phenylacetylene as a function of time using H_2_-Pd/Nb_2_C, Pyrolysis-Pd/Nb_2_C and SB-Pd/Nb_2_C as catalysts. (b) Selectivity toward styrene over time in phenylacetylene semihydrogenation catalyzed by H_2_-Pd/Nb_2_C, Pyrolysis-Pd/Nb_2_C, and SB-Pd/Nb_2_C. (c) TOF values and yields of products in phenylacetylene semihydrogenation for various supported Pd catalysts. I to IX correspond to Pd/C, Pd/Nb_2_O_5_, H_2_-Pd/Nb_2_C, Pyrolysis-Pd/Nb_2_C, SB-Pd/Nb_2_C, Pd/V_2_O_5_, Pd/TiO_2_, Pd/Al_2_O_3_, and Lindlar, respectively. (d) Catalytic stability for H_2_-Pd/Nb_2_C. Reproduced with permission from ref. 154. Copyright 2023 Elsevier.

Highly dispersed Ru NCs were supported on Ti_3_C_2_T_*x*_ nanosheets (RuNCs/Ti_3_C_2_T_*x*_) exploiting the 2D structure and abundant surface terminations (Scheme 9).^156^ RuNCs/Ti_3_C_2_T_*x*_-3 (where 3 indicates 3 wt % Ru NCs) achieved complete conversion and selectivity in quinoline hydrogenation at 55 °C and 5 bar H_2_ in a water–ethanol mixture.^156^ Theoretical calculations indicated that Ti_3_C_2_T_*x*_ tailors the electronic structure of Ru through MSIs, promoting desorption of H from Ru and enhancing activity. Nevertheless, key metrics such as metal leaching and long-term stability were not reported and should be addressed when benchmarking MXene-supported Ru systems.^156^

**Scheme 9:**
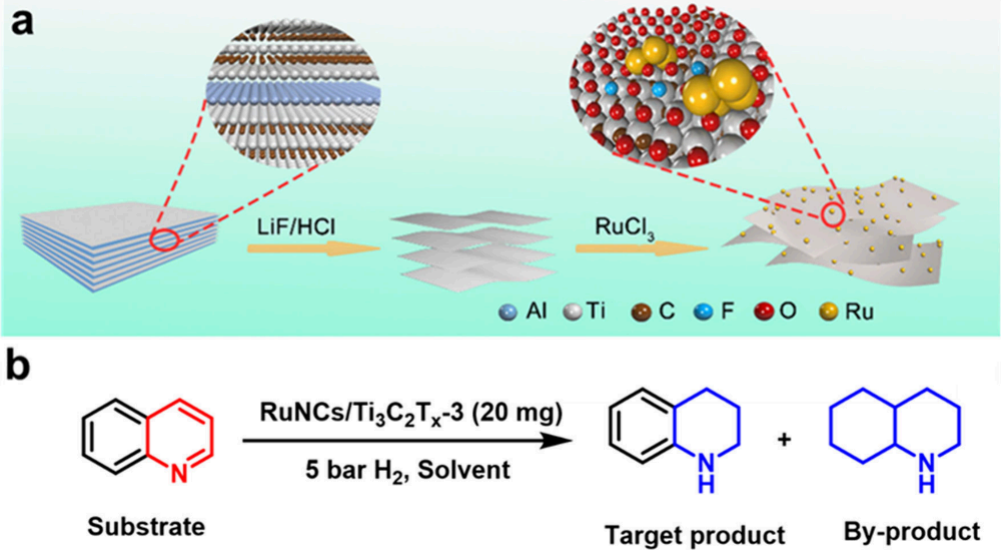
(a) Illustration of the Preparation and Structure of RuNCs/Ti_3_C_2_T_*x*_ Catalysts. Reproduced with Permission from ref. 156. Copyright 2022 American Chemical Society. (b) Hydrogenation of Quinoline Using RuNCs/Ti_3_C_2_T_*x*_-3 Solid

Pt NPs were deposited onto Ti_3_C_2_T_*x*_-derived MXene and evaluated for selective reduction of nitroaromatic compounds to anilines.^158^ Using AB or SB as reducing agents (Scheme 10), Pt/Ti_3_C_2_T_*x*_D-AB (2.2 nm Pt) achieved 100% conversion and 99.5% selectivity for 4-chloronitrobenzene within 1 h, whereas Pt/Ti_3_C_2_T_*x*_D-SB was less active.^158^ Pt/Ti_3_C_2_T_*x*_D-AB achieved a TOF of 3.9 × 10^6^ h^–1^, exceeding benchmark Ni SAs on N-doped carbon (8.4 h^–1^; Table 2), and maintained performance over six cycles. Ag NPs deposited on reduced Ti_3_C_2_T_*x*_ using l-arginine (Ag/r-Ti_3_C_2_T_*x*_) showed a TOF of 1109 h^–1^ for 4-nitrophenol reduction.^160^ The protective role of l-arginine against oxidation was proposed, highlighting MXene stability as a recurring concern (Scheme 4). Ag/r-Ti_3_C_2_T_*x*_ retained 90% activity after five cycles.^160^

**Scheme 10:**
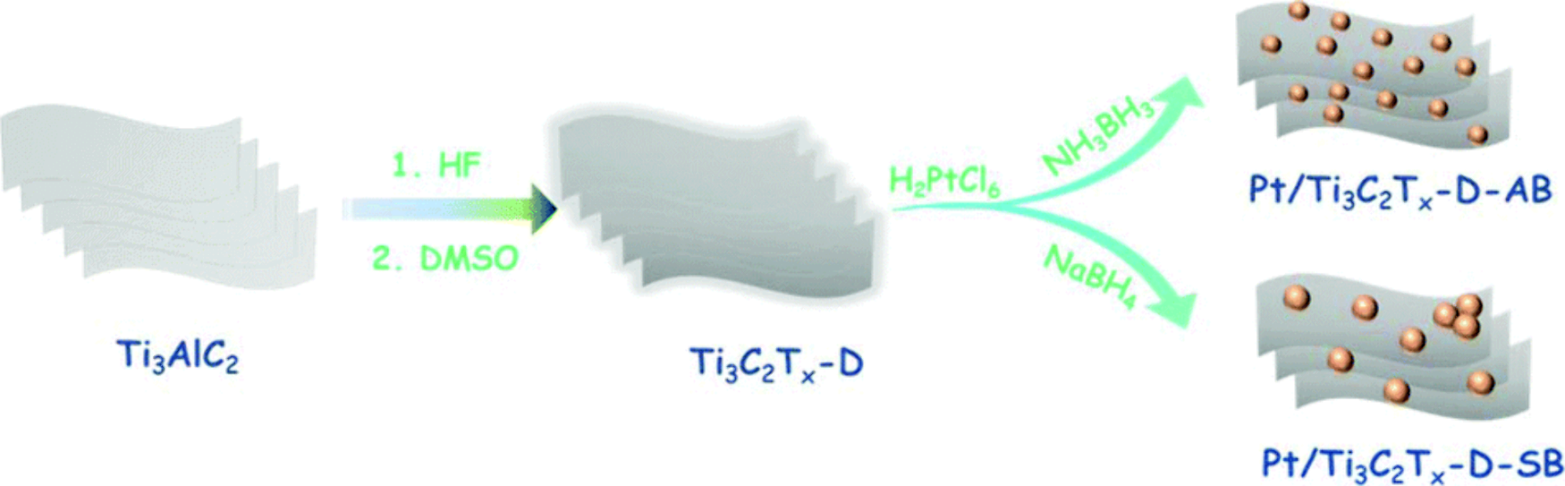
Preparation of Ti_3_C_2_T_*x*_-D and the Deposition of Pt NPs to Obtain Pt/Ti_3_C_2_T_*x*_-D-AB and Pt/Ti_3_C_2_T_*x*_-D-SB. Reproduced with Permission from ref. 158. Copyright 2020 Royal Society of Chemistry

Taken together, these studies demonstrate that MXenes are highly effective supports for hydrogenation and reduction reactions, enabling both vacancy-anchored SACs and strongly interfaced NP/NC motifs across a wide range of substrates. The presence of metal vacancies, tunable surface terminations, and strong MSIs enables precise control over metal dispersion and electronic structure, thereby enhancing activity and selectivity while suppressing undesired overhydrogenation pathways. In several systems, electron transfer from the MXene support to the anchored metal species plays a decisive role in modulating adsorption strength and reaction kinetics. At the same time, the recurring sensitivity of MXenes to surface oxidation and potential metal leaching highlights the importance of systematic stability evaluation and postreaction characterization, particularly for reactions conducted under reductive or liquid-phase conditions (Scheme 4).

#### 5.3.3 Dehydrogenation (Dual-Atom Sites, NPs, and Supported Pt Layers)

Ethane dehydrogenation to ethylene has attracted increasing interest due to its lower carbon footprint compared with conventional steam cracking, yet it remains kinetically limited by the high activation energy required for C–H bond cleavage.^185^ Using first-principles calculations, Wan and co-workers proposed a double-atom catalyst concept based on MXenes.^161^ In this theoretical study, Rh_2_@V_2_CO_2_ exhibited strong d−σ* coupling between ethane, leading to low calculated energy barriers of 0.64 and 0.63 eV for the successive C–H activation steps. Although experimental validation is still lacking, this work illustrates the potential of MXenes to stabilize cooperative multiatom active motifs beyond conventional single-atom catalysts.

In addition to atomically dispersed sites, MXenes have been extensively explored as supports for metal NPs in dehydrogenation-related reactions. Bimetallic RhNi NPs uniformly dispersed on Ti_3_C_2_ MXene (RhNi/MXene) showed complete selectivity toward H_2_ in hydrous hydrazine decomposition, achieving a TOF of 857 h^–1^, which is substantially higher than that of RhNi catalysts supported on ZIF-8 and conventional carbon or oxide materials.^163^ The catalyst maintained stable performance over multiple cycles (Figure 36), highlighting the role of strong MSIs and efficient charge transfer enabled by the conductive MXene support.

**Figure 36:**
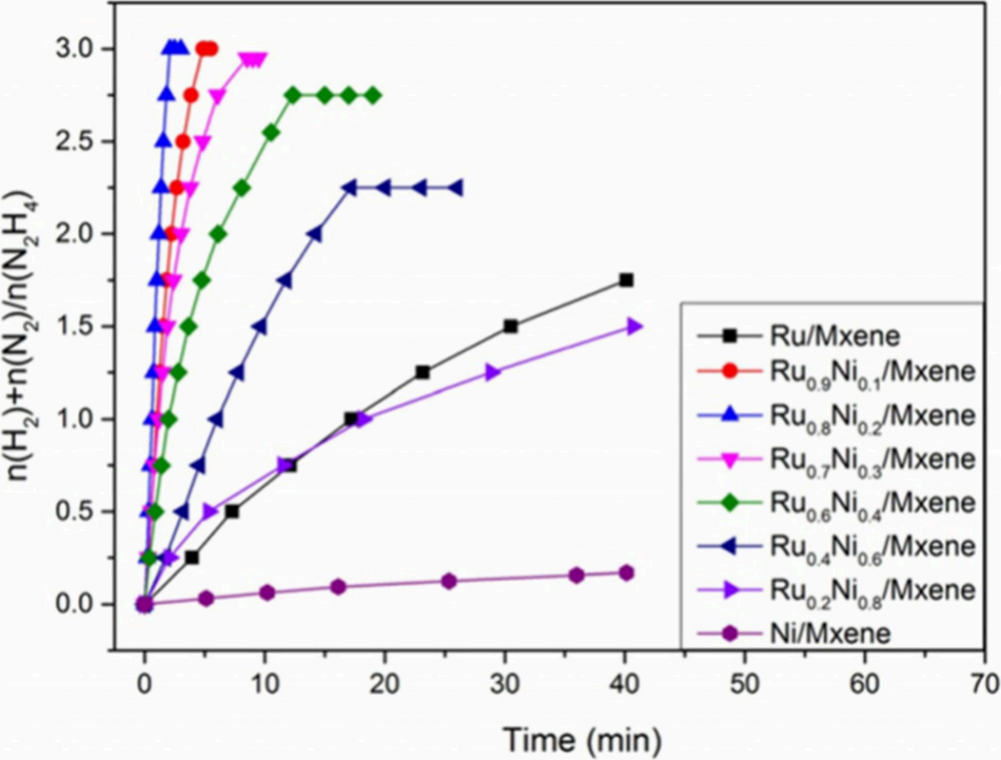
Time-course plots for H_2_ generation from the decomposition of hydrazine hydrate with different Rh/Ni molar ratios supported over MXene at 50 °C. Reproduced with permission from ref. 163. Copyright 2018 Wiley.

Hydrogen release from chemical hydrogen carriers provides another representative class of dehydrogenation reactions.^186^ Pt NPs supported on Ti_3_C_2_T_*x*_-derived MXenes exhibited strongly support-dependent activity in AB hydrolysis, which could be markedly enhanced by controlled oxidation of the MXene surface.^166^ Ozone- and H_2_O_2_-treated MXenes significantly modified the electronic structure of supported Pt, resulting in TOF values exceeding 260 min^–1^ at 30 °C, far higher than those of untreated Pt/MXene and benchmark Pt/TiO_2_ catalysts (Table 2). This study demonstrates that deliberate tuning of MXene surface chemistry offers an effective handle to modulate MSIs and dehydrogenation kinetics, albeit with careful consideration of structural stability.

Functionalized MXenes can also stabilize alloy NPs for dehydrogenation reactions. In this context, bimetallic PdCr NPs immobilized on amine-functionalized Ti_3_C_2_ MXenes (PdCr/NH_2_-MXene) exhibited high activity for formic acid dehydrogenation, reaching a TOF of 1906 h^–1^ at 50 °C (Figure 37).^169^ The catalyst showed no significant activity decay over five cycles, and TEM analysis confirmed the preservation of NP size after reuses. Although the surface chemistry is more complex than simple −NH_2_ termination, this system illustrates how surface functionalization of MXenes can promote uniform dispersion and electronic modulation of alloy NPs.

**Figure 37:**
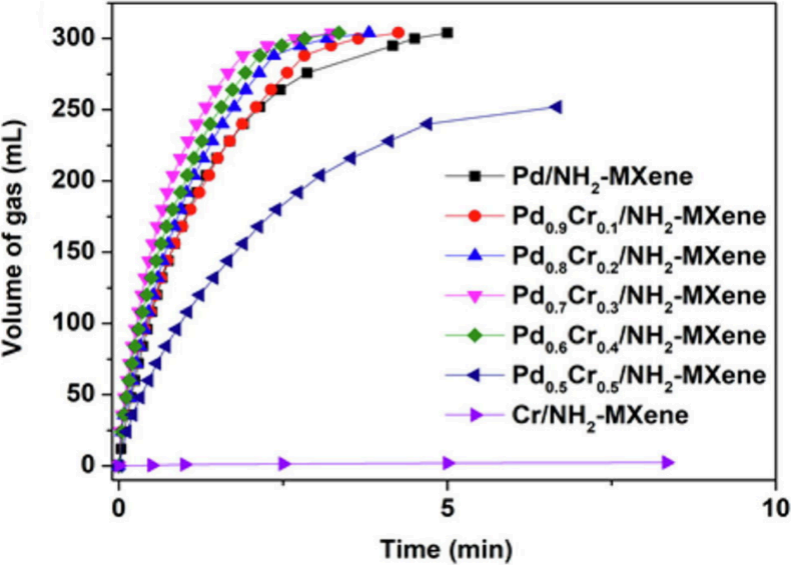
Dehydrogenation of formic acid with time using Pd_1–*x*_Cr_*x*_/NH_2_-MXene catalysts. Reproduced with permission from ref. 169. Copyright 2022 Elsevier.

A related boundary example involves a partially leached Ti_3_(Al_0.8_Sn_0.2_)­C_2_ MAX phase (TASC-NaOH), which likely contains MXene-like surface but cannot be classified as a true MXene.^167^ Ru NPs supported on this disordered material exhibited high activity for AB hydrolysis, with a TOF of 582 min^–1^ at 30 °C (Figure 38). However, gradual activity loss due to Ru detachment underscores the importance of well-defined MXene structures for achieving durable metal anchoring and reliable catalytic performance.

**Figure 38:**
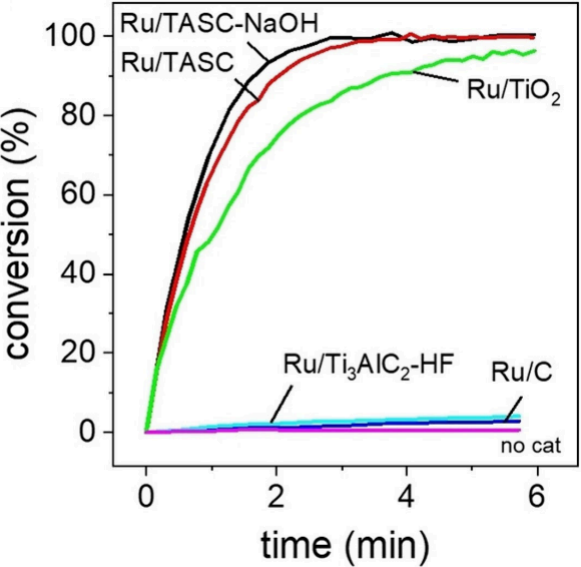
Reduction of 4-nitroaniline by AB catalyzed by various Ru-impregnated catalysts. Reproduced with permission from ref. 167. Copyright 2021 Wiley.

Beyond metal-centered systems, interfacial coupling in MXene-based heterostructures can also promote dehydrogenation.^164^ For example, a Ti_3_C_2_@PrF_3_ nanosheet heterojunction enabled efficient low-temperature dehydrogenation of AlH_3_, which was attributed to synergistic electronic redistribution across the interface and the intimate contact between the two phases (Scheme 11).^164^ Although this system does not fall within the archetype of MXene-supported metal SAs or NPs, it further highlights the importance of interfacial electronic effects in MXene-enabled dehydrogenation chemistry.

**Scheme 11:**
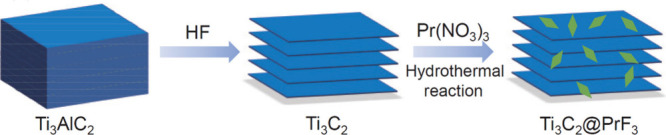
Preparation Procedure and Structure of Ti_3_C_2_@PrF_3_. Note That the PrF_3_ Is Formed from the F Atoms of the Ti_3_C_2_ Surface. Reproduced with permission from ref. 164. Copyright 2023 Springer

In petrochemical dehydrogenation, catalyst deactivation by coking remains a major challenge. In this context, MXenes display notable advantages. Atomically thin Pt nanolayers deposited on Mo_2_TiC_2_ MXene catalyzed the nonoxidative dehydrogenation of ethane and propane with high selectivity (>95%) and stable activity over 24 h on stream without detectable coke formation (Figure 39).^125^ The strong resistance to coking is attributed to the unique electronic properties of MXenes combined with robust MSIs.^187^

**Figure 39:**
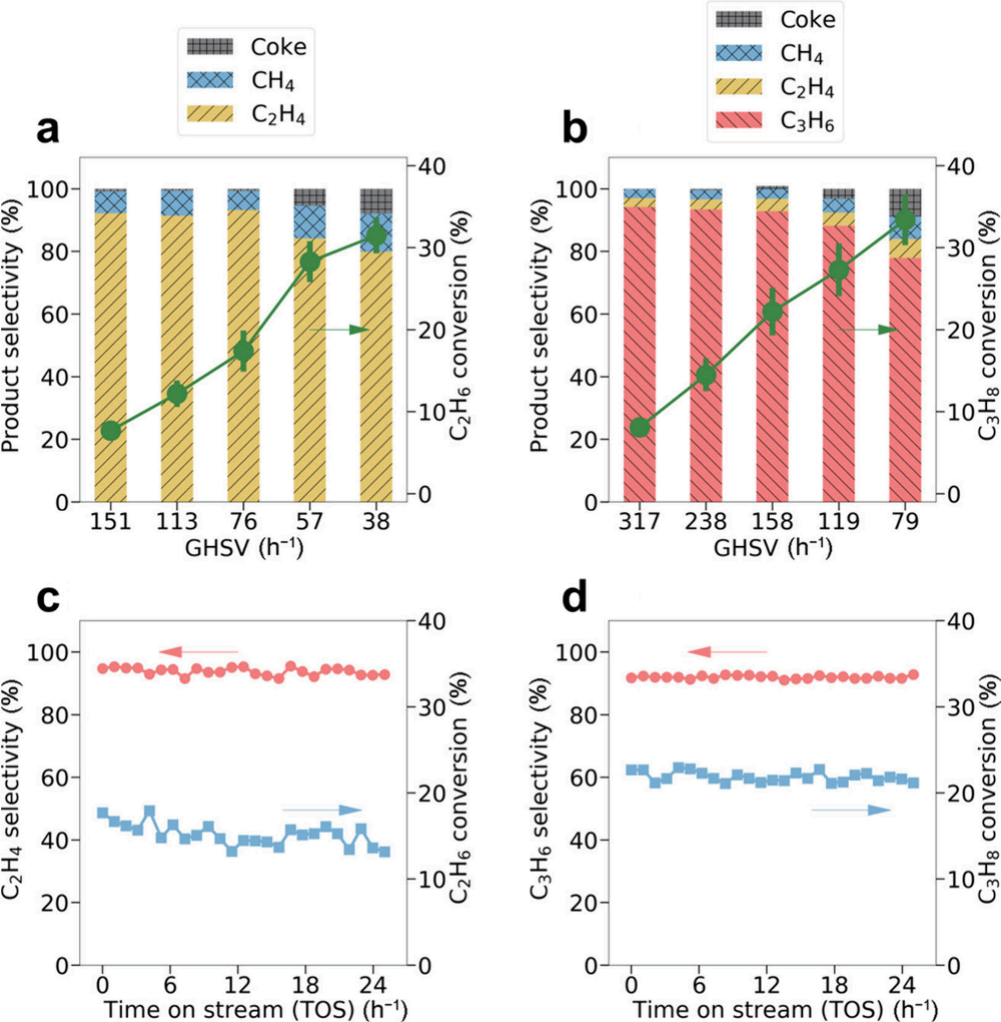
Performance of the 0.5% Pt/Mo_2_TiC_2_ catalyst for nonoxidative ethane and propane dehydrogenation. (a) Effect of gas hourly space velocity (GHSV) on C_2_H_6_ dehydrogenation. (b) Effect of GHSV on C_3_H_8_ dehydrogenation. (c) Catalyst stability of C_2_H_6_ dehydrogenation. (d) Catalyst stability of C_3_H_8_ dehydrogenation. Operating conditions at 550 °C, 10% C_2_H_6_ or 10% C_3_H_8_ with balanced 89% N_2_ and 1% Ar as internal standard, 200 cc/min total flow rate, 200 or 100 mg catalyst for dehydrogenation of ethane or propane, respectively. Reproduced with permission from ref. 125. Copyright 2024 Springer.

Overall, these examples demonstrate that MXenes can stabilize a diverse range of dehydrogenation-active motifs, including dual-atom sites, alloy NPs, and interfacial heterostructures. Their high conductivity, tunable surface chemistry, and strong MSIs collectively enable enhanced activity, selectivity, and resistance to deactivation, establishing MXenes as versatile supports for dehydrogenation catalysis.^47,125,187^

#### 5.3.4 CO_2_ Conversion and Reforming (SAs, NPs, and Intermetallic Interfaces)

Designing efficient catalysts for CO_2_ conversion and reforming is of central importance for mitigating carbon emissions and enabling sustainable chemical transformations. In this context, MXenes have emerged as versatile supports for both SAs and metal NPs, owing to their defect-rich surfaces, high electrical conductivity, and strong MSIs that collectively facilitate CO_2_ activation and subsequent transformation.^188^

From a theoretical perspective, DFT calculations have predicted that SAs stabilized on MXene surface can serve as highly selective active sites for CO_2_ hydrogenation. For example, Bi SAs anchored on V_2_C MXenes (Bi@V_2_C) were proposed to selectively hydrogenate CO_2_ to formic acid, while suppressing the competing reverse water–gas shift reaction.^171^ Similarly, Ni SAs immobilized at oxygen-vacancy sites of Ti_3_C_2_O_2_ (Ni@Ti_3_C_2_O_2_) were shown to catalyze CO_2_ hydrogenation to formic acid via a low-barrier pathway involving a HCOO* intermediate.^173^ These studies collectively highlight the unique capability of MXene vacancy sites to stabilize isolated metal centers with well-defined coordination environments and favorable CO_2_ adsorption energetics.

Beyond isolated atoms, MXene-supported metal NPs have also demonstrated promising performance in CO_2_ conversion reactions. A systematic theoretical investigation of ordered Mo_2_TiC_2_T_*x*_ revealed that defect formation and catalytic properties strongly depend on surface terminations.^174^ CO_2_ adsorption on defect-free MXene surfaces was found to be endothermic, whereas adsorption at vacancy sites was spontaneous and exothermic. This finding underscores the importance of deliberate defect engineering in transforming MXenes from passive supports into catalytically active interfaces for CO_2_ activation.^174^ Experimentally, the active role of MXenes in CO_2_ hydrogenation has been clearly demonstrated. A silica-supported Cu/Mo_2_CT_*x*_ catalyst prepared via surface organometallic chemistry exhibited a higher intrinsic CH_3_OH formation rate than a reference^129^ Cu/SiO_2_ catalyst at identical Cu loading (Figure 40).^129^ Although both catalysts displayed similar Cu NP sizes, the Mo_2_CT_*x*_ surface enabled superior dispersion, including the stabilization of SAs and small clusters. During H_2_ treatment, Cu species were observed to migrate from silica onto the Mo_2_CT_*x*_ surface, forming stabilized Cu^+^ sites at the Cu–Mo_2_CT_*x*_ interface. Operando XANES and XPS analyses confirmed the structural stability of the MXene support during prolonged operation, correlating with sustained CH_3_OH productivity. This work illustrates that MXenes can actively participate in stabilizing catalytically relevant oxidation states and reaction intermediates rather than merely serving as inert carriers.

**Figure 40:**
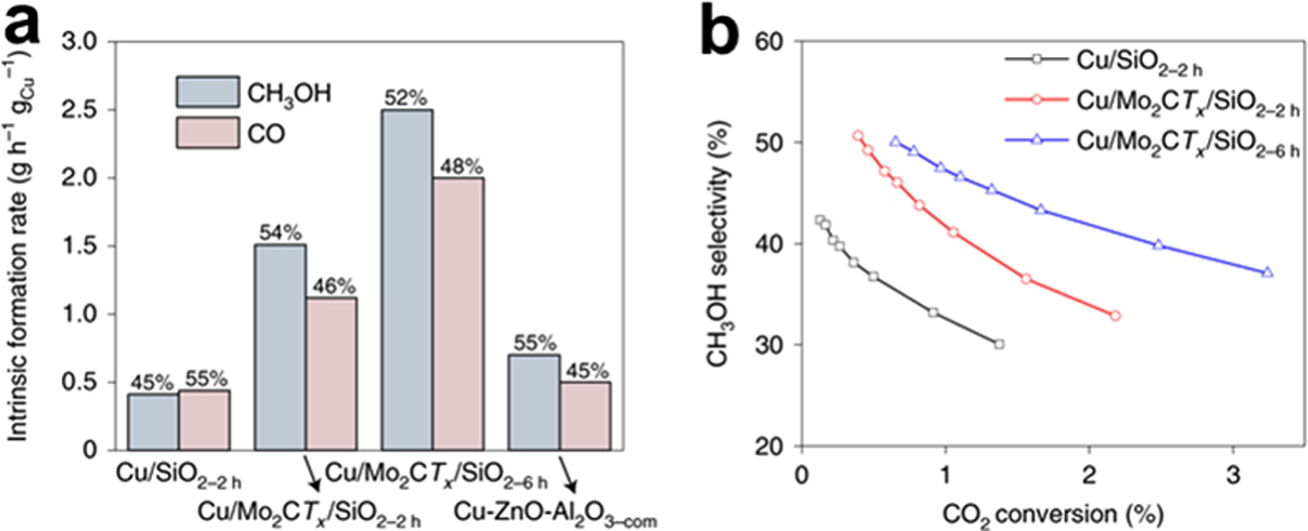
(a) Comparison of intrinsic formation rates of CH_3_OH and CO for Cu/Mo_2_CT_*x*_/SiO_2_ and Cu/SiO_2_ catalysts (230 °C, 25 bar, H_2_/CO_2_/N_2_ = 3/1/1) obtained by extrapolation to zero conversion together with the selectivity values for CH_3_OH and CO specified above the respective bars. The Cu–ZnO–Al_2_O_3_–com denotes the commercial Cu–ZnO–Al_2_O_3_ catalyst (pretreated in H_2_ at 250 °C for 3.5 h before the catalytic test). In addition, 2 and 6 h indicate the prereduction at 500 °C either for 2 or 6 h. (b) Dependence of CH_3_OH selectivity on CO_2_ conversion by varying the contact time. Reproduced with permission from ref. 129. Copyright 2021 Nature Portfolio.

Surface modifications of MXenes further provide an effective handle for tuning CO_2_ conversion selectivity. Nitrogen-doped Ti_3_C_2_T_*x*_ obtained via NH_3_ treatment altered the catalytic behavior of supported Co NPs, shifting selectivity from CO to CH_4_ during CO_2_ hydrogenation (Figure 41).^124^ This effect was attributed to the formation of TiO_2_ species and strengthened Co–TiO_2_ interactions, which modified the reducibility of Co at the interface. Both pristine and N-doped catalysts exhibited stable performance over extended operation. This example highlights the ability of MXene surface chemistry to regulate MSIs and steer reaction pathways.

**Figure 41:**
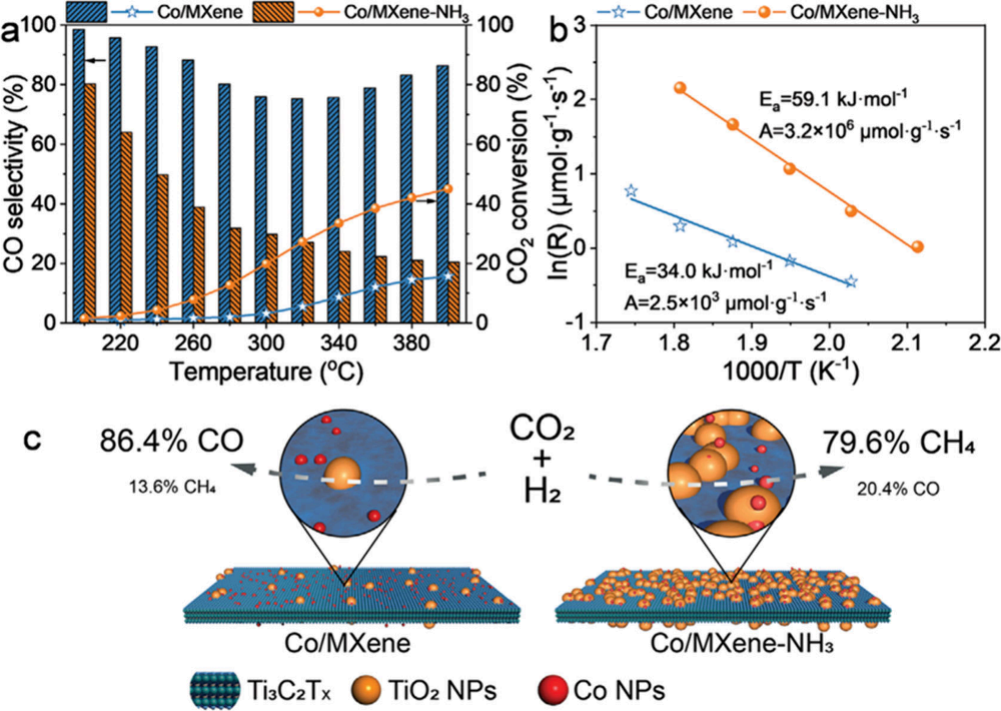
(a) Catalytic performance of CO_2_ hydrogenation for Co/MXene and Co/MXene-NH_3_ as a function of reaction temperature and (b) Arrhenius plot of catalysts for CO_2_ hydrogenation. Reaction conditions: 100 mg of catalysts, *T* = 200–400 °C, flow rate 40 mL min^–1^, and CO_2_/H_2_/N_2_ = 24%/72%/4%. (c) Schematic illustration of reaction pathway over Co NPs supported on the surface modified MXene. Reproduced with permission from ref. 124. Copyright 2021 Wiley.

MXenes have also proven as advantageous supports for dry reforming of CH_4_.^189^ Ni NPs supported on multilayer V_2_C-derived MXenes achieved high CH_4_ and CO_2_ conversions with excellent stability over extended operation, which was attributed to strong MSIs that suppressed coke formation.^190^ *In situ* DRIFTS analysis revealed that CH_4_ activation occurred on Ni sites, while CO_2_ was adsorbed on the MXene surface as carbonate species that subsequently reacted with CH_4_-derived intermediates to form CO. These results emphasize the cooperative roles of metal NPs and MXene surfaces in complex reforming reactions.

As discussed in Section 3, reactive MSIs are not uncommon with MXene as supports and often lead to the formation of intermetallic interfaces. Such reactive MSIs are crucial for tuning the electronic density of active metals, controlling particle geometry, and enhancing both activity and stability. In this respect, the interaction between Pt and Nb_2_CT_*x*_ MXene has been studied to determine the influence of the MSI on the catalytic activity of the material for the water–gas shift reaction.^47^ After reductive removal of surface functional groups at 350 °C, a Pt–Nb surface alloy was formed. This alloy exhibited weaker CO adsorption compared to monometallic Pt and improved H_2_O activation relative to Pt supported on nonreducible oxides or bulk Nb_2_C.^47^ These findings demonstrate the importance of reactive MSIs in forming catalytically favorable interfaces and highlight the potential of extending such strategies to other MXenes.

CO_2_ activation has been widely studied using SACs.^191^ For example, Pt SACs (0.2 wt %) were stabilized on Ti_3–*x*_C_2_T_*y*_ MXene nanosheets via simultaneous self-reduction of PtCl_6_^2–^ and deposition of Pt at Ti vacancy sites at room temperature.^113^ The abundant Ti vacancies in Ti_3–*x*_C_2_T_*y*_ are particularly favorable for anchoring Pt SAs, which form strong Pt–C bonds with the MXene and become stabilized at these defect sites. These Pt_1_/Ti_3–*x*_C_2_T_*y*_ samples exhibited superior activity in the reductive fixation of CO_2_ via the formylation of amines to produce formamides (Figure 42).^113^ DFT calculations showed that compared to Pt NPs, single Pt atoms on Ti_3–*x*_C_2_T_*y*_ possess a partial positive charge, which leads to lower adsorption energies of silane, CO_2_, and aniline, and thus greater activity.^113^ This catalyst also demonstrated broad substrate scope for amide synthesis and could be reused without significant loss of activity.^113^

**Figure 42:**
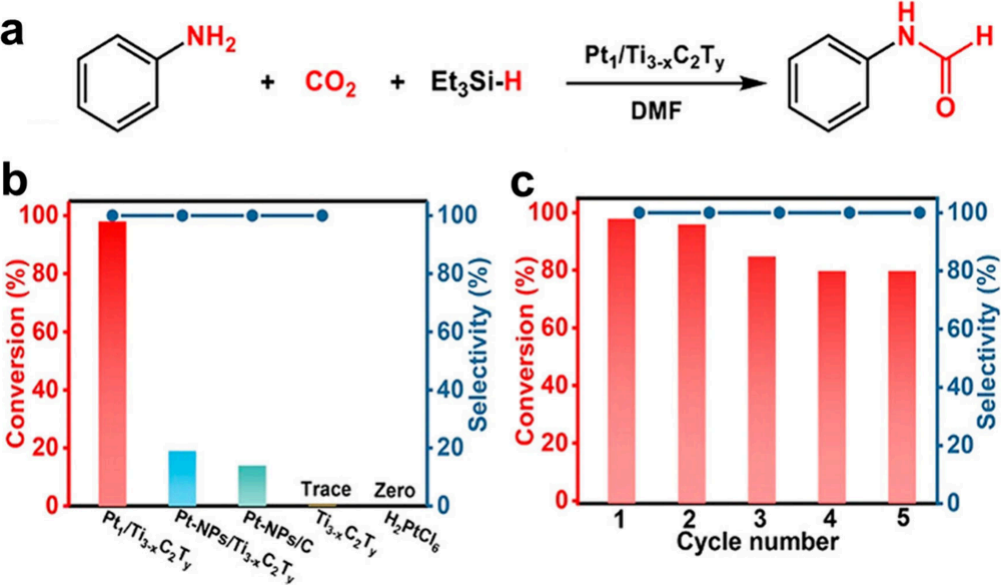
(a) Reaction of N-formylation of aniline by CO_2_ using Et_3_SiH as a reducing agent. (b) Catalytic performance of the N-formylation of aniline using different catalysts. (c) Recycling test of Pt_1_/Ti_3–*x*_C_2_T_*y*_ for the N-formylation of aniline. Reproduced with permission from ref. 113. Copyright 2019 American Chemical Society.

Taken together, the studies discussed in this subsection demonstrate that MXenes enable CO_2_ conversion and reforming through a combination of vacancy-stabilized single atoms, defect-activated surfaces, and strongly coupled MSIs. Their ability to regulate active-site dispersion, electronic structure, and surface chemistry underpins their versatility across diverse CO_2_ transformation pathways.

#### 5.3.5 N_2_ Fixation and NH_3_ Synthesis (Intrinsic MXenes and MXene-Supported NPs)

NH_3_ synthesis via N_2_ fixation is an industrially critical reaction and a long-standing challenge in heterogeneous catalysis. Unlike conventional Haber–Bosch catalysts, theoretical studies have suggested that surface-free MXenes can intrinsically activate N_2_ owing to their exposed transition-metal layers and unique electronic structures, offering alternative pathways for nitrogen activation under milder conditions.^192^ DFT calculations revealed that surface-free MXenes exhibit exothermic N_2_ adsorption with adsorption energies ranging from −1.11 to −3.45 eV and relatively low N≡N dissociation barriers, with values as low as 0.28 eV reported for W_2_N.^104^ Microkinetic simulations further indicated that NH_3_ formation is thermodynamically and kinetically feasible on selected MXene surfaces. However, strong N adsorption, slow hydrogenation steps, and difficult NH_3_ desorption may lead to surface poisoning, highlighting the challenge of achieving complete catalytic cycles using intrinsic MXenes alone.

Further insights were obtained from combined DFT and microkinetic modeling, which revealed a Brønsted–Evans–Polanyi (BEP) relationship^193^ between the N_2_ dissociation energy and the corresponding activation barrier on MXene surfaces (Figure 43).^104^ This relationship provides a predictive framework for screening MXenes with favorable N_2_ activation energetics and explains why nitride MXenes such as W_2_N exhibit particularly low dissociation barriers. While these theoretical results establish the fundamental capability of MXenes for N_2_ activation, they also emphasize the need for complementary strategies to facilitate hydrogenation and product desorption.^104^

**Figure 43:**
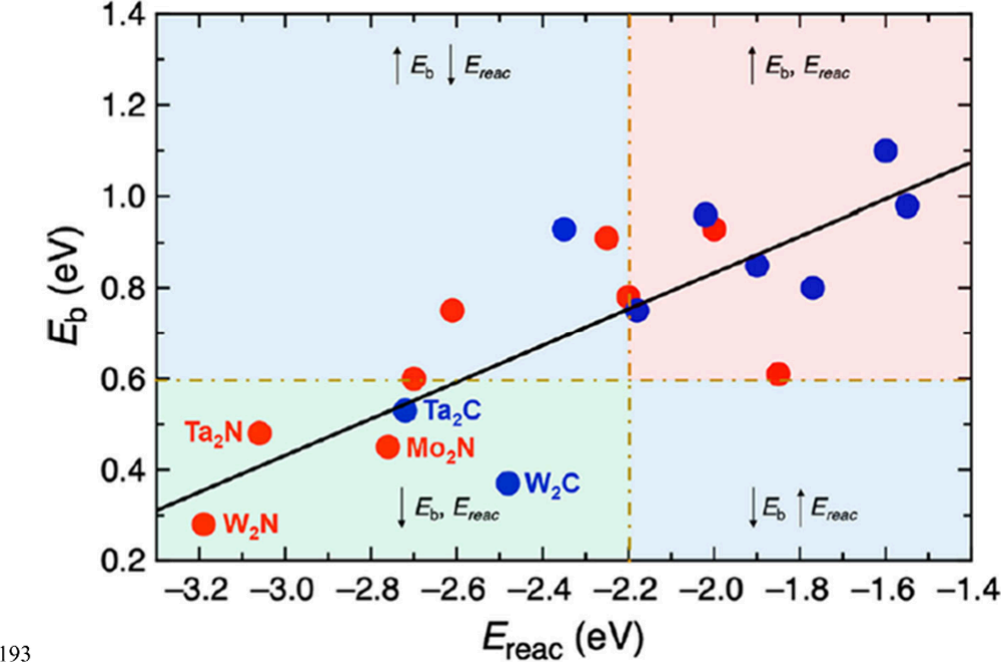
Plot of the BEP relationship for N_2_ dissociation reaction energy barrier, *E*_b_, and the reaction activation energy, *E*_reac_, on the MXene surfaces investigated; the black line corresponds to the linear regression of the calculated data, with equation *E*_b_ = 0.387*E*_reac_+1.611 and correlation coefficient *R* = 0.79. Blue and red dots correspond to data for the carbide and nitride MXenes, respectively. Reproduced with permission from ref. 104. Copyright 2020 American Chemical Society.

To overcome these limitations, MXenes have been employed as supports for metal NPs, combining the intrinsic N_2_ activation capability of MXene surfaces with efficient hydrogenation activity provided by the metal phase. Among reported systems, Co-decorated Mo_2_CT_*x*_ MXenes have demonstrated significant catalytic activity for NH_3_ synthesis under mild conditions.^106^ As shown in Figure 44, these catalysts exhibited measurable NH_3_ formation at temperatures as low as 250 °C and achieved an NH_3_ synthesis rate of 9 500 μmol g^–1^_active phase_ h^–1^ at 400 °C under ambient pressure, while maintaining stable performance for more than 15 days of continuous operation. Arrhenius analyses further confirmed the favorable kinetics of NH_3_ formation on MXene-supported Co catalysts. Table 4 compares the catalytic performance of various Co-containing Mo_2_CT_*x*_ MXenes and related materials.^106^

**Figure 44:**
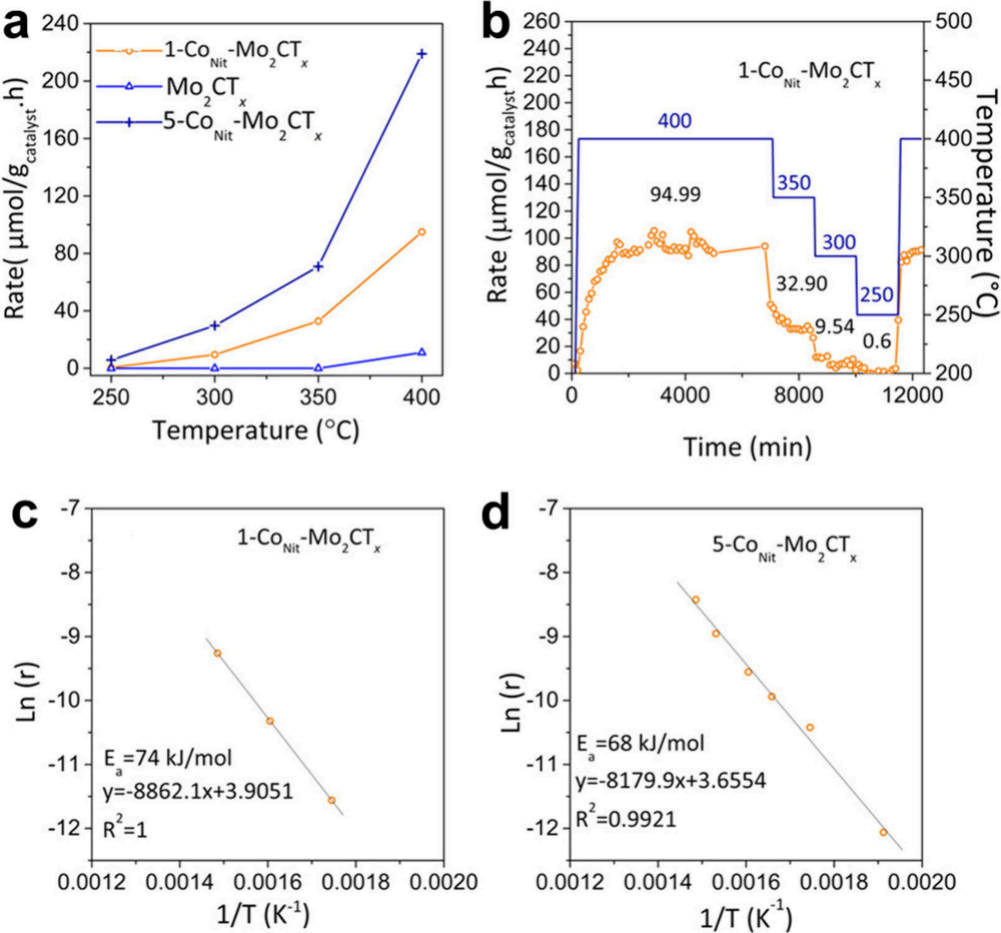
Catalytic activity of a series of Mo_2_C supported Co NPs catalysts in NH_3_ synthesis. (a) Temperature dependence of Mo_2_CT_*x*_, 1-CoNit-Mo_2_CT_*x*_, and 5-CoNit-Mo_2_CT_*x*_ (Nit referring to the use of cobalt nitrate for Co deposition) catalytic activity for NH_3_ synthesis. (b) Typical NH_3_ yield obtained of 1-Co_Nit_-Mo_2_CT_*x*_ catalyst at various temperatures, (c) Arrhenius plot obtained of 1-Co_Nit_-Mo_2_CT_*x*_ catalyst, and (d) Arrhenius plot obtained of 5-Co_Nit_-Mo_2_CT_*x*_ catalyst. The reaction was performed under 60 mL min^–1^ flow rate of 75 vol % H_2_/N_2_ at 400 °C and ambient pressure. Reproduced with permission from ref. 106. Copyright 2024 American Chemical Society.

**Table 4:** Activity and Kinetic Data of a Series of Mo_2_CT_*x*_ Catalysts^a^

Catalyst	NH_3_ production rate (μmol g^–1^ h^–1^)	NH_3_ production rate (μmol g_Co_^–1^ h^–1^)	Temperature (°C)	Activation energy^a^ (kJ mol^–1^)	Stability
Mo_2_CT_*x*_	23.2	ND	400	ND	Unstable
5-Co_Nit_-Mo_2_Ga_2_C	9.7	194	400	ND	Unstable
1-Co_Cl_-Mo_2_CT_*x*_	11	1102	400	ND	Unstable
1-Co_Nit_-Mo_2_CT_*x*_	95	9499	400	74	Stable
5-Co_Nit_-Mo_2_CT_*x*_	219	4380	400	68	Stable

a Reaction conditions: 60 mL min^–1^ of 75% H_2_ in N_2_ (99.98%) at 400 °C and atmospheric pressure. Data taken from ref. 106.

b ND corresponds to “not determined”.

Mechanistic investigations revealed that NH_3_ synthesis over Co/Mo_2_CT_*x*_ proceeds through a Mars–Van Krevelen-type mechanism, in which lattice nitrogen incorporated into the MXene framework directly participates in NH_3_ formation.^106^ Isotopic labeling experiments (^15^N/^14^N exchange), together with postreaction XPS and STEM-EDS analyses, confirmed the generation and consumption of lattice nitrogen during catalysis. In this dynamic process, hydrogenation of lattice N produces NH_3_ and leaves behind nitrogen vacancies that are subsequently replenished by gaseous N_2_, thereby sustaining a self-regenerating catalytic cycle. Operando XPS measurements further revealed partial reduction of Co^2+^ to Co^0^ and the emergence of a new N 1s feature (∼397.6 eV), assignable to metal–nitride species analogous to Co_3_Mo_3_N, providing direct experimental evidence for lattice-nitrogen participation.

Compared with bulk carbides such as such as α-Mo_2_C or Co_3_Mo_3_C, the 2D architecture of Mo_2_CT_*x*_ exposes a high fraction of reactive metal–carbon edges and provides short diffusion pathways for lattice nitrogen migration, thereby enhancing both the kinetics and reversibility of the catalytic cycle. These features highlight the unique role of MXenes as both conductive supports and dynamic nitrogen reservoirs in NH_3_ synthesis.^73^

In addition to Co-based systems, MXenes have also been explored as supports for other metal NPs through interfacial and functional design strategies. For example, a thermoresponsive MXene-based nanocomposite was constructed using Ti_3_C_2_T_*x*_ as the conductive support, Au NPs as the active species, and poly­(*N*-isopropylacrylamide) (PNIPAM) as a responsive matrix (Figure 45).^194^ The synthesis involved electrostatic adsorption of an azo compound (used as a radical initiator) onto Ti_3_C_2_T_*x*_, followed by NIPAM polymerization and subsequent “self-reduction” of Au ions on the Ti_3_C_2_T_*x*_ surface.^194^ Although not designed as a conventional Haber–Bosch-type catalyst, this system demonstrates how MXene-supported metal NPs can be integrated with functional matrices to enable dynamic regulation of catalytic behavior, including reversible on/off control via mild temperature modulation and facile catalyst separation. This example highlights the versatility of MXenes as supports that extend beyond static MSIs, enabling tunable interfacial environments and adaptive catalytic functions.

**Figure 45:**
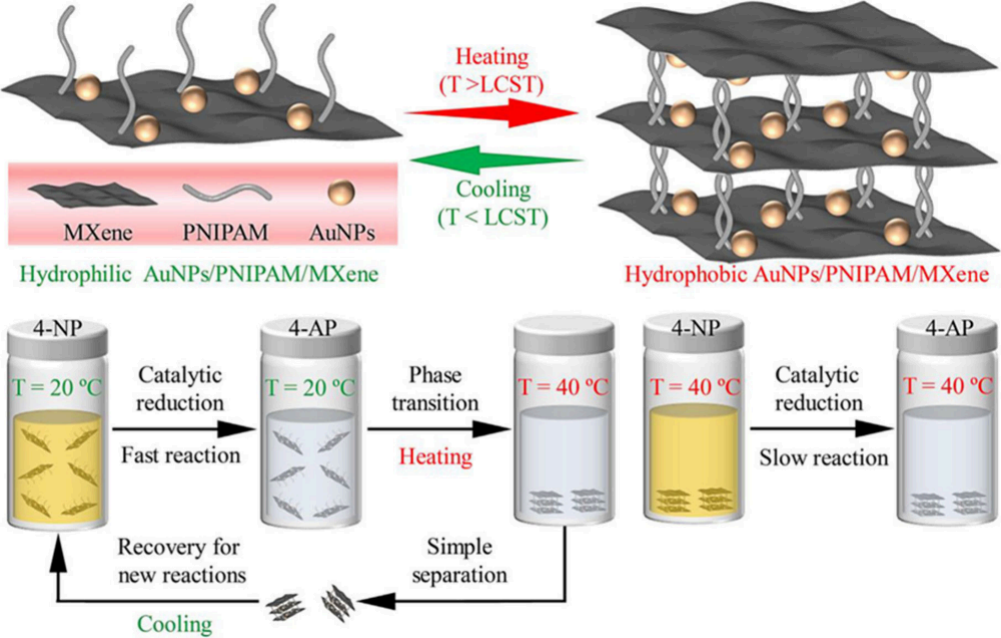
Schematic structure, thermoresponsiveness and operation as smart heterogeneous catalysis of AuNPs/PNIPAM/MXene nanocomposite. Reproduced with permission from ref. 194. Copyright 2022 Elsevier.

Overall, the combined theoretical and experimental studies demonstrate that MXenes offer a distinctive platform for N_2_ fixation and NH_3_ synthesis by integrating intrinsic nitrogen activation, lattice-nitrogen redox chemistry, and strong MSIs. While challenges related to surface poisoning and long-term durability remain, these findings underscore the potential of MXene-based catalysts to enable NH_3_ synthesis under milder conditions than traditional Haber–Bosch processes.

#### 5.3.6 Oxidation and Advanced Oxidation Processes (NPs and Vacancy-Anchored SACs)

Beyond hydrogenation- and reduction-dominated reactions, MXene-based materials have also been explored as supports for oxidation and advanced oxidation processes, where precise regulation of interfacial charge distribution, reactive oxygen species, and catalyst stability is critical.^195^ In these systems, the combination of strong MSIs and defect-rich MXene surfaces plays a decisive role in governing activity and selectivity. In conventional oxidation reactions, Pt-loaded Ti_3_C_2_ MXenes have been evaluated for benzene oxidation, revealing that charge transfer across the Pt–MXene interface strongly influences catalytic performance.^123^ This system is discussed further in Section 5.4 in the context of interfacial charge-density redistribution. More strikingly, Ti_3_C_2_ MXene has been employed as a nonoxide support for Pt NPs in room-temperature formaldehyde oxidation.^127^ After reductive pretreatment, a Pt_3_Ti intermetallic phase was formed as a consequence of strong MSIs, leading to markedly enhanced formaldehyde conversion even under high humidity conditions. *In-situ* DRIFTS measurements and DFT calculations revealed a stepwise oxidation mechanism involving formate (*HCOO) and CO intermediates, while Ti incorporation into Pt_3_Ti promoted oxygen activation and lowered the activation barrier for CO oxidation. These findings demonstrate how reactive MSIs on MXenes can generate intermetallic active phases that outperform monometallic Pt catalysts (Figure 46).^127^

**Figure 46:**
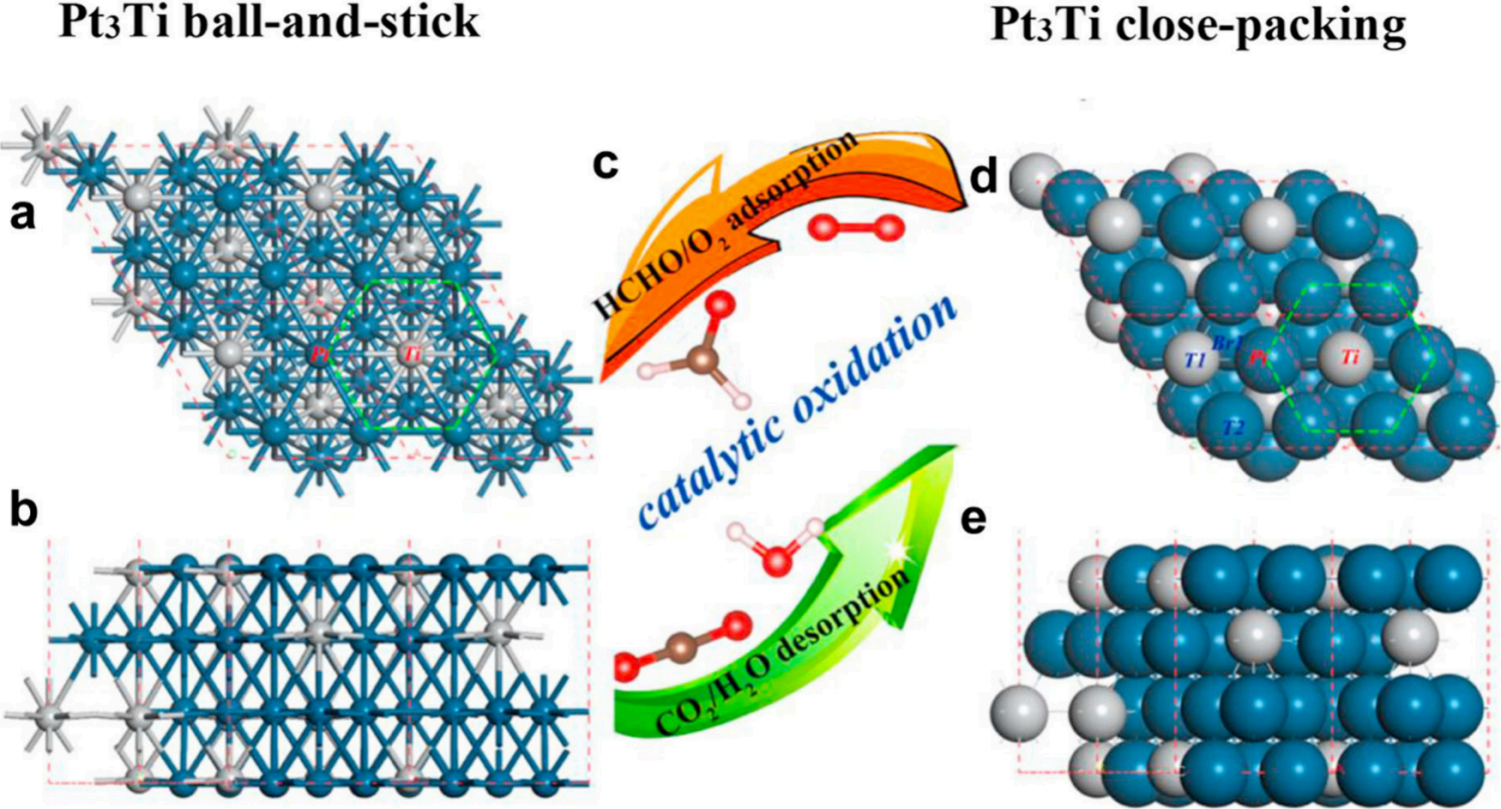
Optimized Pt_3_Ti structure responsible for formaldehyde oxidation. (a) Top view of Pt_3_Ti ball–stick model. (b) Side view of Pt_3_Ti ball–stick model. (c) Schematic of the catalytic formaldehyde oxidation reaction with the participation of O_2_. (d) Top view of Pt_3_Ti closely packed model. (e) Side view of Pt_3_Ti closely packed model. The red letters in panels (a) and (d) indicate the types of metal atoms. The part of the structure dotted in green in parts a and d represents the specific hexacoordinate structure of Pt_3_Ti. Blue and gray spheres represent Pt and Ti atoms, respectively. Reproduced with permission from ref. 127. Copyright 2023 Elsevier.

MXenes have also been extensively investigated in advanced oxidation processes for pollutant degradation, where vacancy engineering enables stabilization of isolated metal centers and selective generation of reactive oxygen species.^196,197^ For example, Ti-vacancy-rich Ti_2_N MXenes anchored with Co SAs exhibited rapid peroxymonosulfate activation, enabling efficient degradation of carbamazepine, sulfamethoxazole, and other organic pollutants within minutes.^114^ Quenching and EPR analyses indicated the involvement of both radical (^•^OH, SO_4_^•–^) and nonradical pathways. Similarly, single Cu atoms stabilized on Ti_3_C_2_T_*x*_ selectively generated singlet oxygen (^1^O_2_) during peroxymonosulfate activation, affording high activity and selectivity toward electron-rich contaminants such as sulfamethoxazole and tetracycline.^120^ These examples highlight the ability of MXene vacancies to stabilize isolated metal sites that favor specific oxidation pathways and controlled reactive species generation.

Overall, MXenes provide a distinctive platform for oxidation catalysis by integrating vacancy engineering, strong MSIs, and tunable interfacial electronic structures. These attributes enable both high catalytic efficiency and pathway selectivity, while simultaneously underscoring the importance of careful stability assessment under oxidative conditions, particularly with respect to MXene surface oxidation and metal leaching.

#### 5.3.7 Summary and Perspective for MXene-Supported SAs and NPs

As systematically summarized in Table 5, the studies discussed in Section 5.3 collectively establish MXenes as highly versatile and functionally active supports for both SAs and metal NPs across a broad spectrum of thermal catalytic reactions. Their intrinsic metallic conductivity, abundant surface terminations, and defect-rich transition-metal layers enable strong anchoring, efficient charge transfer, and precise electronic modulation of supported metal species. Across hydrogenation, dehydrogenation, CO_2_ conversion and reforming, N_2_ fixation, and oxidation reactions, a unifying theme emerges: MXenes do not merely disperse active metals but actively participate in determining reaction pathways, selectivity, and stability through MSIs, defect chemistry, and interfacial electronic effects. Vacancy-anchored SAs, intermetallic interfaces, and lattice-mediated redox mechanisms repeatedly appear as key motifs, fundamentally distinguishing MXenes from conventional oxide or carbon supports.

**Table 5:** Representative Roles of MXenes as Supports for SAs and Metal NPs across Different Thermal Catalytic Reactions

Reaction class	Active metal species	MXene support	Dominant anchoring/interfacial feature	Key catalytic role of MXene	Representative outcome
Hydrogenation/reductions	Pt, Pd SAs; Pd, Ru, Pt NPs/NCs	Ti_3_C_2_T_*x*_, Nb_2_C	Vacancy trapping; strong MSI; electron donation from MXene	Stabilization of isolated metal sites; tuning adsorption strength and suppressing overhydrogenation	High selectivity in semihydrogenation and nitroaromatic reduction; enhanced TOF and durability
Dehydrogenation	Rh_2_ dual atoms; RhNi, PdCr, Pt NPs; Pt nanolayers	V_2_CO_2_, Ti_3_C_2_T_*x*_, Mo_2_TiC_2_	Dual-atom stabilization; alloy formation; intermetallic interfaces	Lowering C–H activation barriers; enhanced resistance to coking	High activity and stability in ethane/propane dehydrogenation and hydrogen carrier reactions
CO_2_ conversion and reforming	Bi, Ni SAs; Cu, Co, Ni NPs	V_2_C, Ti_3_C_2_O_2_, Mo_2_CT_*x*_, V_2_C-derived MXenes	Vacancy-anchored SAs; defect-assisted adsorption; reactive MSI	Selective CO_2_ activation; stabilization of Cu^+^/metal–carbide interfaces; suppression of coke formation	High selectivity toward HCOOH, CH_3_OH, CO or CH_4_; stable DRM performance
N_2_ fixation and NH_3_ synthesis	Intrinsic MXene sites; Co NPs	Mo_2_CT_*x*_, surface-free MXenes	Lattice-N participation; Mars–Van Krevelen mechanism; strong MSI	Direct N_2_ activation; coupling of N activation with efficient hydrogenation	NH_3_ synthesis under milder conditions with long-term stability
Oxidation reactions	Pt NPs; Co, Cu SAs	Ti_3_C_2_T_*x*_, Ti_2_N	Intermetallic formation; vacancy-anchored SACs	Enhanced oxygen activation; controlled ROS generation	Complete formaldehyde oxidation; efficient PMS activation
Advanced oxidation processes	Co, Cu SAs	Ti_2_N, Ti_3_C_2_T_*x*_	Metal–vacancy coordination; selective PMS activation	Preferential generation of ^1^O_2_ or radical/nonradical pathways	Rapid and selective degradation of organic pollutants

At the same time, the sensitivity of MXenes to surface oxidation, potential metal leaching, and structural evolution under harsh reaction conditions highlights the need for systematic durability evaluation and operando characterization. Future efforts should focus on correlating termination chemistry, defect density, and interfacial structure with long-term catalytic performance. Overall, the unique combination of structural tunability, electronic flexibility, and interfacial reactivity positions MXenes as a powerful and adaptable platform for next-generation heterogeneous catalysis based on SAs and nanostructured metal species.

### 5.4 Charge/Thermal Transport-Assisted Reactions

VOCs are hazardous pollutants in air and water that pose serious health risks. These compounds are emitted from industrial processes, transportation, disposed wastewaters, and household products, potentially leading upon chronic exposure to significant health problems and environmental concerns such as smog formation, respiratory diseases, and carcinogenic effects.^198^

Catalytic degradation is a highly effective method for mitigating VOC pollution by converting harmful emissions into nontoxic byproducts, very frequently targeting the mineralization of organic compounds to CO_2_ and H_2_O. Various approaches have been developed to optimize catalytic VOC removal efficiency. In the most common one, thermal catalysis is used to activate metal-based catalysts, promoting VOC oxidation into harmless compounds.^199^ Photothermal catalysis enhances this process by using light to assist and increase the efficiency of the reaction, augmenting reaction rates and improving degradation outcomes.^200^ Very frequently, the VOC degradation pathway is promoted through a Fenton-like advanced oxidation process that uses an oxidizing reagent to generate reactive oxygen species, which actively attack VOCs, initiating oxidative degradation assisted by atmospheric oxygen.^201^ These catalytic strategies offer effective solutions for VOC emission abatement in industrial emissions and environmental remediation applications.

Catalytic oxidation is a process used for the destruction of pollutants, such as VOCs and hazardous air pollutants, by using heat and catalysts to facilitate oxidation. It is commonly used in air pollution control^202^ and can also be applied to the treatment of wastewater and soils.^203^ In this context, it is well documented that MXenes activate hydrogen peroxide decomposition, acting as efficient catalysts to generate reactive oxygen species capable of triggering pollutant oxidative degradation. In Fenton-like processes, MXenes can facilitate the activation of H_2_O_2_ to produce highly reactive ^•^OH (Scheme 2), leading to the rapid oxidation of organic contaminants. Catalytic Fenton reactions can also be applied to wastewater treatment. MXenes have shown great potential to promote advanced oxidations and hydrocarbon oxidations. In air purification treatment, MXene-based catalysts demonstrate high efficiency in breaking down VOCs, such as formaldehyde^127^ and benzene,^123^ at moderate temperatures. The tunable surface functional groups of MXenes and the presence of redox-active transition metals in their structure make them well suited for this type of catalysis, providing an alternative to the conventional stoichiometric Fe^2+^-based Fenton reaction under acidic conditions. One major challenge is to prove MXene stability and reusability, and to elucidate the mechanism of the advanced oxidation process, particularly the key reactive oxygen species responsible for degradation.^204^ Certainly, a challenge in MXene catalysis will be to develop materials that can withstand oxidative environments and to delineate design strategies for their synthesis.

MXenes serve as effective catalytic supports for metal species, in which the local environment around the metal is typically considered. However, collective properties of MXenes, such as work function, electron density, and mobility, can also contribute to catalytic activity by enabling charge transfer between the MXene and the supported metal. This interfacial electron density transfer enhances MSIs and favors catalytic processes in which charge density transfer plays an important role, such as oxidations. In one example of using metal-containing MXenes as catalysts for pollutant abatement, Pt-doped Ti_3_C_2_ MXene (Pt@Ti_3_C_2_) was employed for benzene oxidation that is a relevant probe reaction for the treatment of aromatic air pollutants. The catalyst exhibited a significant increase in oxidation efficiency after a high-temperature H_2_ reduction pretreatment, which enhances the MSI and promotes oxygen insertion at catalytic sites. Notably, a 1% Pt@Ti_3_C_2_-R catalyst (where “R” indicates reduction pretreatment) achieved a benzene oxidation rate of 0.012 mol g^–1^ h^–1^ at 200 °C, significantly outperforming untreated MXene catalysts (Figure 47).^123^ The high-temperature H_2_ reduction pretreatment significantly enhances the catalytic performance of Pt@Ti_3_C_2_ MXene catalysts by optimizing the structural and electronic properties of Pt NPs. It strengthens the interaction between Pt NPs and the MXene support, facilitating efficient electron density distribution at the Pt–MXene interface. Additionally, it increases the availability of lattice oxygen (O*), a crucial factor for oxidation reactions, as evidenced by XPS analysis, which showed an increase in lattice oxygen content from 45.3% to 71% after reduction. Furthermore, pretreatment diminishes the proportion of adsorbed oxygen (O_ads_), ensuring that more reactive oxygen species are available for catalysis, thereby improving the overall oxidation efficiency. The oxidation of benzene over MXene-based catalysts follows a multistep mechanism involving adsorption, activation, and complete oxidation. Initially, benzene molecules adsorb onto the catalyst surface, particularly at Pt sites and oxygen vacancies. These adsorbed molecules interact with lattice oxygen (O*) and vacancies, forming intermediate species such as phenolate and benzoquinone. Subsequent oxidation of phenolate results in the cleavage of the benzene ring, generating smaller intermediates like formate. Finally, continued attack of O* on these intermediates facilitates complete oxidation into CO_2_ and H_2_O. Theoretical calculations further support this mechanism, demonstrating that the apparent activation energy (*E*_app_) decreases significantly for the reduced catalysts, suggesting faster reaction kinetics. Additionally, a positive entropy change (Δ*S*‡ = 16.3 ± 9.8 kJ mol^–1^ K^–1^) suggests a dissociative oxidation mechanism, in which highly mobile oxygen species enhance reactivity. Moreover, the reduction pretreatment increases the oxygen storage capacity from 4.13 × 10^–3^ to 4.84 × 10^–3^ mol g^–1^ for 1% Pt@Ti_3_C_2_-R, further improving the catalyst’s efficiency in benzene oxidation.

**Figure 47:**
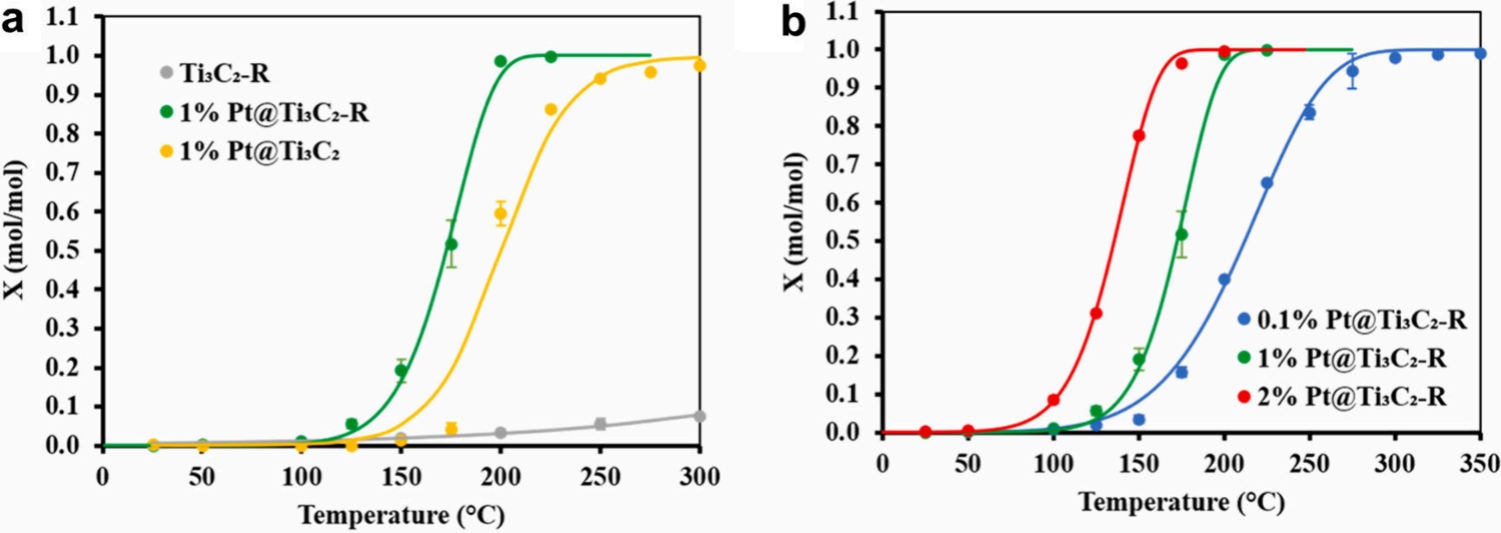
Curves for the benzene conversion performance of (10 ppm in dry air and RH = 0%): (a) comparison between the catalysts of the control group (bed mass: 10 mg catalyst +50 mg sand and FR: 50 mL min^–1^) and (b) effect of different Pt loading (bed mass: 10 mg catalyst + 50 mg sand and FR: 50 mL min^–1^) on Pt@Ti_3_C_2_-R. Reproduced with permission from ref. 123. Copyright 2024 Elsevier.

As has been commented, Ti_3_C_2_ can serve as an active support by establishing strong reactive MSIs, leading to the formation of intermetallic compounds such as Pt_3_Ti. The strong M–C bonds in MXenes allow for the formation of stable intermetallic active sites, while the surface functional groups enhance adsorption and activation of reactants. The presence of −OH and −O groups on the surface contributes to the catalytic activity by enhancing metal dispersion and tuning the work function and electronic interactions. It has already been discussed that reduction pretreatment of 2% Pt/Ti_3_C_2_ led to the formation of intermetallic Pt_3_Ti, which displayed superior catalytic activity for benzene oxidation. The treatment with H_2_ at 300 °C caused Pt NPs to merge, forming a homogeneous intermetallic structure. This modification improved the electronic properties of the catalyst and facilitated O_2_ activation, enhancing the oxidation process. The oxidation mechanism described in these studies relies on the activation of lattice oxygen (O*) and surface oxygen vacancies. These O* are continuously replenished by molecular O_2_ from the reaction atmosphere. This cycle is in accordance with the Mars–Van Krevelen mechanism, where oxidation occurs through the direct involvement of lattice oxygen, followed by its regeneration from gaseous oxygen. In thermocatalytic oxidation studies involving MXenes, O* plays a pivotal role in VOC oxidation, while oxygen vacancies act as essential sites for sustaining the reaction by facilitating oxygen mobility and maintaining catalytic activity.

MXenes are also highly efficient catalysts for wastewater treatment, particularly in Fenton-like advanced oxidation processes. They can activate hydrogen peroxide, persulfates, and can absorb light to generate reactive radicals for pollutant degradation.^205^

The catalytic performance of MXene-based materials in advanced oxidation processes depends on several factors. Solution pH influences efficiency, with MXenes working across a broad acidic pH range is required for conventional Fenton-like reactions. Catalyst and oxidant concentrations must be optimized; while higher levels improve degradation, they might cause radical quenching or unwanted side reactions. Temperature is also a crucial factor, as it accelerates reactions but may raise costs and affect the catalyst stability. MXene reusability is challenged by oxidation, though modifications can enhance longevity. Finally, toxicity assessment is essential to ensure that the breakdown products of pollutants do not pose their own environmental or health risks.^204^ A comprehensive understanding and optimization of these factors is essential for maximizing the effectiveness of MXene-based advanced oxidation processes in wastewater treatment.

MXenes activate hydrogen peroxide (H_2_O_2_) to generate ^•^OH for pollutant degradation. They also activate persulfates (APS, PMS, or peroxydisulfate) to generate ^•^OH and/or sulfate radicals (SO_4_^**·**–^), with the advantage that for these sulfate reagents, the radicals can be formed at neutral pH values.^114,120^

Multilayered Ti_3_C_2_T_*x*_ MXene (ML-Ti_3_C_2_T_*x*_) demonstrated exceptional catalytic efficiency, achieving 100% degradation of methylene blue within 24 min under optimized conditions. The reaction followed pseudo-first-order kinetics, with ^•^OH identified as the primary active species responsible for dye degradation.^206^ The mechanism involved H_2_O_2_ activation by low-valence titanium species (Ti^2+^ and Ti^3+^) present in MXene, which, by electron donation to H_2_O_2_, generate highly oxidizing ^•^OH. Ti^2+^ first reacted with H_2_O_2_, forming Ti^3+^ and initiating the radical generation process. Ti^3+^ becomes further oxidized to Ti^4+^, producing additional ^•^OH radicals.^207^ As it has been illustrated in Scheme 2, H_2_O_2_ can also act as a reducing agent, forming O_2_ and returning some high-valence Ti^4+/3+^ to lower Ti^2+^ oxidation states. This efficient and sustainable reaction pathway highlights the catalytic activity of MXenes in Fenton-like catalysis by providing abundant active sites for H_2_O_2_ activation. Unlike conventional Fe^2+^-based catalysts, MXene functions effectively across a broad pH range, maintaining high degradation efficiency without requiring heterostructures or light irradiation. Additionally, its accordion-like layered structure enhances dye adsorption, further improving catalytic performance.

However, MXene stability is an issue of high concern. In fact, it has also been reported that multilayered Ti_3_C_2_T_*x*_ MXene (ML-Ti_3_C_2_T_*x*_) oxidizes in the presence of H_2_O_2_. This occurs as low-valence Ti species (Ti^2+^ and Ti^3+^) react with H_2_O_2_, converting into Ti^4+^, which is only sluggishly reduced to Ti^2+^ and mostly hydrolyzed to TiO_2_. Accordingly, in this process, ML-Ti_3_C_2_T_*x*_ should be seen as a stoichiometric reagent rather than a catalyst. The TON is the key metric to assess whether the process should be considered catalytic or stoichiometric. Unfortunately, under the experimental conditions in which 10 mg of ML-Ti_3_C_2_T_*x*_ is used to react with between 5 and 20 mg of pollutant, and without knowing the exact products and degree of mineralization, it is not possible to determine if all the Ti present in the catalyst is responsible for the stoichiometric methylene blue degradation. However, the observed formation of TiO_2_ is a clear sign of instability.

On a positive note, mild oxidation of MXenes with H_2_O_2_ can be considered an appropriate procedure to obtain MXene-derived 2D materials and composites, in which MXenes act as both a structural template and a multivalent metal precursor, offering a universal strategy for designing a wide range of materials that could be difficult to obtain through other methods.^97^

Thus, when mildly oxidized with H_2_O_2_, exfoliated Ti_3_C_2_ MXene transforms into titanium oxide NCs on a carbon backbone (TiO_1.47_@C), the resulting MXene-derived material exhibiting excellent Fenton-like catalytic activity (Figure 48).^97^ This catalyst efficiently degraded atrazine, a widely used herbicide, within 5 min under optimized conditions, utilizing 5 mM H_2_O_2_ and 0.1 g/L Ti across a broad pH range (3–11). This broad range of pH values is remarkable, since acidic pH values below 5 are normally required to generate ^•^OH from H_2_O_2_. At basic pH values starting in 8, peroxyl radicals (^•^OOH), with much milder oxidizing properties, which are generally insufficient for mineralization, are formed.

**Figure 48:**
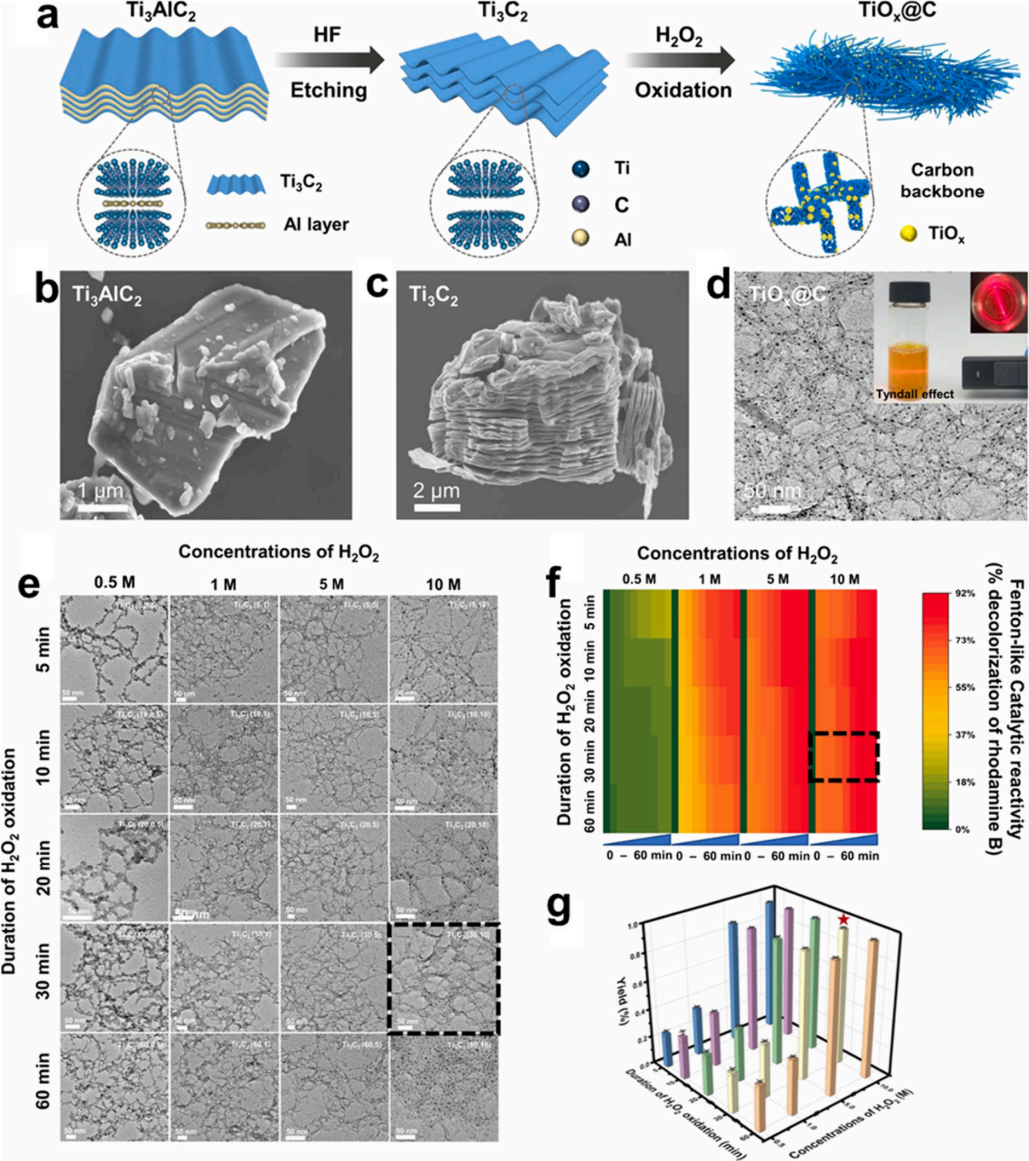
Synthesis and characterizations of TiO_*x*_@C catalyst. (a) Schematic description of formation of TiO_*x*_@C templated by exfoliated Ti_3_C_2_ MXene. SEM images of bulk Ti_3_AlC_2_ (b) and Ti_3_C_2_ (c) displaying a typical accordion-like structure after HF etching. (d) TEM image of TiO_*x*_@C with TiO_*x*_ NCs (dark dots) decorated on an amorphous carbon backbone (light gray). Inset: photograph of TiO_*x*_@C aqueous dispersion showing the typical Tyndall effect. (e) TEM images of TiO_*x*_@C fabricated by increasing concentrations of H_2_O_2_ (0.5, 1, 5, and 10 M) and duration of oxidation (5, 10, 20, 30, and 60 min). (f) Contour map of the Fenton-like catalytic performance of TiO_*x*_@C based on decolorization of rhodamine B. Red to green indicates high to low activities. (g) Yield (%) of TiO_*x*_@C from oxidation of Ti_3_C_2_ by variations of H_2_O_2_ concentrations and duration of oxidation. Reproduced with permission from ref. 97. Copyright 2023 National Academy of Sciences.

DFT calculations confirmed that these Ti-deficient sites were the primary reaction centers, ensuring efficient electron transfer. Ti-deficit vacancies in MXenes act as reactive sites, where multivalent Ti^2+^, Ti^3+^, and Ti^4+^ species facilitated H_2_O_2_ activation, leading to the generation of ^•^OH and superoxide radicals, which oxidize toxic herbicides and pesticides into smaller, nontoxic byproducts. Again, a careful evaluation of the number of turnover cycles should be conducted to assess the stability of TiO_1.47_@C as a catalyst.

Single metal atoms on MXenes (SA/MXene) have also been tested as catalysts in advanced oxidation processes. In one example, a Cu-SA/MXene system was used for the activation of PMS (HSO_5_^–^). The reaction was found to follow a selective nonradical oxidation pathway with the generation of ^1^O_2_, without the formation of sulfate (SO_4_^•–^) and ^•^OH radicals. PMS selectively binds to Cu-SA/MXene through its terminal oxygen, initiating the activation process. This interaction facilitates electron transfer from PMS to the Cu sites, leading to the formation of the intermediate SO_5_^•–^. Subsequently, SO_5_^•–^ undergoes self-reaction, generating ^1^O_2_ along with persulfate (S_2_O_8_^2–^). The MXene support plays a crucial role in stabilizing Cu SAs, which act as active sites, ensuring a remarkably high selectivity (∼99.71%) for ^1^O_2_ generation, making the system highly efficient for pollutant degradation.^120^

It has been proposed that MXenes hold great potential for industrial wastewater treatment, air purification, and soil remediation. However, concerns about their long-term environmental impact, potential toxicity, and material stability remain issues that require careful assessment. In order to fully exploit the potential of MXenes for environmental remediation and other areas, general challenges such as greener synthesis methods and large-scale implementation should be addressed.

In summary, MXenes exhibit great potential in charge- and thermal transport-assisted catalytic processes, particularly in environmental applications such as VOC degradation and advanced oxidation. Their high electrical and thermal conductivity, tunable surface terminations, and ability to activate oxidants like H_2_O_2_ and PMS enable the generation of reactive oxygen species (^•^OH, ^•^O_2_^–^, ^1^O_2_) under mild conditions. These properties make MXenes effective for both thermocatalytic and Fenton-like reactions across a broad pH range, offering an advantage over traditional catalysts. Furthermore, the strong MSIs and interfacial electronic modulation in MXene-supported systems enhance catalytic performance, especially in oxidation reactions. However, challenges remain regarding the long-term stability of MXenes under oxidative conditions and the need to quantify true catalytic TON values. Addressing these limitations is essential for advancing MXene-based materials toward practical applications in pollution control and sustainable catalysis.

## 6 Photothermal Catalysis with MXenes: Applications and Mechanisms

### 6.1 Fundamentals of Photothermal Effect in MXenes

Thermal catalysis overcomes the activation energy barrier of a reaction using heat. This heat is generally obtained in industrial processes by burning fossil fuels and is supplied to the system mainly through conduction, convention, and diffusion from the walls of the reactor to the active sites, or by heating reagents. In addition to fuel combustion, there are also many other ways to generate heat, including the Joule effect for direct electricity-to-heat conversion,^208^ magnetic induction,^208^ and microwaves.^209^ Related to microwaves heating, other forms of electromagnetic radiation, particularly in the IR region, but also in the visible spectrum, can result in heating via local temperature increase.^210^ Figure 49 illustrates materials, mechanisms, and applications of the photothermal effect.

**Figure 49:**
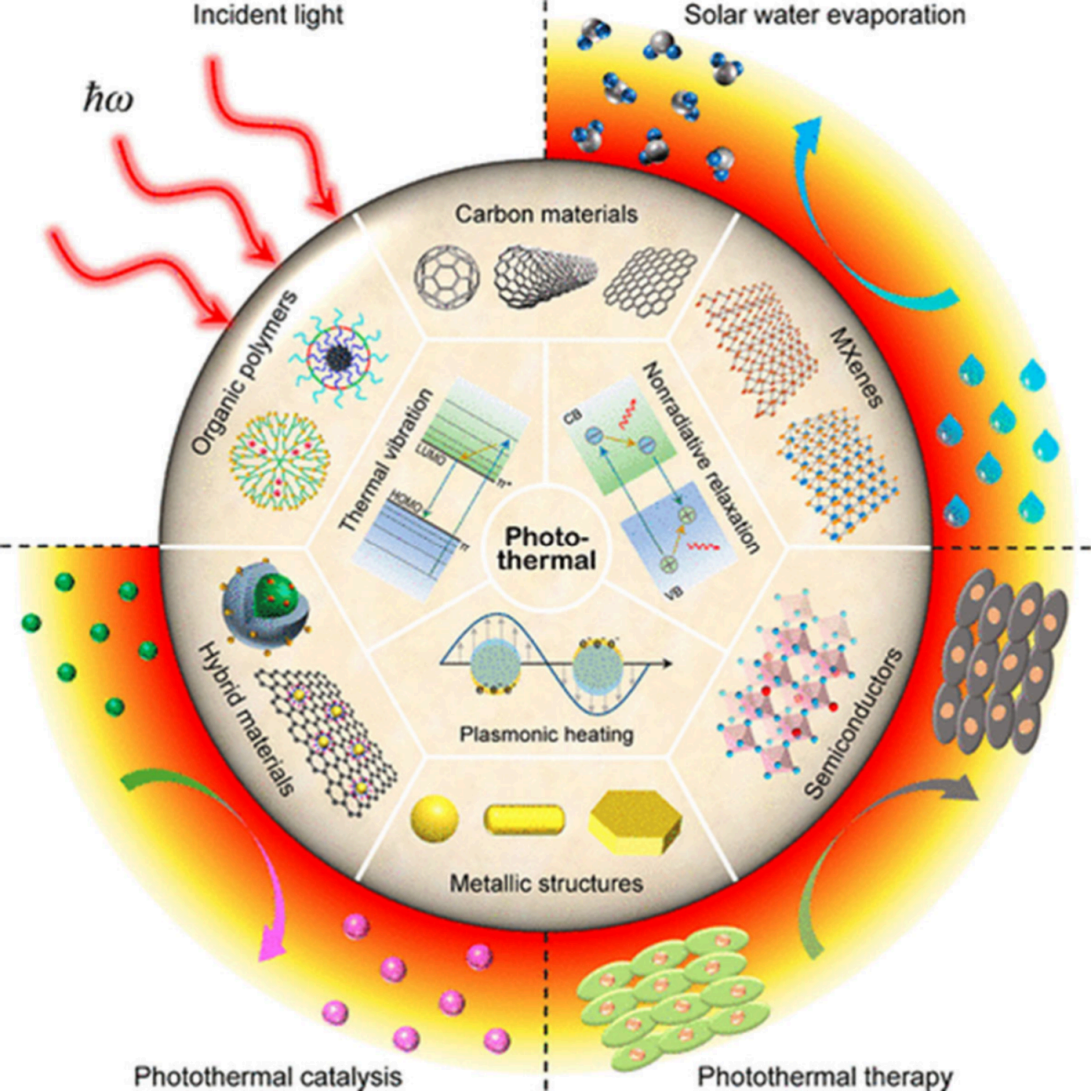
Illustration summarizing the mechanisms, materials, and applications of photothermal processes. Reproduced with permission from ref. 210. Copyright 2023 American Chemical Society.

Unique features of light-based heating include temporal and spatial resolution, as light can be delivered as short laser pulses in which the power, defined as energy divided by time, can be enormously high due to ultrashort pulse durations. Additionally, light beams can be focused on specific zones while leaving others in the dark. However, regardless of these peculiarities of heating with light, this method of promoting thermal reactions using photons has opened a new field of light-assisted catalysis, generally referred to as photothermal reactions.^139,210^ In photothermal reactions,^211^ the reaction mechanism can be essentially the same as that in conventional thermal catalysis and can involve the same reaction intermediates. This also applies for SAs and catalysis by metal NPs combined with photothermal catalysis.^211–214^ However, photothermal reactions typically occur at lower temperatures than in thermal catalysis, suggesting that hot electrons contribute to the process to some extent.^215^ Within this context, and considering the properties of MXenes as efficient photoabsorbers and rapid thermalizers, it is not surprising that these materials have attracted growing attention as photothermal catalysts.

#### 6.1.1 Light-to-Heat Conversion Mechanisms

Upon irradiation, heat evolves due to the thermalization of photon energy. When light in the visible-Near IR region is absorbed, valence band electrons are excited to higher electronic states.^210^ Relaxation can occur through various deactivation mechanisms, but when it proceeds via electron–phonon coupling, it leads to the transfer of light energy to the lattice of the material, altering the vibration of lattice atoms and bonds. This transfer from electronic to lattice energy corresponds to the conversion of light energy into heat, resulting in a local temperature increase. Figure 50 illustrates the process of converting photon energy into heat.

**Figure 50:**
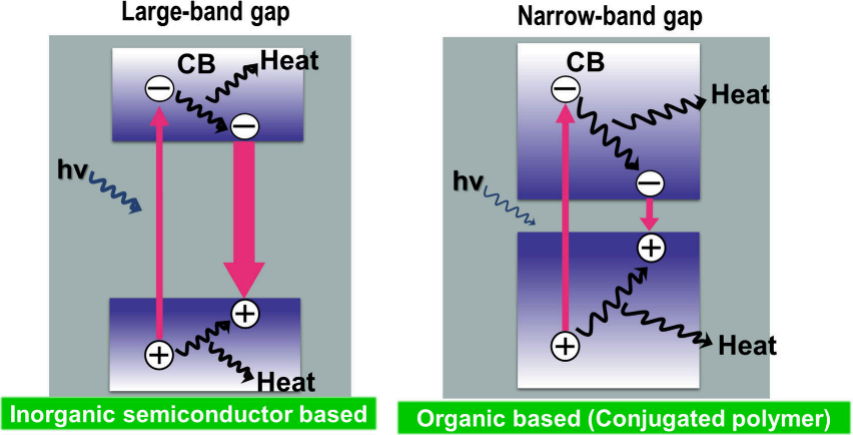
Illustration of the conversion of a photon into heat through coupling electron excitation with energy relaxation by phonon coupling. Adapted from ref. 216.

This localized heating occurs initially at the site of photon absorption, and the generation of phonons takes place on a picosecond time scale. Over longer time scales, heat can dissipate to the surroundings, depending on the material’s thermal conductivity and contact with solvents or gases.^210^ Under continuous irradiation, a temperature gradient may be established in the material, with illuminated areas being hotter than dark ones and the appearance of localized hot spots at the micrometer scale. This temperature distribution can be captured by thermal IR cameras; however, it also highlights that a single temperature value, such as that obtained using conventional thermocouples, does not accurately reflect the heterogeneous heat distribution occurring during photothermal processes.

Considering that metal NPs exhibit plasmon absorption bands spanning a broad region of the visible to IR spectrum, light-induced heating offers an efficient way to utilize energy. Since photons are absorbed at the metal NPs, which often serve as the active sites of the catalyst, heat is generated precisely where the reaction occurs, without wasting energy on heating reactor walls or other nonreactive components. This localized energy delivery can lead to higher energy efficiency in photothermal reactions compared to conventional heating, in a manner similar to that observed with microwave or magnetic induction heating. It is important to note that reported photothermal efficiencies strongly depend on the definition of efficiency and the experimental configuration. In particular, internal light-to-heat conversion efficiency refers to the fraction of absorbed photon energy dissipated as heat, whereas system-level or solar-to-thermal efficiencies additionally account for optical losses, heat dissipation, and device architecture.

#### 6.1.2 Factors Affecting Photothermal Effects: Surface Plasmon, Bandgap, Structure

As already discussed, MXenes frequently exhibit metallic character, showing neutral absorption in the visible spectrum but displaying a UV band attributed to ligand-to-metal *M–T* electronic transitions. In addition, the optical absorption spectrum of MXenes commonly exhibits a plasmon band at the red end of the visible region.^141,217^ Figure 51 presents a typical UV–Vis spectrum of MXene.

**Figure 51:**
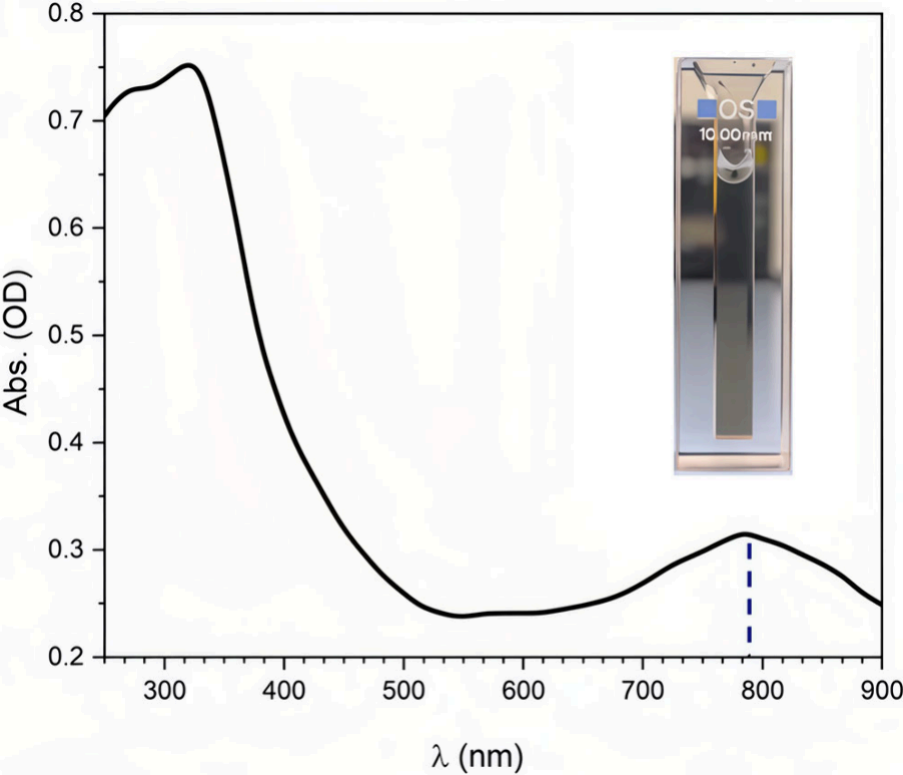
UV–Vis spectrum of Ti_3_C_2_ obtained by fluoride etching from Ti_3_AlC_2_. The inset shows the visual appearance of the Ti_3_C_2_ suspension. Reproduced with permission from ref. 218. Copyright 2023 MDPI.

The metallic character of MXenes accounts for their typically narrow bandgap, although the exact value is influenced by the nature of surface terminal groups and defects.^102^ Simply put, oxygen-containing functional groups make MXenes behave more like metal oxide semiconductors, and such surface terminations can open a bandgap, as evidenced by the UV absorption features in the optical spectrum.

All these properties make MXenes particularly well suited as light absorbers and thermalizers,^219,220^ effectively converting photons into heat. Under certain experimental conditions, near-unity internal light-to-heat conversion efficiencies have been reported for MXene dispersions under laser irradiation, reflecting the highly efficient nonradiative dissipation of absorbed photon energy. However, such values correspond to material-level internal efficiencies measured under localized and confined illumination conditions, rather than device-level solar-to-thermal efficiencies under standard solar irradiation.^220^ Furthermore, when MXenes are used as support for metal NPs, the resulting hybrid structures can couple photothermal heat generation with the catalytic activity of the metal species. Besides metals as NPs or metallenes on top of the MXene surface, MXenes can also form heterostructures with other 2D nanomaterials, such as graphene-like carbons or 2D metal–organic frameworks (MOFs) (see Table 1) that combine the light absorption, thermal conductivity and thermalization properties intrinsic to MXenes with the catalytic activity of the other material. The 2D morphology and the strong interfacial interaction make MXenes very suitable components for these heterojunctions in photothermal reactions. In most cases, the *in situ* growth of the components on pre-existing MXene results in better performance due to the favorable interfacial contact. Most reported applications of MXenes as photothermal catalysts fall into this category. However, as outlined in earlier sections, an underexplored area is the utilization of inherent structural active sites of MXenes in light-driven thermal reactions. The following sections will briefly review reported examples of MXene-supported metal NPs used in photothermal CO_2_ reduction, hydrogen evolution and N_2_ fixation, underscoring the need for further investigation to unlock the full catalytic potential of MXenes in this domain.

### 6.2 CO_2_ Photoreduction: Key Materials, Active Intermediates, Proposed Mechanisms

The conversion of CO_2_ into fuels and feedstock chemicals via photothermal catalysis holds promise for efficient solar energy utilization to mitigate atmospheric CO_2_ emissions and climate change.^221^ In this regard, a key research focus is the development of emerging materials with excellent photothermal properties to enhance the performance of photothermal CO_2_ catalysts.^222^ In one representative study, Ni NPs were deposited on Nb_2_C and Ti_3_C_2_ MXenes to reinforce the photothermal effect through synergistic interactions between the two components.^223^ Negligible photocatalytic activity was observed at 400 °C under 13-sun irradiation using Nb_2_C alone. Surprisingly, a CO_2_ conversion rate of 8.50 mol g_Ni_^–1^ h^–1^ was achieved using Ni/Nb_2_C-nanosheets with 36-sun illumination at 400 °C (Table 6). Furthermore, the activity of Ni/Nb_2_C was 6.3 times higher than that of Ni/Nb_2_O_5_ with an identical Ni particle size (9.1 ± 2.4 nm) under similar photothermal CO_2_ hydrogenation conditions. A comparable enhancement was observed when Ni NPs were supported on Ti_3_C_2_, demonstrating the broader applicability of this strategy. This study clearly illustrates how thermally conductive MXene materials can cooperate with metal NPs as active sites to enhance photothermal CO_2_ catalysis, enabling more efficient solar-to-chemical energy conversion. Continuing in this direction, Ru NPs supported on Mo_2_TiC_2_ were employed as a photothermal catalyst for the reverse water–gas shift reaction, achieving an activity of 4.0 mol g_Ru_^–1^ h^–1^, that is among the highest reported to date.^224^

**Table 6:** Properties and Photochemical Catalytic Performance of Ni/Nb_2_C and Ni/Nb_2_O_5_. Data Taken from ref. 223

Sample	Loading amount of Ni^a^ (wt %)	Size of Ni^b^ (nm)	R_CO2_ ^c^ (mmol g^–1^ h^–1^)	CH_4_ rate (mmol g^–1^ h^–1^)	CH_4_ selectivity (%)
Ni/Nb_2_C	6.0	9.0	87.0	72.5	83.4
Ni/Nb_2_O_5_	6.9	9.1	15.9	12.9	80.9

a Determined by ICP-MS.

b Obtained by measuring 100 Ni NPs from TEM images.

c CO_2_ conversion rate in the batch reactor under 15-sun illumination at 400 °C for 20 min.

In another report, an S-vulcanized Ti_3_C_2_ MXene photocatalyst (S-Ti_3_C_2_) containing hollow TiS_2–*x*_O_*x*_ NPs was prepared by hydrothermal treatment of Ti_3_C_2_ with thiourea in an ethanol/ethylene glycol 9/1 mixture at 150 °C.^219^ S-Ti_3_C_2_ was employed for sunlight-driven CO_2_ reduction.^219^ The vulcanization process not only expands the NIR absorption capacity of the S-Ti_3_C_2_ catalyst but also enhanced its photothermal conversion efficiency. Under concentrated natural sunlight, this photocatalyst produced CH_4_ (12.03 mmol g^–1^ h^–1^) and C_2_H_4_ (3.55 mmol g^–1^ h^–1^), with a C_2+_ selectivity of 29.76%. Furthermore, the solar-to-carbon-fuel conversion efficiency exceeded 0.045%.^219^

A new composite material (NH_2_-UiO-66/Ti_3_C_3_T_*x*_) was synthesized via spontaneous self-assembly of Zr-MOF and Ti_3_C_3_T_*x*_ MXene layers.^225^ In this heterojunction, the role of Ti_3_C_3_T_*x*_ is to convert efficiently light into heat, while NH_2_-UiO-66 captures CO_2_ and promotes its reduction. Remarkably, this composite was capable of converting highly diluted atmospheric CO_2_ (418 ppm) into CO and CH_4_ at room temperature under simulated sunlight, using H_2_O as a reducing agent, yielding 140 and 241 ppm, respectively.^225^ However, it remains unclear why O_2_, a good electron acceptor, does not compete more favorably with CO_2_ reduction. Under simulated sunlight at 80 °C, the photocatalytic CO_2_ conversion to CO and CH_4_ reached 127 μmol g^–1^ h^–1^ and 330 μmol g^–1^ h^–1^, respectively.^225^ This rate was 4.76 times higher than the rate obtained for diluted atmospheric CO_2_. Interestingly, in contrast to thermocatalytic CO_2_ reduction processes typically requiring temperatures above 300 °C, this solid shows significant performance below 100 °C. This enhanced activity is attributed to the role of the MOF in providing additional active sites and lowering the activation energy. Further, the localized surface plasmon resonance effect of MXene facilitates charge carrier migration to the Zr^4+^ sites within the MOF. These observations were supported by DFT calculations.^225^ The composite retained its catalytic activity and product selectivity after five cycles.

Among various MXenes, carbonitride-based MXenes exhibit excellent electrical conductivity, high photothermal conversion, carrier mobility, and a larger number of exposed active sites, making them promising cocatalysts for photothermal reactions. In this regard, a Ti_3_CN/TiO_2_ heterojunction was prepared through *in situ* TiO_2_ growth from a Ti_3_CN precursor that led to much better performance than mechanical mixtures.^226^ The resulting heterojunction was evaluated for photothermal CO_2_ reduction. The ultrathin structure and enhanced exposure of surface-active sites provided a favorable platform for TiO_2_ growth, while the heterojunction prepared from monolayer Ti_3_CN exhibited stronger interfacial bonding, which promoted rapid carrier migration across the interface. SEM, XRD, XPS, and UV–Vis DRS characterization revealed that the monolayer-derived heterojunction exhibited a narrower bandgap and higher carrier mobility. The catalyst achieved a CO production rate of 11.36 μmol g^–1^ h^–1^ after 4 h, significantly outperforming P25 TiO_2_ (3 μmol g^–1^ h^–1^). This enhancement is attributed to the effective photothermal catalytic mechanism. Photothermal catalysis is not merely the sum of photocatalysis and thermal catalysis, but a synergistic process, as illustrated by the results shown in Figure 52. UV–Visible light excites charge carriers at TiO_2_, facilitating CO_2_ activation, while Visible-IR light provides heat at Ti_3_CN, which further promotes carrier separation, resulting in improved catalytic activity.^226^ The catalyst maintained its performance over five cycles without noticeable activity loss. However, it is worth noting that the study lacked ^13^C-labeling experiments to conclusively confirm the origin of the detected CO.

**Figure 52:**
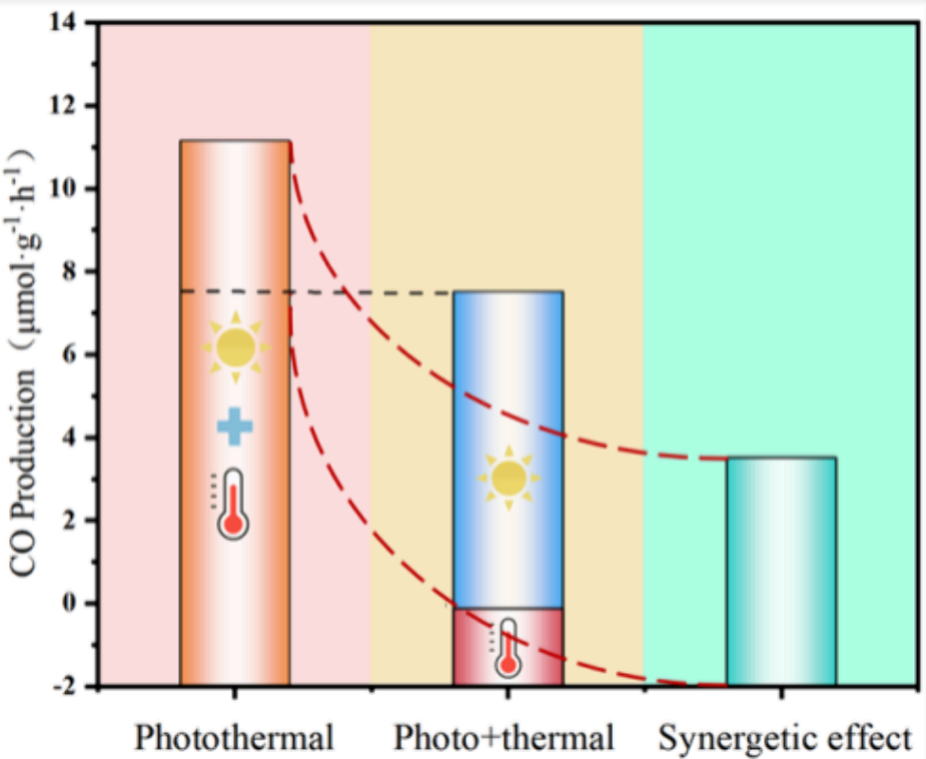
Quantification of the synergistic effect observed when comparing the activity of Ti_3_CN/TiO_2_ under photothermal with thermal (at the same temperature) and photocatalysis at ambient temperature under different catalytic conditions. Reproduced with permission from ref. 226. Copyright 2014 MDPI.

As previously discussed, photothermal catalysis is gaining increasing attention, particularly for CO_2_ hydrogenation, as the activity of photocatalysts may allow them to compete favorably with thermocatalytic processes that are nearing commercial implementation. However, a comprehensive understanding of the entire process remains lacking. In one attempt to elucidate mechanistic aspects, Ni­(OH)_2_/Ti_3_C_2_ (NiTC) composites were prepared by *in situ* hydrothermal synthesis of Ni­(OH)_2_ on preformed Ti_3_C_2_.^227^ β-Ni­(OH)_2_ was loaded on the surface interlayers of the accordion-like Ti_3_C_2_ structure, generating a built-in electric field at the Ni­(OH)_2_/Ti_3_C_2_ interface. The strong coupling between plasmonic Ti_3_C_2_ and the Ni­(OH)_2_ catalyst enabled the observation of surface plasmonic transverse electric and transverse magnetic resonances in Ni­(OH)_2_/Ti_3_C_2_, along with an enhanced absorption peak. Upon Vis–NIR light irradiation, a local surface plasmon resonance effect occurs in both Ti_3_C_2_ and Ni­(OH)_2_, and their spectral overlap enhances transverse magnetic wave generation. The resulting hot electrons enhance photothermal CO_2_ conversion. Under optimized conditions, the average CO production rate via photothermal CO_2_ hydrogenation over the Ni­(OH)_2_/Ti_3_C_2_ composite at 250 °C was 0.89 mmol g^–1^ h^–1^, which is 14 times higher than that of bare Ti_3_C_2_. The key point is how local heat facilitates hot electron generation and their involvement in the photothermal reaction.

One effective approach to mitigate the greenhouse effect is the direct capture and conversion of atmospheric CO_2_ into valuable fuels via photothermal catalysis. In this aspect, a CuTCPP/Ti_3_C_2_T_*x*_/TiO_2_ (CuTCPP: Cu tetra­(carboxyphenyl)­porphyrin that is a 2D metal–organic framework) catalyst was prepared in two steps: partial oxidation of Ti_3_C_2_T_*x*_ by hydrothermal treatment to form Ti_3_C_2_T_*x*_/TiO_2_, followed by *in situ* solvothermal synthesis of the 2D CuTCPP MOF.^220^ In this heterojunction, the role of Ti_3_C_2_T_*x*_ is to convert light into heat and ensure electrical conductivity among the components, while CuTCPP is the catalytically active component. Under optimized conditions, the CuTCPP/MXene/TiO_2_ composite exhibited CO and CH_4_ production rates of 124 μmol and 106 μmol g^–1^ h^–1^, respectively, significantly surpassing the performance of the individual components.^220^ The authors claim direct photocatalytic reduction of atmospheric CO_2_ by H_2_O, though the procedure for removing atmospheric O_2_ to avoid quenching of photogenerated electrons is not clearly explained. The enhanced performance is supported by DFT calculations indicating the generation of an internal electric field between components, promoting charge separation and utilization. Additionally, CuTCPP is reported to lower the free energy barrier of the photothermal reaction, while the local surface plasmon resonance and high electron transfer rates of MXene further accelerate the process. Although the catalyst maintained its activity over five cycles, some uncertainty remains regarding the ^13^CH_4_ detection due to potential interference from excess H_2_O during the labeling experiments.

MXene aerogels are promising multifunctional materials for developing efficient photocatalysts for CO_2_ reduction. Aerogels increase surface area, enhance substrate-catalyst interactions, improve electrical conductivity, and offer a self-supporting structure. However, pristine MXene aerogels exhibit poor light-harvesting capability and require photosensitizers for enhanced photocatalytic performance. Colloidal CsPbBr_3_ nanocrystals, with strong absorption in the UV–Visible region up to 550 nm, were supported onto Ti_3_C_2_T_*x*_ MXene aerogels (with T_*x*_ = −F, −O, −OH) and employed for CO_2_ reduction by H_2_O.^228^ Under optimal conditions, the CsPbBr_3_/Ti_3_C_2_T_*x*_ MXene aerogels achieved CO and CH_4_ production rates of 42 μmol g^–1^ h^–1^ and 3.5 μmol g^–1^ h^–1^, respectively, with no detectable H_2_, indicating selective CO_2_ reduction. This activity enhancement is attributed to (i) the high molar extinction coefficient of CsPbBr_3_ nanocrystals and the highly porous structure of MXene aerogels that improves light harvesting, (ii) open pores and hydrophilic surfaces that promote CO_2_ and H_2_O adsorption by providing abundant catalytic sites, and (iii) strong interfacial contact and excellent conductivity of MXene, enabling efficient charge separation and transport. The composite showed stable performance over eight cycles, attributed to effective immobilization of CsPbBr_3_, which prevented aggregation and deactivation. However, it is surprising that CsPbBr_3_ remained unaffected by moisture, given its known instability in humid environments in photovoltaic applications.^229^

V_2_C (VC) MXene has been employed as a support for Ni NPs and NiO nanosheet composites (Ni@NiO/VC), prepared by photocatalytic reduction of NiO_*x*_/VC (Figure 53). The Ni@NiO/VC catalyst was evaluated for photothermal CO_2_ hydrogenation to CH_4_.^138^ Under optimal conditions (H_2_/CO_2_ = 4/1, light intensity ∼ 1.9 W cm^–2^), the 0.8Ni@NiO/VC catalyst achieved 48.1% CO_2_ conversion with a CH_4_ production rate of 33.2 mmol g^–1^ h^–1^ and 99.2% selectivity under 300 W full-arc Xe lamp irradiation.^138^ Activity and selectivity were retained over ten consecutive cycles, indicating good stability in the conditions of photocatalytic CO_2_ reduction. As shown in Figure 53, the performance of 0.8Ni@NiO/VC under photothermal conditions significantly exceeded that under purely thermal conditions, highlighting the advantages of light-assisted catalysis. The enhanced activity and high CH_4_ selectivity are attributed to the large surface area, excellent photothermal properties of VC MXene, good interfacial contact, and the synergistic role of Ni and NiO in activating H_2_ and CO_2_ molecules.^138^

**Figure 53:**
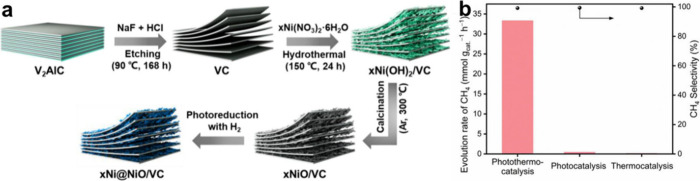
(a) Schematic illustration for the preparation of xNi@NiO/VC via *in situ* photoreduction with H_2_. (b) Comparison of the CH_4_ evolution rates over 0.8Ni@NiO/VC via photothermocatalysis (300 W Xe lamp), photocatalysis (300 W Xe lamp with water filter), and thermocatalysis (300 °C in the dark). Reproduced with permission from ref. 138. Copyright 2024 Elsevier.

In a recent study, V_4_C_3_-MXene crystals were prepared by HF etching of Al from V_4_AlC_3_.^217^ The harsh conditions of the etching process generate atomic vacancies that are associated with positive holes, which were quantified by EPR spectroscopy using 2,2,6,6-tetramethylpiperidine oxide as a probe, yielding 13.87 μmol g^–1^ of holes stored on the surface. Additionally, the presence of electrons stored inside the layers was quantified by methylene blue decolorization, resulting in 119.3 μmol g^–1^ of electrons.^217^ The density of these electrons and holes varies depending on the etching time. Upon exposure to full-spectrum sunlight irradiation, the electrons and holes in V_4_C_3_-MXene are excited, leading to resonant excitation that produces high-energy hot electrons and hot holes.^217^ The high-energy hot holes dissociate H_2_ into H^+^, while the hot electrons react with the adsorbed CO_2_ to form a series of intermediates, ultimately yielding CO. The stored holes and electrons in the V_4_C_3_-MXene crystal produce a resonance effect, raising the surface temperature to 369 °C and accelerating the photothermal catalytic conversion of CO_2_ to CO. Under the optimized reaction conditions, the CO_2_ photothermal conversion rate for V_4_C_3_-MXene reaches 95.68 μmol g^–1^ h^–1^, with a CO selectivity of 96% during CO_2_ reduction under simulated sunlight irradiation at 250 °C for 3 h, which is superior to thermal catalysis alone. This work nicely illustrates the synergistic influence of light and temperature in achieving efficient photothermal CO_2_ reduction and stands as one of the few reports where a MXene without additional metals or photosensitizers exhibits significant photothermal activity.

A Pt SA anchored over 3D hierarchical TiO_2_–Ti_3_C_2_ with atomic-scale interface engineering was prepared by first forming a 3D material with exfoliated Ti_3_C_2_ reduced with ascorbic acid, followed by partial oxidation to form TiO_2_–Ti_3_C_2_ via thermal treatment. Subsequent impregnation and photoreduction of H_2_PtCl_6_ yielded Pt/TiO_2_–Ti_3_C_2_ (Figure 54).^230^ The photocatalytic performance of this material was tested in the photoreduction of CO_2_ to CO. Interestingly, the *in situ* growth of TiO_2_ on Ti_3_C_2_ nanosheets provides an interfacial driving force for charge transport, while also creating an atomic-level charge transfer channel for directional electron migration.^230^ On the other hand, Pt anchored on TiO_2_ or Ti_3_C_2_ can effectively capture photogenerated electrons via atomic interfacial Pt–O bonds, shortening the charge migration distance and serving as active sites for CO_2_ adsorption and activation. Under optimized reaction conditions, the built-in interfacial electric field in Pt/TiO_2_–Ti_3_C_2_ contributes to its high photocatalytic performance for CO_2_ reduction to CO using H_2_O as both electron and proton donor, achieving a production rate of 20.5 μmol g^–1^ h^–1^ and a CO selectivity of 96%, which is five times higher than that of P25.

**Figure 54:**
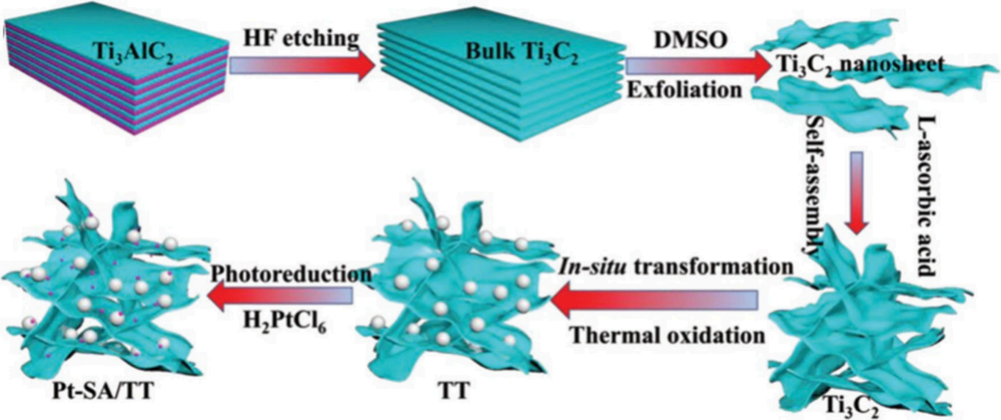
Schematic illustration of the synthetic process of Pt-SA/TT (Pt/TiO_2_–Ti_3_C_2_). Reproduced with permission from ref. 230. Copyright 2023 Wiley.

All in all, recent advances in CO_2_ photoreduction using MXene-based materials have demonstrated their promising potential in photothermal catalysis, particularly through synergistic interactions between light-induced thermal effects, active metal species, and engineered interfaces. MXenes, when integrated with metal NPs, metal oxides, MOFs, or photosensitizers, enable efficient CO_2_ activation and conversion under mild conditions, with several systems achieving high selectivity toward CO or CH_4_. Key factors contributing to enhanced performance include localized surface plasmon resonance, built-in electric fields, high thermal conductivity, and carrier mobility of MXenes. Moreover, materials such as Ti_3_C_2_, Mo_2_TiC_2_, V_4_C_3_, and Ti_3_CN have been successfully employed in diverse architectures, ranging from heterojunctions to aerogels, to improve light harvesting, charge separation, and reaction kinetics. Despite these encouraging results, challenges remain in mechanistic understanding, stability under operating conditions, and reliable isotope labeling to confirm carbon sources. Continued exploration of structure–function relationships and rational catalyst design will be essential to unlock the full potential of MXenes in solar-to-fuel conversion technologies.

### 6.3 Photothermal Hydrogen Evolution Reaction

Formic acid is considered a liquid hydrogen organic carrier, as it can release hydrogen on demand. In addition to thermal catalysis, one possibility to decompose formic acid into H_2_ and CO_2_ is via photocatalysis, offering the advantage of instantaneous H_2_ evolution.^231,232^ A report has studied the use of defective Ti_2_CT_*x*_ MXene nanosheets as a photocatalyst for solar-driven formic acid decomposition.^233^ To prepare the photocatalyst, Ti_2_CT_*x*_ was subjected to ultrasonic treatment, and the resulting suspension was dropped onto a sponge to form a defect-engineered MXene monolith (D-MM).^233^ Solar-driven formic acid decomposition by the D-MM solid exhibited a hydrogen generation rate of 401 mmol g^–1^ h^–1^ under one Sun irradiation from a 5 M aqueous formic acid solution, with complete H_2_ selectivity and catalytic stability over 45 h of operation, significantly surpassing many state-of-the-art photocatalysts, including Pd-based materials (Figure 55). This work provides new insights into the design of a photothermal MXene catalyst and may open new applications toward solar hydrogen generation from formic acid. The superior activity is attributed to interfacial heat localization and demonstrates a defect-engineering strategy for MXenes to integrate photothermal properties with catalytic activity. It should be noted, however, that Ti_2_CT_*x*_ is among the less stable MXenes as it is known to undergo oxidation very easily.

**Figure 55:**
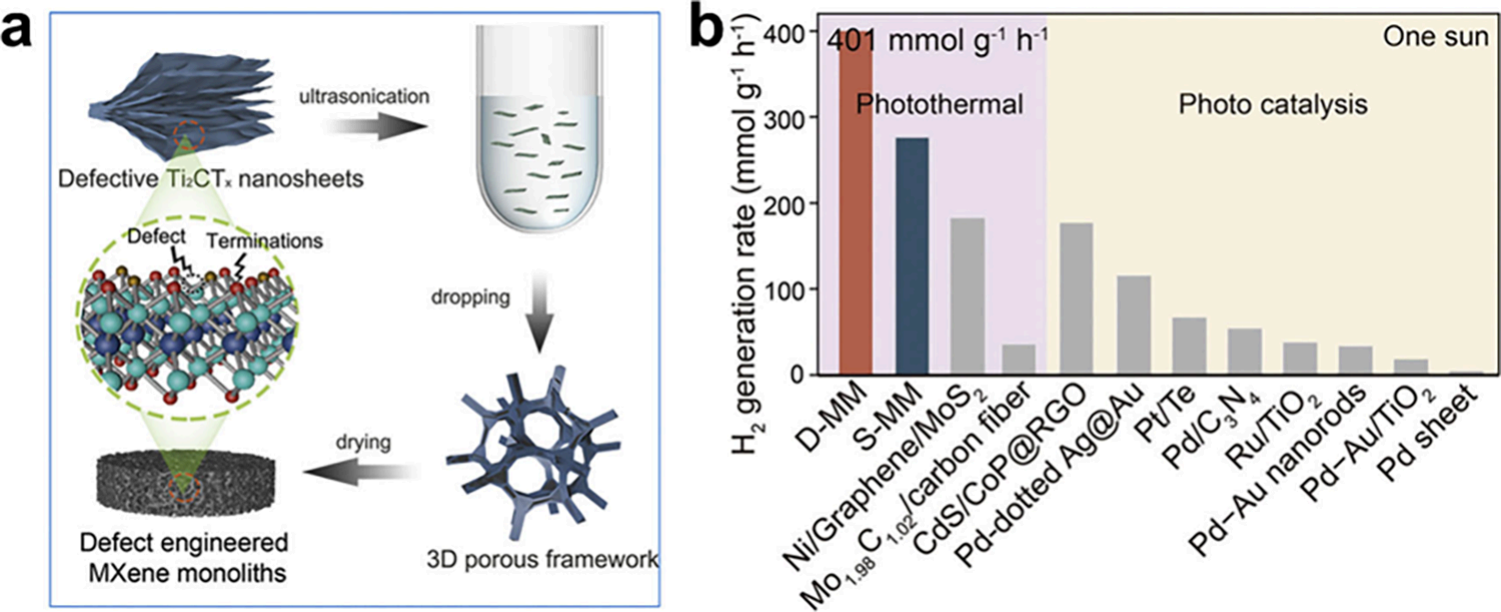
(a) Fabrication of the D-MM and (b) Comparison of H_2_ generation rates by formic acid decomposition over MXene monoliths and different photothermal and photocatalytic materials under 1 sun. S-MM stands for saturated MXene monolith. Reproduced with permission from ref. 233. Copyright 2022 Cell Press.

A two-dimensional multilayer accordion-like Ti_3_C_2_ with holes was obtained by a tiny-solvent-thermal method, in which it is claimed that the small amount of solvent and water, acting probably in the vapor phase, generates holes in Ti_3_C_2_ clay in which TiO_2_ grows *in situ* and uniformly (Figure 56).^234^ A hydrogen production rate of 7.28 mmol h^–1^ g^–1^ was achieved with Ti_3_C_2_–TiO_2_ containing 3 wt % Pt as a cocatalyst and triethanolamine (20 wt %) as a sacrificial electron donor under simulated sunlight irradiation. This photoactivity was 109 and 7 times higher than that of Ti_3_C_2_–TiO_2_ catalyst without holes and P25, respectively. The superior activity of the Ti_3_C_2_–TiO_2_ heterojunction is attributed to the efficient charge migration between the two components, narrower bandgap, high photogenerated charge separation efficiency, and fast transport rate. The electron storage characteristics of Ti_3_C_2_ can significantly suppress electron–hole recombination on TiO_2_ NPs and enhance electron accumulation in this component, favoring the occurrence of multielectron reactions. The hydrogen production efficiency of Ti_3_C_2_–TiO_2_ remained stable for three cycles, and the SEM image of the spent Ti_3_C_2_–TiO_2_ still showed the hole morphology (Figure 56), thus supporting its structural stability after three cycles.

**Figure 56:**
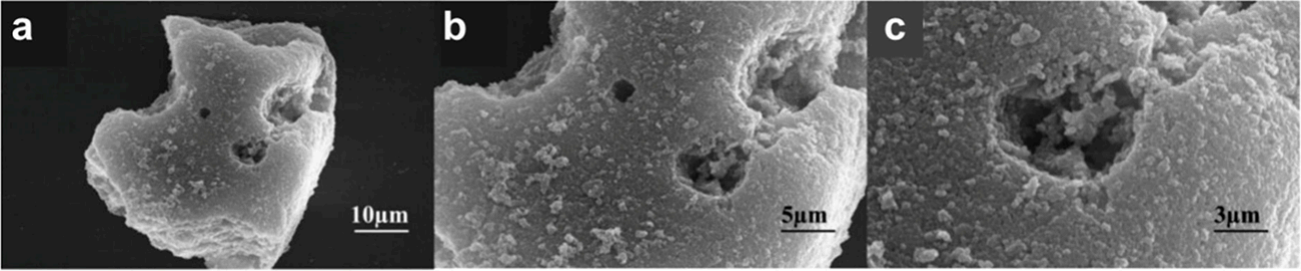
(a) SEM image of the fresh Ti_3_C_2_–TiO_2_, (b, c) partial enlargement of the hole after catalysis in consecutive cycles. Reproduced with permission from ref. 234. Copyright 2024 Elsevier.

The role of MXenes in most reports on photocatalysis consists in the formation of a Schottky junction with the semiconductor, which, after charge carrier migration across the interface, leads to the accumulation of electrons or holes in these materials.^235–237^ This process increases the efficiency of the photocatalytic process by suppressing wasteful charge recombination on the semiconductor. In addition, MXene can act as cocatalyst, facilitating the transfer of charge carriers to the substrates and promoting their transformation into key intermediates or products. In this context, it has been proposed that MXenes can perform similarly to noble metal NPs. However, these reports on the use of MXenes in photocatalysis to enhance photoinduced charge separation are outside the scope of the present review, which is focused on the role of MXenes in photothermal catalysis and the nature of the corresponding active sites. Nevertheless, in some cases, beyond the purely photoinduced charge transfer mechanism, it has been reported that the presence of MXenes can also open a photothermal pathway that operates simultaneously with conventional photoinduced charge separation and contributes to the overall photochemical reaction.

This is the case for a new heterostructure constructed by integrating Ti_3_C_2_ MXene quantum dots and a 3D porous graphitic carbon nitride (PGCN) via spontaneous electrostatic self-assembly to obtain Ti_3_C_2_QDs/PGCN (Scheme 12).^238^ Among the various photocatalysts prepared, 5.5 wt % Ti_3_C_2_ QD/PGCN composite exhibited 15.24 and 3.53 times higher photocatalytic H_2_ evolution rate using triethanolamine (10 wt %) as a sacrificial agent compared to pristine carbon nitride and PGCN, respectively.^238^ It was reported that this composite shows good photothermal conversion ability, accelerating hydrogen evolution with increasing temperature and enhancing light absorption and carrier density. However, although a photothermal effect, converting light into heat, was claimed in this study, it is unclear how a purely photothermal mechanism, implying a thermocatalytic process at elevated temperatures, could promote H_2_O splitting at 100 °C, even in the presence of triethanolamine as a sacrificial agent. Furthermore, EPR spectroscopy and DFT calculations confirm that the Schottky barrier between PGCN and Ti_3_C_2_ QD can efficiently promote spatial charge separation and significantly improve photocatalytic activity. Thus, it would be important to revisit the study to determine at which temperature hydrogen evolution for the Ti_3_C_2_QDs/PGCN heterojunction occurs in the dark.

**Scheme 12:**
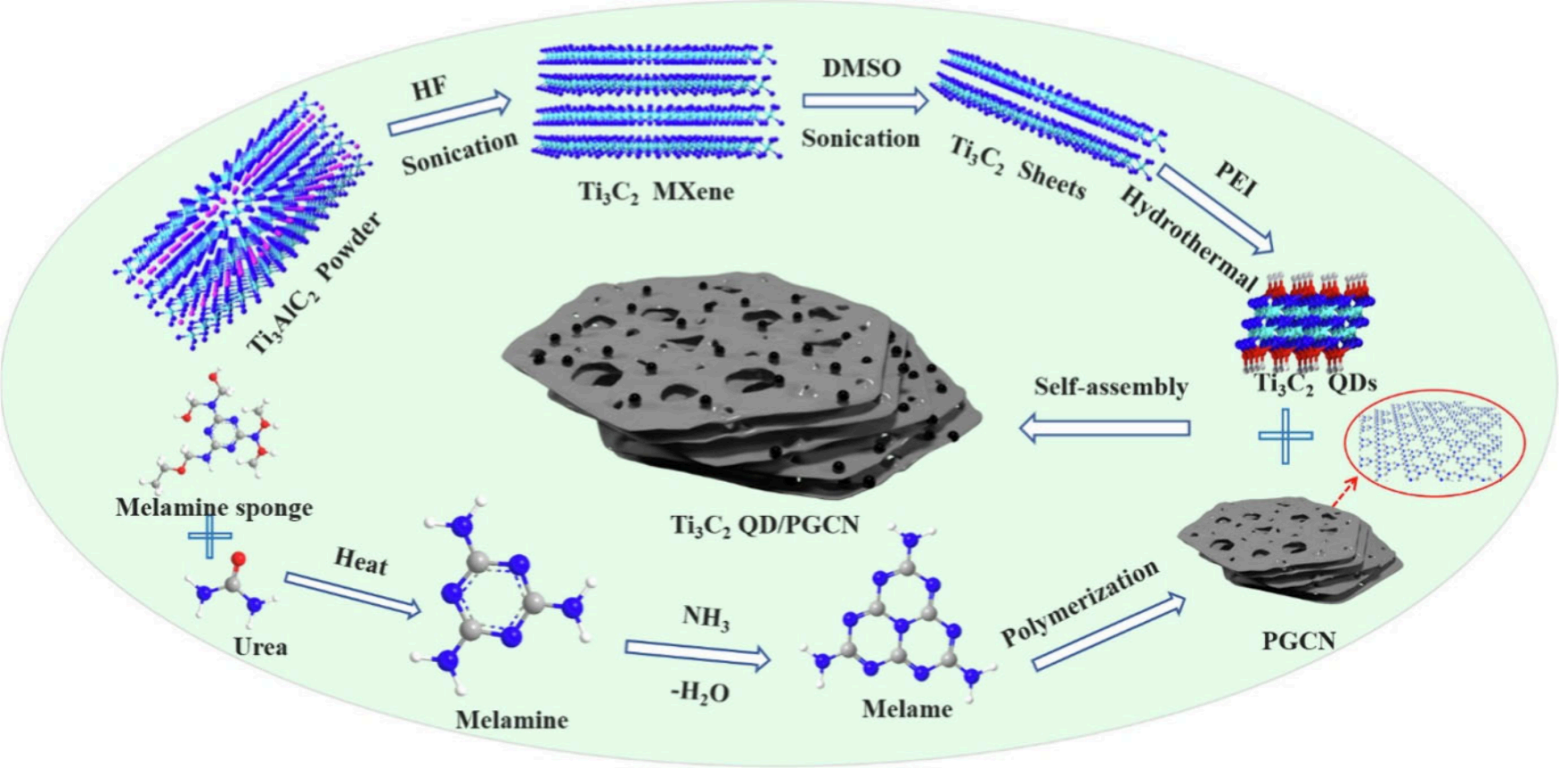
Illustration of the Preparation of Ti_3_C_2_ QD/PGCN Composite. Reproduced with permission from ref. 238. Copyright 2023 Elsevier

The construction of photocatalysts capable of absorbing the full solar spectrum, particularly NIR radiation through surface engineering, is an attractive strategy to fully harness solar energy in semiconducting materials. Since the energy of NIR photons is lower compared to that of the visible region, they can only excite narrow-gap electronic transitions, some of which do not possess the required energy to promote the target photochemical reaction. This makes the construction of efficient NIR-responsive photocatalysts very challenging.^239^

In this respect, as another example combining photoinduced charge separation and photothermal catalysis, a Ti_3_C_2_T_*x*_/CdS heterojunction was prepared through the *in situ* epitaxial growth of CdS nanosheets on the MXene surface using a solvothermal method.^240^ Under visible and NIR light irradiation, the composite showed a H_2_ evolution rate of 65.4 mmol g^–1^ h^–1^, which is 7.2 times higher than that of CdS alone. Interestingly, the composite catalyst exhibited a significantly higher surface temperature of 80.4 °C under visible light irradiation at an intensity of 0.1 W cm^–2^, which is 1.84 times higher than the value provided by CdS. Furthermore, the unique 2D/2D structure effectively mitigated the recombination of photogenerated carriers, enhancing the photocatalytic performance of the heterojunction. This catalyst was used for four cycles without any decay in its photocatalytic activity.

Ultrathin Ti_3_C_2_T_*x*_ nanosheets with high electrical conductivity and terminal −O– groups were self-assembled with an imine-linked COF (CTF-TFB, where CTF corresponds to covalent triazine framework and TFB to 1,3,5-triformylbenzene), and the resulting solid (CTF-TFB/Ti_3_C_2_T_*x*_) was studied for photocatalytic hydrogen evolution using ascorbic acid as sacrificial electron donor and Pt as a cocatalyst.^241^ Among the various conditions tested, the composite CTF-TFB/Ti_3_C_2_T_*x*_ exhibited about a 50% increase in apparent quantum yield at 420 and 450 nm compared to pristine CTF-TFB. This superior performance is attributed to the broad-spectrum light absorption and photothermal effect of Ti_3_C_2_T_*x*_ nanosheets, which significantly enhance the utilization of visible light by CTF-TFB. According to the proposed mechanism, photogenerated electrons from CTF-TFB migrate to Ti_3_C_2_T_*x*_, facilitating spatial charge separation, increasing charge separation efficiency, and extending their lifetime. In addition to the conventional photoinduced charge separation, the photothermal effect of Ti_3_C_2_T_*x*_ provides a surface temperature increase of approximately 10 °C, resulting in a further 67% enhancement in the photocatalytic hydrogen evolution rate in the composite—attributed to the contribution of photothermal pathway.

In summary, recent advances demonstrate the promising role of MXenes in photothermal hydrogen evolution reactions (HER), particularly for solar-driven decomposition of formic acid and water splitting. Defect-engineered and heterostructured MXene-based materials, such as Ti_2_CT_*x*_ monoliths and Ti_3_C_2_–TiO_2_ composites, exhibit high hydrogen production rates under simulated sunlight, benefiting from interfacial heat localization, efficient charge separation, and enhanced light absorption. Furthermore, MXenes often function as cocatalysts, forming Schottky junctions with semiconductors, promoting charge migration and suppressing recombination. In some systems, the photothermal effect contributes synergistically with photoinduced charge separation to boost H_2_ evolution, as evidenced in Ti_3_C_2_QDs/PGCN and Ti_3_C_2_T_*x*_/CdS heterojunctions. However, challenges remain in clearly distinguishing thermal and photothermal pathways, ensuring MXene stability, and validating reaction mechanisms, particularly under low-temperature or NIR irradiation. Advanced mechanistic studies and structural optimization will be key to fully realizing MXene potential in solar-to-hydrogen conversion.

### 6.4 Photothermal-Assisted Photocatalytic Nitrogen Fixation

Ammonia (NH_3_) synthesis from nitrogen is a cornerstone of the chemical industry, essential for fertilizer production and nitrogen-containing polymers, yet it remains one of the most energy-intensive industrial processes. The conventional Haber–Bosch process, operating under high temperature and pressure, accounts for approximately 1.5% of total global CO_2_ emissions.^242^ In line with current decarbonization goals, there is an urgent need to develop sustainable alternatives that rely on green hydrogen and renewable energy sources.^243^ While MXene-based systems for nitrogen fixation are generally classified within photothermal catalysis, most reported examples actually operate predominantly through photocatalytic pathways, in which photoexcited charge carriers drive the reduction of N_2_ to NH_3_. The accompanying photothermal effect, originating from localized surface plasmon resonance (LSPR) or broadband light absorption, mainly enhances the local temperature, accelerates surface reaction kinetics, and facilitates reactant activation, rather than serving as the primary driving force. Therefore, these reactions are more accurately described as photothermal-assisted photocatalytic processes.

Photocatalytic NH_3_ production could represent a sustainable strategy with the potential to mitigate energy consumption and environmental impact while addressing the growing demand for NH_3_ in agriculture, the chemical industry, and the energy sector.^244^ In this regard, photocatalytic NH_3_ production holds several advantages over traditional thermal Haber–Bosch synthesis, which is the standard industrial method. The current process requires extensive infrastructure and high capital costs, limiting its use to large-scale production facilities. In contrast, the photocatalytic approach commonly operates at ambient pressure and low temperatures and can be powered by renewable solar energy, reducing overall energy demand.^245^ A solar-powered photocatalytic system could potentially operate at low cost and allow for greater flexibility, enabling decentralized, smaller-scale on-site NH_3_ production.^245^

In certain respects, photocatalytic nitrogen fixation parallels the natural nitrogenase enzyme mechanism but utilizes solar energy to drive the reduction of N_2_ to NH_3_.^246^ In some studies, MXenes serve as cocatalysts or electron mediators in photocatalytic systems, improving charge separation in semiconductors by acting as efficient electron acceptors and relays, while also introducing a thermal effect. The high electrical conductivity of some MXenes is believed to facilitate rapid electron transfer and charge migration, partially suppressing the recombination losses of photogenerated electron–hole pairs in single semiconductor photocatalysts. Furthermore, the 2D morphology of MXenes is well-suited to establishing strong interfacial interactions with semiconductors, enhancing charge separation efficiency and boosting overall photocatalytic performance.^247^

The integration of MXenes into photocatalytic systems has advanced the field of sustainable NH_3_ synthesis.^247^ MXenes have demonstrated remarkable catalytic potential, particularly in NH_3_ synthesis.^247^ In addition to their intrinsic properties, such as high electrical and thermal conductivity and tunable surface groups, certain MXenes exhibit strong N_2_ activation capability, outperforming many traditional catalysts in terms of efficiency and stability. Recent advancements in MXene-based catalysts include defect engineering to increase the density of active sites, heterostructure formation to organize components and facilitate charge migration, and synergistic interactions with metals and semiconductors to enhance nitrogen activation and charge carrier dynamics, ultimately leading to significant improvements in NH_3_ production via sustainable routes.

Certain metal NPs, such as Au NPs, exhibit a LSPR absorption band in the visible region. When supported on MXenes, these metal NPs enhance light absorption and generate hot electrons that are efficiently trapped on the MXene surface, producing photothermal effects. This plasmonic enhancement facilitates charge accumulation at catalytic sites, driving NH_3_ synthesis using H_2_O as a reducing agent under 400–780 nm light irradiation, at an exceptional rate of 5334 μmol g^–1^ h^–1^ while maintaining high selectivity and stability.^248^ Nitrogen adsorption occurs through both end-on chemisorption on Ti sites of MXene and side-on adsorption at oxygen vacancies of reduced Ti_3_C_2_. It was proposed that, upon visible light exposure, photoexcited electrons from Au NPs provide a high reduction potential, allowing N_2_ molecules to form transient states (*N_2_^•–^) and enter vibrationally excited states. This process weakens the nitrogen–nitrogen triple bond, facilitating electron transfer and subsequent hydrogenation into NH_3_. However, it should be noted that the electron affinity of N_2_ is highly negative, and the process, in the absence of a concurrent proton-coupled transfer, is thermodynamically unfavorable.

MXene-based photocatalysts are not limited to visible-light-driven processes but also operate in the NIR region due to their broadband absorption. Ti_3_C_2_T_*x*_/TiO_2_-400, a hybrid plasmonic catalyst, utilizes the ability of Ti_3_C_2_T_*x*_ MXene to harvest NIR light, generating hot electrons that facilitate NH_3_ synthesis, reaching a production rate of 82 μmol g^–1^ h^–1^ under 740 nm irradiation and 422 μmol g^–1^ h^–1^ under full-spectrum illumination.^249^ Figure 57 summarizes the photocatalytic activity of this plasmonic photocatalyst. Isotopic ^15^N-labeling experiments are always advisible to confirm N_2_ as the source of the detected NH_3_.

**Figure 57:**
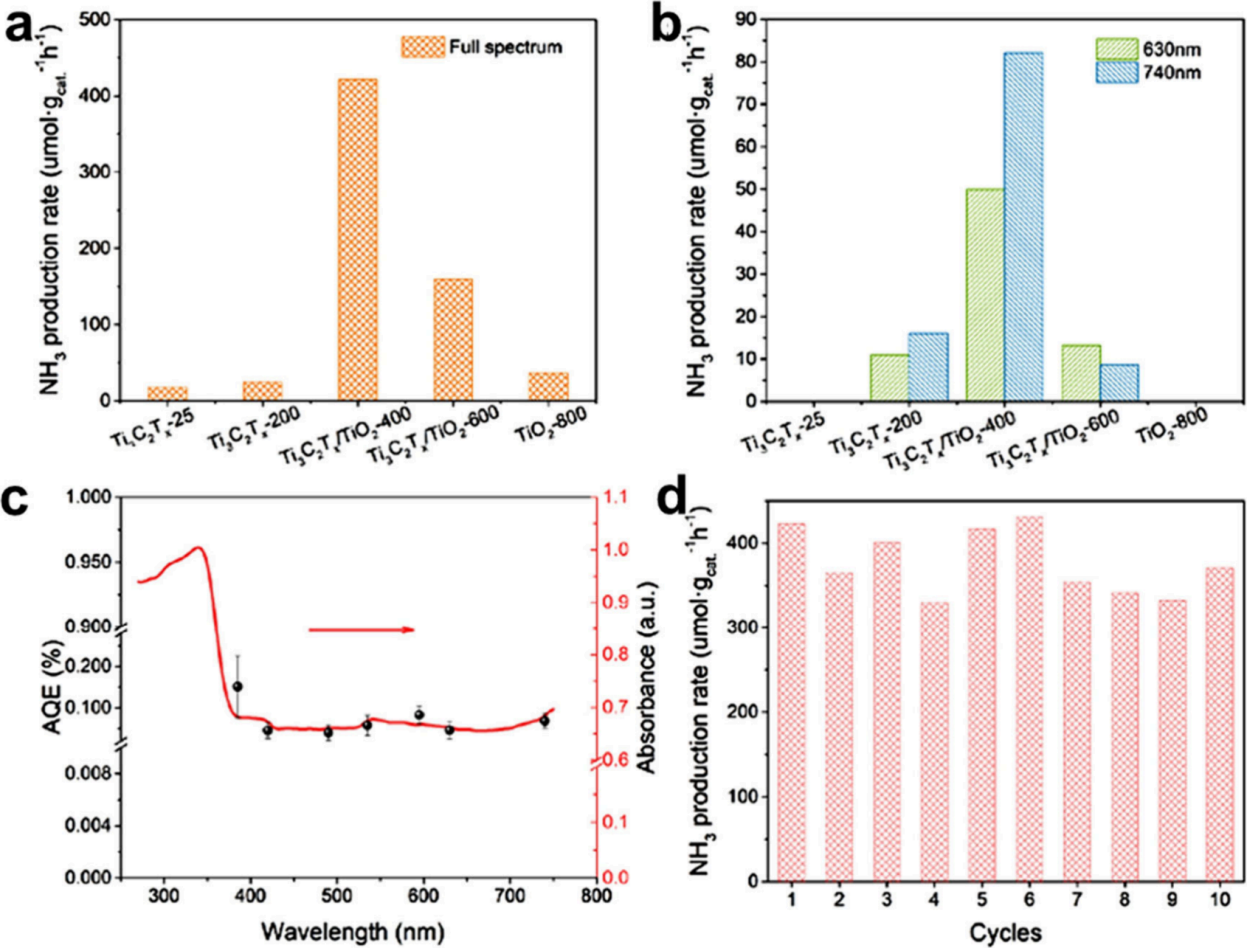
Photocatalytic NH_3_ production rates over Ti_3_C_2_T_*x*_-25 (after Ti_3_AlC_2_ etching at room temperature), Ti_3_C_2_T_*x*_-200 (Ti_3_C_2_T_*x*_-25 heated in air at 200 °C, whereby the MXene structure remains), Ti_3_C_2_T_*x*_/TiO_2_-400 (Ti_3_C_2_T_*x*_-25 heated in air at 400 °C, whereby a partial conversion to TiO_2_ occurs), Ti_3_C_2_T_*x*_/TiO_2_-600 (same as previous sample heated at 600 °C), and TiO_2_-800 (Ti_3_C_2_T_*x*_-25 heated in air at 800 °C, whereby a complete conversion to TiO_2_ occurs) under irradiation of (a) full spectrum of xenon lamp and (b) irradiation with monochromatic light of 630 and 740 nm wavelength. (c) Calculated apparent quantum efficiency values for N_2_ fixation over Ti_3_C_2_T_*x*_/TiO_2_-400 under monochromatic light irradiation. (d) NH_3_ production rate of Ti_3_C_2_T_*x*_/TiO_2_-400 under irradiation of full spectrum of xenon lamp over the course of ten rounds of successive reaction. Reproduced with permission from ref. 249. Copyright 2020 Elsevier.

End-on chemisorption and side-on chemisorption are two distinct ways in which nitrogen molecules can bind to a catalyst surface during nitrogen fixation processes. These modes determine how the catalyst interacts with N_2_ and facilitates its activation. In end-on chemisorption, the nitrogen molecule (N_2_) binds to the surface of the catalyst through one of its nitrogen atoms, with the molecule oriented perpendicular to the surface. This typically involves a strong interaction between a metal atom on the catalyst surface and the lone pair of electrons on one nitrogen atom. The catalyst donates electrons to the antibonding orbitals of N_2_, weakening the N≡N triple bond and facilitating its reduction.^250^ This type of binding is commonly observed in systems where nitrogen binds to d-block transition metals such as Fe, Ti, or synthetic catalysts containing small NPs.

In side-on chemisorption, the nitrogen molecule binds to the surface with both nitrogen atoms interacting with the catalyst, and the molecule lies parallel to the surface. In this case, both nitrogen atoms interact with the catalyst, typically via π-back bonding or through coordination with surface oxygen vacancies. Electrons from the catalyst tend to weaken the N≡N bond by populating antibonding π* orbitals. The electron density is evenly distributed across both nitrogen atoms, making the breaking of the N≡N bond easier. This mode is typically observed in catalysts with active sites such as vacancies or coordinatively unsaturated metal atoms.

Synergistic effects between MXenes and semiconductor or metal components further improve catalytic performance by enhancing light absorption beyond the UV and even into the NIR region, increasing charge separation, and enabling efficient surface area utilization.^251^ Heterostructures such as Ti_3_C_2_ MXene coupled with BiOBr^252^ or CdS^253^ ensure optimal active site availability while preserving structural integrity. These findings collectively highlight the transformative potential of MXenes in photocatalysis for N_2_ reduction and suggest that future research should focus on further tuning the nature and density of surface defects, rational interfacial engineering, and multicomponent hybridization to push the boundaries of NH_3_ synthesis efficiency.

Overall, photothermal-assisted photocatalytic nitrogen fixation using MXene-based materials represents a promising pathway for sustainable NH_3_ synthesis under mild conditions, offering an alternative to the energy-intensive Haber–Bosch process. By leveraging the high electrical conductivity, tunable surface chemistry, and photothermal properties of MXenes, significant progress has been made in enhancing light absorption, promoting charge separation, and facilitating N_2_ activation. Strategies such as plasmonic enhancement, defect engineering, and interfacial coupling with semiconductors or metals have enabled efficient NH_3_ production using visible and NIR light. However, challenges remain in confirming the reaction mechanism, ensuring catalyst stability, and scaling up the process. To advance toward truly pure photothermal nitrogen fixation, future research should focus on designing metallic or plasmonic MXenes capable of converting photon energy entirely into localized heat, thereby driving N_2_ activation and hydrogenation through purely thermal pathways. Such solar-thermal catalytic systems could ultimately bridge the gap between photothermal and thermocatalytic NH_3_ synthesis.

### 6.5 Environmental Remediation and Pollutant Degradation: Reactive Oxygen Species, Thermal Activation Pathways

Dehalogenation and dehydrogenation reactions play a pivotal role in environmental remediation, particularly in the degradation of halogenated organic pollutants and the conversion of organic compounds into value-added chemicals. Halogenated contaminants, such as chlorinated solvents, pesticides, and pharmaceuticals, are persistent and toxic, posing serious ecological and health hazards. Their removal often requires reductive pathways under mild and selective conditions. Similarly, dehydrogenation is a fundamental step in the transformation of saturated hydrocarbons into olefins and aromatic compounds and is increasingly relevant in clean energy applications such as hydrogen release from liquid organic hydrogen carriers (LOHCs). MXene-based catalysts have emerged as promising candidates for both reactions, owing to their tunable surface chemistry, high electron mobility, and strong MSIs. These features enable MXenes to efficiently activate C–X (X = Cl, Br) and C–H bonds, particularly when combined with reactive oxygen species or under photothermal/thermal conditions, offering versatile platforms for pollutant detoxification and sustainable chemical conversions.

Perfluorooctanesulfonate (PFOS) is a fluorinated synthetic surfactant frequently used in consumer-end products, but it has raised recent concern due to its environmental persistence and toxicity. One of the strategies to promote the degradation of PFOS through C–F bond cleavage is the use of solvated electrons in water via reductive processes. Unfortunately, solvated electrons are easily scavenged, resulting in poor or incomplete defluorination. To overcome these issues, one approach is to develop heterogeneous solid catalysts capable of efficiently degrading the C–F bonds in PFOS. However, currently available solid catalysts are still not very effective. In this regard, Ray and co-workers reported the catalytic performance of V_2_C MXene nanosheets in the defluorination of PFOS using H_2_O_2_ at room temperature under aerobic conditions (Figure 58).^254^ Under optimized conditions, 96% removal of PFOS was achieved after 4 h using V_2_C nanosheets as catalysts for H_2_O_2_-assisted defluorination. In contrast, the activity of V_2_C alone (62% removal) or H_2_O_2_ alone (no removal) was considerably lower under identical conditions. The authors hypothesize that solvated electrons, generated by the interaction between V_2_C and H_2_O_2_, are responsible for the rapid defluorination of PFOS adsorbed on the V_2_C surface.^254^ However, additional spectroscopic characterization would be necessary to provide compelling evidence for the actual active species involved in the cleavage of C–F bonds in PFOS.

**Figure 58:**
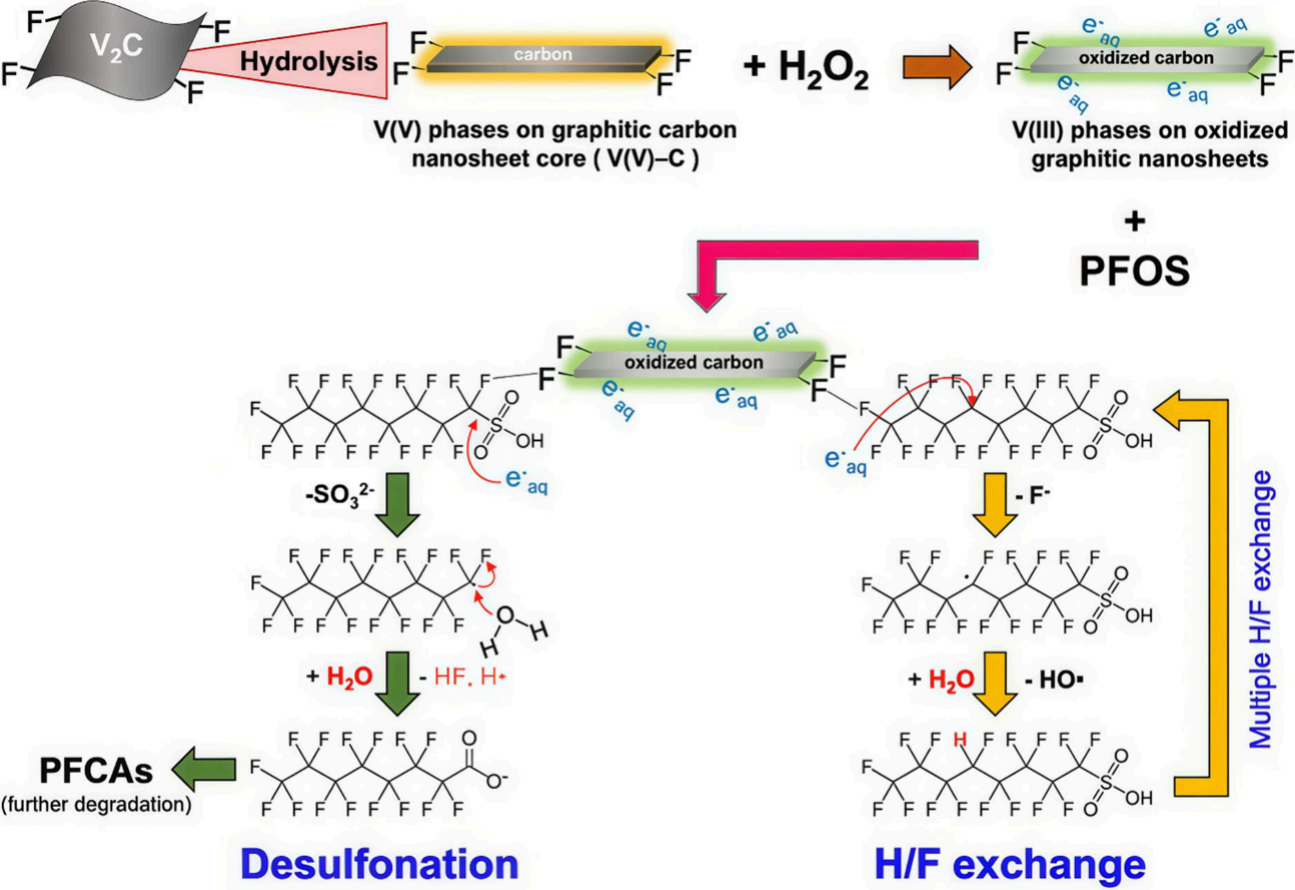
Proposed formation of V_2_C-derived V­(V) phases on graphitic carbon by reaction with H_2_O_2_ that also charges the material with solvated electrons that facilitate PFOS degradation and defluorination. Reproduced with permission from ref. 254. Copyright 2023 Royal Society of Chemistry.

In sum, MXene-based catalysts have emerged as highly promising materials for environmental remediation, especially in the degradation of persistent organic pollutants. Their ability to activate oxidants like H_2_O_2_ and persulfates enables the generation of reactive oxygen species, including ^•^OH, O_2_^–•^, and ^1^O_2_, which are instrumental in the oxidative degradation of toxic compounds. Beyond oxidation, MXenes and derived materials also demonstrate potential in reductive transformations such as dehalogenation and defluorination. Notably, V_2_C MXene upon transformation has been shown to promote efficient PFOS defluorination under ambient conditions via a proposed solvated electron mechanism, although further mechanistic insights are still required. These findings illustrate the dual oxidative and reductive versatility of MXenes in pollutant degradation and highlight the need for further studies on long-term stability, selectivity, and mechanistic pathways to fully realize their environmental applications.

## 7 Challenges and Future Directions

Since the first report in 2011, MXenes have become intensively studied in many areas driven by the unique structural and physicochemical properties of these 2D metal carbides. These properties include mechanical and thermal stability as well as electrical and thermal conductivity, among others. Over the past decade, these materials have become widely used for electromagnetic shielding, for charge storage in supercapacitors, and in important electrocatalytic processes, among other applications. Since heterogeneous catalysis makes ample use of transition metal compounds and MXenes have clear similarities with some molecular metal complex catalysts and with bulk metal carbides and nitrides, it can be expected that MXenes will be increasingly used also as thermal and photothermal catalysts.

It has been shown how the current MXene synthesis methods unavoidably introduce surface terminations and generate defects that can behave as active centers to promote chemical reactions. These structural sites can be Brönsted or Lewis acid and basic sites and they can also be active for reduction reactions. It has been commented that the oxyphilic nature of the early transition metals and the low average oxidation state of the M element, make MXenes prone to undergo oxidation to the corresponding metal oxide. This lack of stability under oxidative environments remains a major challenge hampering their broader catalytic deployment, particularly for oxidation reactions, but it is expected to be gradually mitigated through improved surface engineering and protective strategies.

Besides structural active sites, the review has conveniently highlighted the advantages of the 2D morphology and atom vacancies in MXenes for their use as supports, particularly for the preparation of SA catalysts and to establish reactive metal support interactions with the supported catalysts. In both cases, MXenes compete with the best materials as supports. In one case, MXenes provide atomic nests for SAs generated during the etching process. In the second case, the M metal of the MXene and the supported metal form at the interface an intermetallic phase that binds strongly the supported metal to the MXene structure while providing additional tuning to the electronic properties of the metal sites.

In addition to the lack of stability against oxidation, another point of concern related to the preparation procedures is the possible variability of the catalytic activity among various batches, making it recommendable to provide a detailed description of all the relevant experimental synthetic details and to provide a comparison of the catalytic performance among different independent batches prepared under identical conditions. Best experimental practices are especially important in the current state of the art of the use of MXenes in heterogeneous catalysis as it is also a proper comparison of their catalytic activity with benchmark catalysts. This can be done through a correct determination of the TOF values based on accurate initial reaction rates and a reasonable assumption about the nature of the active sites and their population. From the scarce TOF data available, it seems that MXenes are efficient catalysts as solid acids to promote amine additions, but also for hydrogenations, dehydrogenations, and hydrodeoxygenations, following in the last reactions the expected catalytic activity of bulk transition metal carbides and nitrides, but with additional benefits arising from reduced dimensionality.

There is, however, an issue on how to control the density and nature of the active sites. It seems that the current understanding of the structure and synthesis of MXenes indicates that certain parameters, such as the concentration of HF and etching time, allow for the generation of defects and atom vacancies in a variable density. Also, postsynthetic treatments can be used to modify the surface functional groups or the carbide layer. Thus, it seems that the combination of appropriate synthetic protocols and subsequent post-treatments will have a considerable impact on the catalytic activity of MXenes, if they are properly designed and carried out. Systematic studies correlating catalytic performance with specific structural features introduced during synthesis and post-treatment remain essential for guiding rational design.

At the moment, Ti_3_C_2_ is by far the most studied and widely employed MXene, mainly due to the commercial availability of the MAX precursor and the convenient and reliable etching process, which is often the limiting factor hampering the use of other MXenes. The properties of Ti_3_C_2_ in terms of oxyphilicity and reducibility are clearly different from those of MXenes of other transition metals, like Mo_2_C. Expanding catalytic studies to the vast MXene chemical space is a target in the area, since contrasting catalytic performances are predicted by theory. Also, a broader scope of reactions that can be promoted by MXenes, including photothermal processes, can also be anticipated, particularly as the field is expanding outside hydrogenation-type reactions. Multifunctional MXenes exhibiting sites of different nature can serve to implement tandem reactions, as it is well established for other materials. Particularly, the combination of structural acid sites with supported metals appears to be a doable strategy for tandem reactions, offering the additional advantage of strong metal anchoring or SA dispersion on the MXene surface.

As it has occurred in other areas, DFT calculations can provide valuable information about the nature of the active sites and reaction mechanisms, proposing the most efficient surface terminations and defects and giving a predictive rationalization of the catalytic results. It is expected that most catalytic studies will include calculation on appropriate models, probably implementing machine learning algorithms and artificial intelligence. With proper development, these tools will be used in the design of the most efficient MXenes for each reaction based on the reaction mechanism. *In situ* and *operando* spectroscopic techniques will provide experimental support to these proposed mechanisms, validating in this way the results from calculations. Vibrational spectroscopy, particularly Raman spectroscopy, can serve to determine alterations of the surface functional groups in the presence of substrates and reagents and also to detect reaction intermediates predicted by theory.

The final goal of materials in heterogeneous catalysis is always to implement advantageous, competitive industrial processes based on them. In the case of MXenes, it seems that their use as thermal catalysts is still far from this ambition. However, the development of new MXene synthesis methods, applicable on a large scale and with minimal negative environmental impact, would be necessary, if the situation materializes. Driven by other applications, particularly in the field of batteries and supercapacitors, it appears that large-scale preparation methods are being developed and the cost of MXenes has considerably diminished. Also considering the current interest of heterogeneous catalysis, with strong focus on renewable energies and processes related to circular economy, recycling and sustainability, it can be expected that the activity of MXenes for these new processes will continue to be explored. Selective CO_2_ hydrogenations and N_2_ fixations are, in fact, already among the most studied reactions with MXenes as catalysts, and it is expected that these studies will continue. These reactions are especially appealing to be carried out under photothermal conditions, since the combination of heat and light provides temperature and hot electrons favoring the process under advantageous conditions compared to the conventional purely thermal reaction. In addition, it opens the door for the direct use of natural sunlight for some of these reactions.

From a scalability perspective, most laboratory-scale MXene synthesis routes, particularly those based on HF or LiF-HCl etching, remain unsuitable for large-scale or continuous production due to safety and waste-management concerns. Nevertheless, recent advances in molten-salt etching, electrochemical delamination, and fluorine-free chemical routes have demonstrated gram-to-kilogram-level scalability with improved environmental compatibility, suggesting a clear pathway toward industrial adoption. Moreover, mechanical milling, extrusion, and spray-drying techniques have shown promise for assembling MXene-based composites and catalysts at scale, potentially bridging the gap between laboratory synthesis and pilot-scale applications.

In terms of technology readiness, MXene-based catalysts currently remain at low-to-mid Technology Readiness Levels (TRLs), corresponding to proof-of-concept and small prototype demonstrations. However, specific applications, such as MXenes serving as conductive supports for single-atom catalysts or as photothermal mediators in CO_2_ hydrogenation and N_2_ fixation, are approaching higher TRLs as they demonstrate operational stability, reproducibility, and scalability under semicontinuous conditions. With ongoing progress in large-scale synthesis, durability enhancement, and process integration, these systems are expected to advance toward pilot testing and integration into solar-driven catalytic modules in the near future.

In summary, it is clear that the use of MXenes as heterogeneous catalysis is still in its infancy, but with a considerable potential for development based on the understanding of the nature of the possible active sites. Bottlenecks related to the synthesis, limited reproducibility and the tendency to undergo oxidation have been identified as challenges to be overcome. Future directions related to broadening the composition of MXenes used in catalysis beyond Ti_3_C_2_ and Nb_2_C, broadening the reaction types besides hydrogenations, further exploration of photothermal effects, and the possible prediction and design of each component of the MXene structure to optimize their activity using artificial intelligence and theory have been identified as important research topics to fully exploit MXenes as heterogeneous thermal catalysts. Equally important, addressing scalability and technology-readiness challenges through sustainable synthesis and continuous manufacturing will be decisive for translating MXene-based catalytic concepts from laboratory research to industrially relevant processes. It is likely that, driven by other applications, many different MXenes will become commercially available, even in large quantities, and this will also have an impact on their use in heterogeneous catalysis, potentially enabling large scale industrial catalytic processes.
